# Limited survival and impaired hepatic fasting metabolism in mice with constitutive Rag GTPase signaling

**DOI:** 10.1038/s41467-021-23857-8

**Published:** 2021-06-16

**Authors:** Celia de la Calle Arregui, Ana Belén Plata-Gómez, Nerea Deleyto-Seldas, Fernando García, Ana Ortega-Molina, Julio Abril-Garrido, Elena Rodriguez, Ivan Nemazanyy, Laura Tribouillard, Alba de Martino, Eduardo Caleiras, Ramón Campos-Olivas, Francisca Mulero, Mathieu Laplante, Javier Muñoz, Mario Pende, Guadalupe Sabio, David M. Sabatini, Alejo Efeyan

**Affiliations:** 1 Metabolism and Cell Signaling Laboratory, Spanish National Cancer Research Centre (CNIO), Madrid, Spain; 2 Proteomics Unit, Spanish National Cancer Research Centre (CNIO), Madrid, Spain; 3 Myocardial Pathophysiology, Centro Nacional de Investigaciones Cardiovasculares (CNIC), Madrid, Spain; 4 Platform for Metabolic Analyses, Structure Fédérative de Recherche Necker, INSERM US24/CNRS UMS 3633, Paris, France; 5 Centre de recherche de l’Institut universitaire de cardiologie et de pneumologie de Québec (CRIUCPQ), Faculté de Médecine, Université Laval, Québec, QC Canada; 6 Centre de recherche sur le cancer de l’Université Laval, Université Laval, Québec, QC Canada; 7 Histopathology Unit. Spanish National Cancer Research Centre (CNIO), Madrid, Spain; 8 Spectroscopy and NMR Unit, Spanish National Cancer Research Centre (CNIO), Madrid, Spain; 9 Molecular Imaging Unit, Spanish National Cancer Research Centre (CNIO), Madrid, Spain; 10 Institut Necker Enfants Malades, INSERM U1151, Université de Paris, Paris, France; 11 Whitehead Institute for Biomedical Research, Nine Cambridge Center, Cambridge, MA USA; 12 Department of Biology, Massachusetts Institute of Technology (MIT), Cambridge, MA USA; 13 David H. Koch Institute for Integrative Cancer Research at MIT, Cambridge, MA USA; 14 Broad Institute, Seven Cambridge Center, Cambridge, MA USA; 15 Howard Hughes Medical Institute, MIT, Cambridge, MA USA

**Keywords:** Kinases, Endocrine system and metabolic diseases

## Abstract

The mechanistic target of rapamycin complex 1 (mTORC1) integrates cellular nutrient signaling and hormonal cues to control metabolism. We have previously shown that constitutive nutrient signaling to mTORC1 by means of genetic activation of *RagA* (expression of GTP-locked RagA, or RagA^GTP^) in mice resulted in a fatal energetic crisis at birth. Herein, we rescue neonatal lethality in *RagA*^GTP^ mice and find morphometric and metabolic alterations that span glucose, lipid, ketone, bile acid and amino acid homeostasis in adults, and a median lifespan of nine months. Proteomic and metabolomic analyses of livers from *RagA*^GTP^ mice reveal a failed metabolic adaptation to fasting due to a global impairment in PPARα transcriptional program. These metabolic defects are partially recapitulated by restricting activation of RagA to hepatocytes, and revert by pharmacological inhibition of mTORC1. Constitutive hepatic nutrient signaling does not cause hepatocellular damage and carcinomas, unlike genetic activation of growth factor signaling upstream of mTORC1. In summary, RagA signaling dictates dynamic responses to feeding-fasting cycles to tune metabolism so as to match the nutritional state.

## Abstract (web summary)

The mechanistic target of rapamycin complex 1 (mTORC1) integrates nutrient and hormonal signals to control metabolism. Here the authors investigate the effects of constitutive nutrient signaling through genetic activation of RagA in adult mice and show that constitutive nutrient signaling regulates the response to feeding-fasting cycles and does not drive liver cancer.

## Introduction

Multicellular organisms adjust their metabolism to the fluctuations of internal and environmental nutrient levels. Insulin and other hormones act as second messengers of systemic nutrient sufficiency to ensure coordinated, coherent responses in different organs to uptake, consume and store during nutrient surpluses. At the cellular level, hormonal cues are integrated with those stemming from the detection of nutrients in the cell to adjust anabolism and catabolism accordingly to both systemic and local abundance^1^. In response to cues from hormones and cellular nutrients, the mechanistic target of rapamycin (mTOR) kinase complex 1 (mTORC1) controls cellular anabolic responses including protein, RNA, nucleotide and lipid synthesis^2^. The signal transduction cascade emanating from insulin binding its receptor at the plasma membrane involves the activation of PI3K and AKT, which leads to the inhibitory phosphorylation of the tuberous sclerosis complex (TSC), itself a GTPase activating protein (GAP) toward Ras homology enriched in brain (RHEB), responsible for direct binding and kinase activation of mTORC1^3^. The involvement of the PI3K-AKT-TSC axis in human disease, including cancer and syndromes related to deregulated control of mTORC1, has fostered the development of mouse models with deregulated hormonal input to mTORC1, being the prime example *TSC1/2* knock out mice^4–6^. *TSC1/2* knock-out mice die embryonically with supra-physiological mTORC1 activity, and tissue-specific models of deregulated hormonal signaling to mTORC1 have taught us its relevance in coordinating fasting-feeding responses, and in driving pathological growth^7–10^.

The cellular nutrient signaling cascade that activates mTORC1 occurs independently of that of growth factors, and starts with the activation of sensors of different nutrients, including arginine, leucine, cholesterol, glucose, and other molecules^11–18^. The signals are ultimately transduced to the Rag family of GTPases, which includes RagA, B, C and D, tethered to the outer lysosomal surface, where amino acid sensing occurs in an inside-out manner^19^. The Rag GTPases operate as heterodimeric complexes formed by either RagA or RagB plus either RagC or RagD. Nutrient sufficiency results in loading of RagA with GTP and RagC with GDP, nucleotide configuration that drives recruitment of mTORC1 to the outer lysosomal surface where the Rag and Rheb GTPases reside, and hence mTORC1 activation occurs^1,20^. The detailed information regarding the molecular architecture of the regulation of mTORC1 by cellular nutrients contrasts with the fragmented information on its relevance in mammalian physiology. To interrogate on the physiological perturbations caused by systemic chronic elevation in nutrient signaling we have previously generated a gain-of-function model of nutrient signaling to mTORC1 with knock-in mice expressing a GTP-bound, constitutively-active form of RagA under endogenous control of expression^15^. *RagA*^GTP/GTP^ mice developed normally but succumbed within hours after birth due to a profound metabolic crisis that manifested with decreased circulating levels of amino acids and hypoglycaemia. This metabolic crisis was caused by the inability of cells with constitutive nutrient signaling to inhibit mTORC1 during the early neonatal starvation period and, consequently by an impairment in the execution of nutrient-recycling autophagy^15^.

In this work, we rescued neonatal lethality and produced adult mice with full-body expression of RagA^GTP^ to understand the physiological consequences of constitutive nutrient signaling to mTORC1 in adult mice. Adult *RagA*^GTP^ mice constitute a genetic model of chronically elevated nutrient signaling to mTORC1, without experimental perturbations in nutrient intake and without indirect effects associated to their caloric value or other signaling pathways. Adult *RagA*^GTP^ mice also allow for the interrogation of the consequences of full-body constitutive mTORC1 activity after development, as other genetic tools of activation of mTORC1 in mice (such as loss of the TSC complex) result in developmental lethality^4^.

## Results

### Rescue of neonatal death of *RagA*^GTP^ mice

We have previously engineered a murine genetic system (*RagA*^GTP^ mice^15^) to drive constitutive nutrient signaling to mTORC1 in all organs. This tool allows for determining the consequences of chronic nutrient-dependent activation on mTORC1 activity in vivo, without elevating nutrient levels and the consequences that this elevation may have on other signaling pathways. Generating adult mice with constitutive RagA signaling was not straightforward because homozygous expression of the *RagA*^GTP^ allele in the mixed 75:25 C57BL/6:129 Sv background, in which the genetic system was engineered, albeit yielding expected Mendelian proportions up to birth, resulted in a fully-penetrant early postnatal lethality^15^. In contrast, *RagA*^GTP/+^ cells and mice had normal regulation of mTORC1 activity and normal phenotype^15,21^. We backcrossed the *RagA*^GTP^ strain into a pure C57BL/6 genetic background and found sub-Mendelian frequency of *RagA*^GTP/GTP^ neonates (Supplementary Fig. 1a). Moreover, and in contrast to our previous findings in the original 75:25 C57BL/6:129 Sv background, a large fraction of *RagA*^GTP/GTP^ pups showed deformities and/or were visibly swollen with features inconsistent with fetal viability (Supplementary Fig. 1a, b). Hence, we reasoned that enriching the *RagA*^GTP^ strain to the 129/Sv background could result in a milder phenotype that would allow partial neonatal survival. This rescue occurred, albeit marginally, as only 5% of *RagA*^GTP/GTP^ pups survived long enough to be weaned (3 weeks) (Supplementary Fig. 1c).

**Fig. 1:**
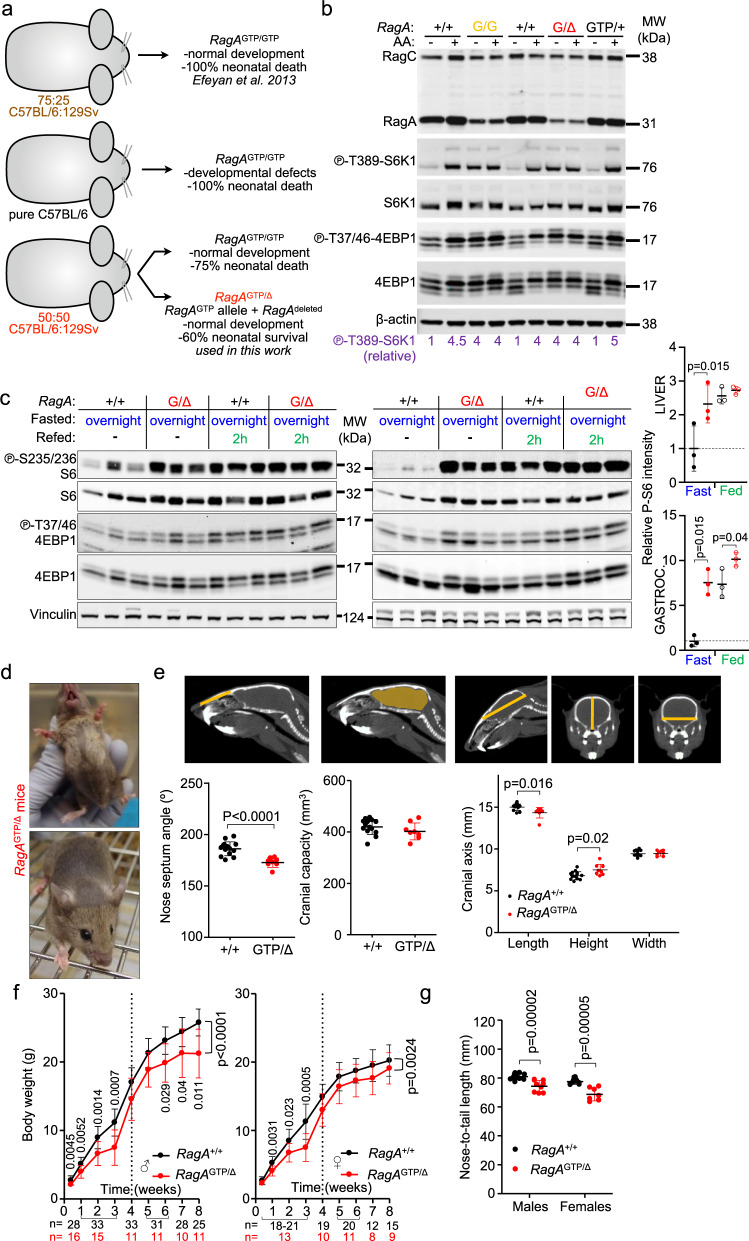
Consequences of constitutive nutrient signaling in adult mice. **a** Schematic summary of all genetic tools of expression of *RagA*^GTP^ and their outcomes. **b** Primary E13.5 MEFs derived from *RagA*^+/+^, *RagA*^GTP/GTP^, *RagA*^GTP/Δ^, and *RagA*^GTP/+^ embryos were deprived from all proteinogenic amino acids for 50 min and re-stimulated with amino acids for 10 min. Protein lysates were immunoblotted for the indicated proteins. The levels of P-T389 S6K1 were calculated relative to β-actin levels and normalized to the average level of the first *RagA*^+/+^ mice without AA (bottom, purple). The experiment was repeated at least twice with different samples. **c** 15- to 19-week-old *RagA*^+/+^ and *RagA*^GTP/Δ^ females were deprived from food for 16 h, followed by ad libitum refeeding for the last 2 h before euthanasia. Whole-cell protein lysates from the liver (left) and gastrocnemius muscle (right) were immunoblotted for the indicated proteins, with the quantification of P-S235/236-S6 intensity relative to vinculin, Fast: Fasted. (*n* = 3). Statistical significance was calculated by using 2-way ANOVA with Sidak’s multiple comparison correction. **d** Representative pictures of the hypopigmentation observed in *RagA*^GTP/Δ^ mice. **e** Craniofacial morphometric changes in the skull of *RagA*^GTP/Δ^ mice. Representative pictures of the measured parameters (top). Quantification of the indicated parameters (bottom). *RagA*^+/+^ (*n* = 14) and *RagA*^GTP/Δ^ (*n* = 8) mice (bottom). Statistical significance was calculated by using unpaired two-tailed t-test. For cranial axes was also corrected for multiple comparisons (Holm-Sidak). **f** Body weight of *RagA*^+/+^ (males *n* = 25-33, females *n* = 12-21) and *RagA*^GTP/Δ^ (males *n* = 10-16; females *n* = 8–13) at indicated times. Statistical significance was calculated by using 2-way ANOVA (mixed-effects model RELM) with Sidak’s multiple comparison correction. **g** Nose-to-tail length of *RagA*^+/+^ (males *n* = 15, females *n* = 10) and *RagA*^GTP/Δ^ (males *n* = 9; females *n* = 7) mice, measured with a caliper. Statistical significance was calculated by using unpaired two-tailed t-test corrected for multiple comparisons (Holm–Sidak). In all panels, horizontal lines indicate the mean and error bars depict Standard deviation (SD).

The co-expression of wild-type and mutant alleles of certain GTPases, such as the K-Ras GTPase, suppresses the signaling activation elicited by active mutant variant^22^, phenomenon likely to be at work in the *RagA*^GTP/+^ cells and mice, which showed no detectable increase in mTORC1 signaling^15,21^. Hence, we restricted the expression of *RagA*^GTP^ to one allele, in the absence of expression from the other allele, and indeed, survival of *hemizygous RagA*^GTP^ mice (*RagA*^GTP/null^, hereafter referred to as *RagA*^GTP/∆^) in a 50:50 mixed background was significantly improved, with 60% survival of the expected Mendelian ratio (Supplementary Fig. 1d; 15% survivors at weaning from an expected 25% mice). No obvious, macroscopic deformities could be appreciated in *RagA*^GTP/∆^ neonates, indicating an amelioration of the phenotype previously observed in pure C57BL/6 background. The differential outcomes of these genetic systems are summarized in Fig. 1a. The partial rescue of early lethality of *RagA*^GTP/Δ^ mice in 50:50 C57BL/6:129 Sv background occurred without noticeable normalization of mTORC1 activity in hemizygous *RagA*^GTP/∆^ cells when compared to *RagA*^GTP/GTP^ in primary mouse embryonic fibroblasts (MEFs), revealed by phosphorylation of the mTORC1 target S6K in threonine 389 and phosphorylation of 4EBP1 in threonines 37 and 46 (Fig. 1b). As reported, mTORC1 signaling in heterozygous *RagA*^GTP/+^ MEFs was indistinguishable from *RagA*^+/+^ counterparts (Fig. 1b), indicating that wild-type RagA, when present, suppresses the effect of a GTP-locked RagA.

### Characterization of mice with constitutive nutrient signaling

To determine to what extent was mTORC1 activity increased in adult *RagA*^GTP/Δ^ mice, we subjected mice to overnight fasting followed by either 30 min or 2 h refeeding and obtained whole protein extracts from several organs. Fig. 1c shows increased mTORC1 pathway activity in organs from *RagA*^GTP/∆^ mice during fasting periods, which was more noticeable in liver and gastrocnemius (Fig. 1c), and in kidney and heart (Supplementary Fig. 1e), but not as strong in white and brown adipose tissues (Supplementary Fig. 1f). Noteworthy, upon refeeding, the maximal phosphorylation of mTORC1 targets was similar in most organs from *RagA*^+/+^ and *RagA*^GTP/∆^ mice (Fig. 1c and Supplementary Fig. 1e, f), suggesting that genetic activation of the nutrient signaling pathway results in resistance to inhibition by fasting, but not in overall supra-physiological activation of the mTORC1 pathway. This absence of increased maximal mTORC1 activity was also seen in *RagA*^GTP/∆^ MEFs (Fig. 1b). Overall, the results of these signal transduction experiments show that *RagA*^GTP/Δ^ mice have increased, but not supra-physiological mTORC1 activity in the fasted state, consistently with a constitutively-active nutrient / Rag GTPase signaling input.

All weaned *RagA*^GTP/∆^ mice exhibited a fully-penetrant hypopigmented coat (Fig. 1d), a phenotype also observed in tuberous sclerosis patients and in other syndromes related with increased mTORC1 activity^23^, and also in *TSC1 floxed* strain bred to a strain of transgenic mice that express the Cre recombinase in a melanocyte-specific promoter^7^. Moreover, and although not appreciable at birth, visual examination of adult *RagA*^GTP/∆^ mice also revealed stereotypical craniofacial morphometric changes, which were confirmed and quantified by computerized axial tomography scans. Three morphometric alterations were most prominent: aberrant nasal septum angle (Fig. 1e, left), decreased length and increased height of the skull (Fig. 1e, right), all of which occurred without significant changes in the total cranial capacity (Fig. 1e, middle) and without obvious behavioral abnormalities. Consistently with the deformities observed in adult *RagA*^GTP/∆^ mice (Fig. 1e) and in *RagA*^GTP/GTP^ fetuses in pure C57BL/6 background (Supplementary Fig. 1a, b), skull and craniofacial deformities are also prevalent in patients with syndromes caused by mutations in components of the PI3K/mTORC1 pathway^24^.

Reduced body weight, as observed in neonates^15^, was maintained in young and old *RagA*^GTP/∆^ mice (Fig. 1f, and Supplementary Fig. 1g). This reduction correlated with a smaller nose-to-tail distance (Fig. 1g), and with smaller WAT and BAT depots (Supplementary Fig. 1h), also quantified as percentage of fat by means of densitometric analysis (Supplementary Fig. 1i). Decreased weight was not a consequence of abnormal food intake (Supplementary Fig. 1j) neither caused by increased voluntary movement in ad libitum conditions (Supplementary Fig. 1k).

### Compromised survival of adult *RagA*^GTP^ mice

**Fig. 2:**
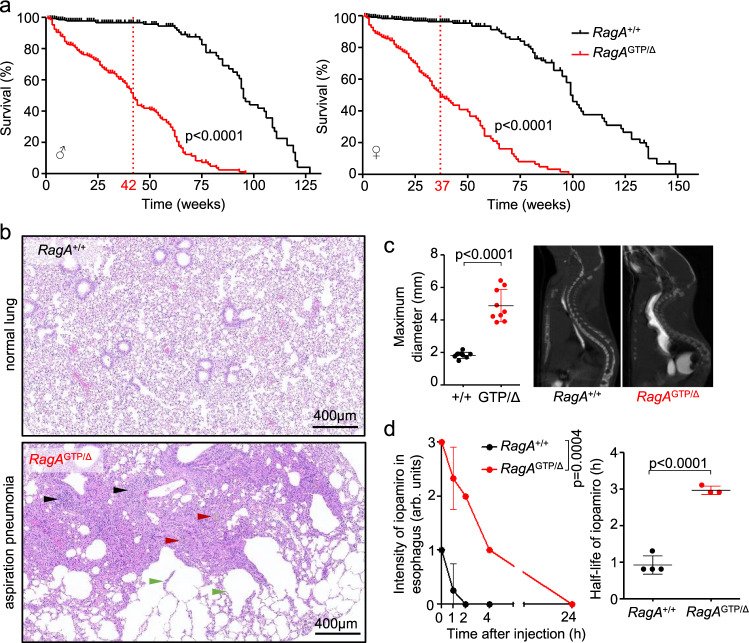
Premature death of *RagA*^GTP/∆^ mice. **a** Kaplan-Meier survival curves of *RagA*^+/+^ and *RagA*^GTP/Δ^ males (*n* = 503 and *n* = 235) and females (*n* = 521 and *n* = 220). Mean survival depicted in red. Statistical significance was calculated with the log-rank (Mantel-Cox) test. **b** Representative H&E staining of a normal lung (*RagA*^+/+^) and a lung with areas of pneumonia (*RagA*^GTP/Δ^), black arrowheads indicate infiltration of inflammatory cells, red arrowheads indicate the presence of macrophages with hemosiderin and green arrowheads indicate areas of emphysema. This phenotype was observed in at least 32 mutant mice. **c** (Left) Maximum esophageal diameter was measured upon sacrifice of *RagA*^+/+^ (*n* = 9) and *RagA*^GTP/Δ^ (*n* = 9) mice. (Right) Representative CT image of an esophagus administered with an oral contrast (iopamiro). Statistical significance was calculated by using unpaired two-tailed t-test. **d** Quantification of remnant iopamiro in the esophagus following its administration in *RagA*^+/+^ (*n* = 4) and *RagA*^GTP/Δ^ (*n* = 3) mice. Half-life of iopamiro was calculated from the slope of each individual curves and analyzed by using an unpaired two-tailed t-test. For the intensity of iopamiro, statistical significance was calculated by using 2-way ANOVA (mixed-effects model RELM). In all panels, horizontal lines indicate the mean and error bars depict Standard deviation (SD).

In spite of the partial rescue of neonatal lethality upon hemizygous expression of *RagA*^GTP^ in a 50:50 background, survival of *RagA*^GTP/∆^ mice was also compromised in adulthood, with a mean survival of *RagA*^GTP/∆^ males and females around 9 months (Fig. 2a and Supplementary Fig. 2a). Necropsies from *RagA*^GTP/∆^ mice, euthanized due to a sharp decline in body condition, frequently revealed aspiration pneumonia (Fig. 2b). While the cause of death could not be unequivocally ascertained in all mice, fatal aspiration pneumonia in a large fraction of adult *RagA*^GTP/∆^ mice was most likely caused by the presence of fully penetrant megaesophagus (Fig. 2c), itself a possible cause for aspiration pneumonia upon regurgitation, and ultimately caused by a distended esophagus. To formally determine the potential existence of a functional defect in esophageal transit, we monitored the oral-cavity-to-stomach transit, by means of oral administration of iopamiro, an imaging contrast reagent, which showed a partial delay of about 2 h in the clearing of the oral contrast in the esophagus of *RagA*^GTP/∆^ mice (Fig. 2d). Of note, this modest 2 h transit delay was unlikely to affect the kinetics of fasting – refeeding cycles and experiments, as a short-term drop in glycaemia upon 12 h fasting was indistinguishable from that of *RagA*^+/+^ mice (Supplementary Fig. 2b).

### Glucose homeostasis defects in *RagA*^GTP^ mice

**Fig. 3:**
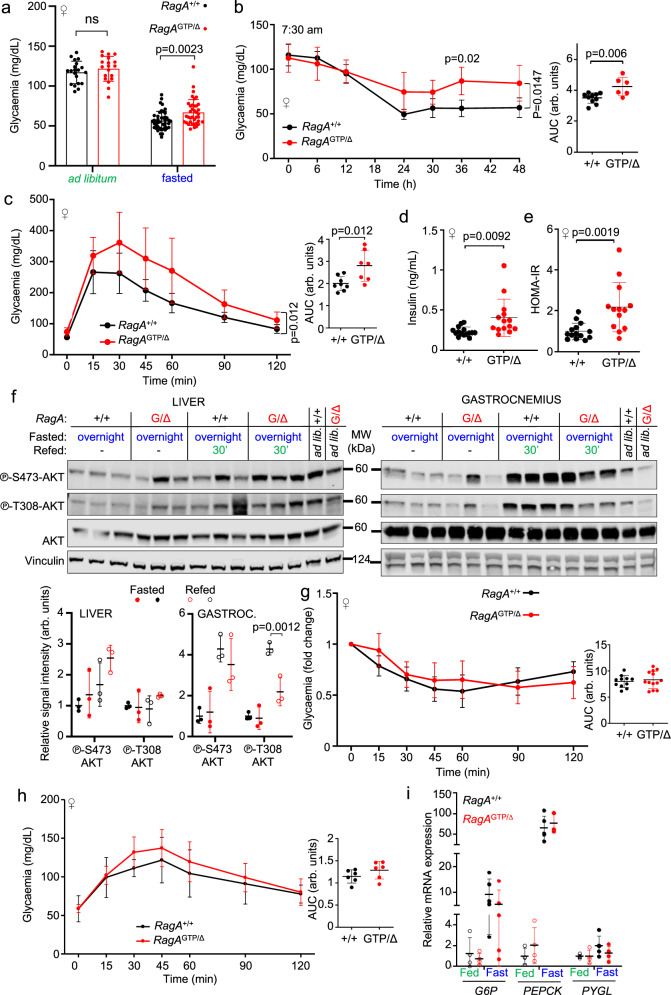
Constitutive activation of *RagA* compromises systemic glucose homeostasis. **a** Glycaemia of 5- to 23-week-old *RagA*^+/+^ and *RagA*^GTP/Δ^ females in ad libitum conditions and after 16 h of fasting. *RagA*^+/+^ (ad libitum *n* = 20 and fasted *n* = 47). *RagA*^GTP/Δ^ (ad libitum *n* = 20 and fasted *n* = 38). Statistical significance was calculated by using 2-way ANOVA with Sidak’s multiple comparison correction. **b** 24- to 33-week-old *RagA*^+/+^ (*n* = 10) and *RagA*^GTP/Δ^ (*n* = 6) females were starved for 48 h. Glucose was monitored every 6 h except in between 8 pm and 8 am. **c** Glucose tolerance test (GTT) of 14- to 22-week-old *RagA*^+/+^ (*n* = 8) and *RagA*^GTP/Δ^ (*n* = 7) females. **d** Insulin levels of 18- to 33-week-old *RagA*^+/+^ (*n* = 15) and *RagA*^GTP/Δ^ (*n* = 14) females fasted 16 h. **e** HOMA-IR ratio calculated from the ratio between insulin and glucose levels obtained from 18- to 33-week-old *RagA*^+/+^ (*n* = 15) and *RagA*^GTP/Δ^ (*n* = 14) females fasted for 16 h. For **d** and **e** statistical significance was calculated by using unpaired two-tailed t-test. **f** 25- to 36-week-old *RagA*^+/+^ and *RagA*^GTP/Δ^ females were deprived from food for 16 h and sacrificed following a 30 min refeeding. Ad libitum *(ad lib.)* fed mice were also included. Protein lysates from the liver and gastrocnemius muscle (Gastroc.) were immunoblotted for the indicated proteins (top) and the quantification of the intensity relative to vinculin  was calculated for the indicated antibodies (bottom). Statistical significance was calculated by using 2-way ANOVA with Sidak’s multiple comparison correction. **g** Insulin tolerance test (ITT) of 19- to 29-week-old *RagA*^+/+^ (*n* = 12) and *RagA*^GTP/Δ^ (*n* = 13) females. **h** Pyruvate tolerance test (PTT) of 20- to 22-week-old *RagA*^+/+^ and *RagA*^GTP/Δ^ (*n* = 6) females. **i** RT-qPCR of livers from 15- to 36-week-old *RagA*^+/+^ (fasted *n* = 5; fed *n* = 4) and *RagA*^GTP/Δ^ (fasted *n* = 5; fed *n* = 4) females. Expression levels of the indicated genes relative to those of β-actin were relativized to the average level of *RagA*^+/+^ mice in fed state. Fast: Fasted. For **b**, **c**, **g**, and **h**, statistical significance was calculated by using 2-way ANOVA with Sidak’s multiple comparison correction. The area under the curve (AUC) was calculated and statistical significance was calculated by using unpaired two-tailed t-test. In all panels, horizontal lines indicate the mean and error bars depict Standard deviation (SD).

To ascertain the consequences of constitutive nutrient signaling on the control of glucose homeostasis, compromised in other mouse models of deregulated mTORC1 activity^3^, we first measured glycaemia in *RagA*^+/+^ and *RagA*^GTP/Δ^ mice in ad libitum and fasted states. In contrast to the profound hypoglycaemia of *RagA*^GTP/GTP^ neonates^15^, adult *RagA*^GTP/Δ^ males and females underwent a mild, but significant hyperglycaemic state after 16 h fasting (Fig. 3a and Supplementary Fig. 3a). Under extended fasting (up to 48 h), *RagA*^GTP/Δ^ mice maintained significantly higher circulating glucose levels than *RagA*^+/+^ (Fig. 3b and Supplementary Fig. 3b). Moreover, *RagA*^GTP/Δ^ female mice were moderately glucose intolerant (Fig. 3c and Supplementary Fig. 3c) and had increased fasted insulin levels and the HOMA-IR ratio, indicative of systemically decreased insulin sensitivity (Fig. 3d, e and Supplementary Fig. 3d, e). Because mTORC1 activity unleashes a negative feedback loop that indirectly suppresses the activation of PI3K-Akt that can impair the positive effect of insulin in glucose uptake^25–28^, we ascertained the phosphorylation status of Akt and some Akt targets. Interestingly, the livers of *RagA*^GTP/Δ^ mice did not show evidence of suppressed Akt signaling (Fig. 3f and Supplementary Fig. 3f), but the gastrocnemius muscle presented a slight, but significant decrease in the phosphorylated Akt, and in some of its targets, upon refeeding (Fig. 3f and Supplementary Fig. 3g). This differential effect in liver and skeletal muscle highlights the importance of this negative feedback loop for muscle-regulated physiology and in the onset of insulin resistance^29^, but is unlikely to fully explain the mild systemic glucose intolerance^30^. Despite this mild decrease in Akt signaling, an insulin tolerance test (ITT) revealed no alterations in insulin-induced decrease in glycaemia in *RagA*^GTP/Δ^ mice (Fig. 3g and Supplementary Fig. 3h), and ex vivo glucose uptake measured in isolated extensor digitorum longus (EDL) and in soleus muscles derived from *RagA*^GTP/Δ^ and wild-type mice was similar between genotypes (Supplementary Fig. 3i). Thus, the intolerance to glucose, and significant increase in fasting insulin levels of female *RagA*^GTP/Δ^ mice do not appear to stem from an intrinsic impairment in glucose uptake. Because increased gluconeogenesis can lead to increased fasting hyperglycaemia, we conducted pyruvate tolerance test (PTT), and observed that pyruvate injection caused a similar increase in glycaemia in *RagA*^+/+^ and *RagA*^GTP/Δ^ mice (Fig. 3h and Supplementary Fig. 3j). In addition, and consistently with no differences in hepatic Akt signaling, *RagA*^GTP/Δ^ livers showed normal levels of mRNAs encoding key enzymes for hepatic glucose production (Fig. 3i). Finally, glycogen levels of skeletal muscle and liver were similar in *RagA*^+/+^ and *RagA*^GTP/Δ^ in fed conditions and equally depleted during fasting (Supplementary Fig. 3k, l).

Altogether, these results suggest that constitutive nutrient signaling to mTORC1, in the absence of increased nutrient intake, moderately compromises glucose homeostasis. This result provides one potential explanation for the predictive value of elevated branched-chain amino acids for type 2 diabetes in otherwise healthy individuals^31^.

### Proteomics analysis of livers from *RagA*^GTP^ mice

**Fig. 4:**
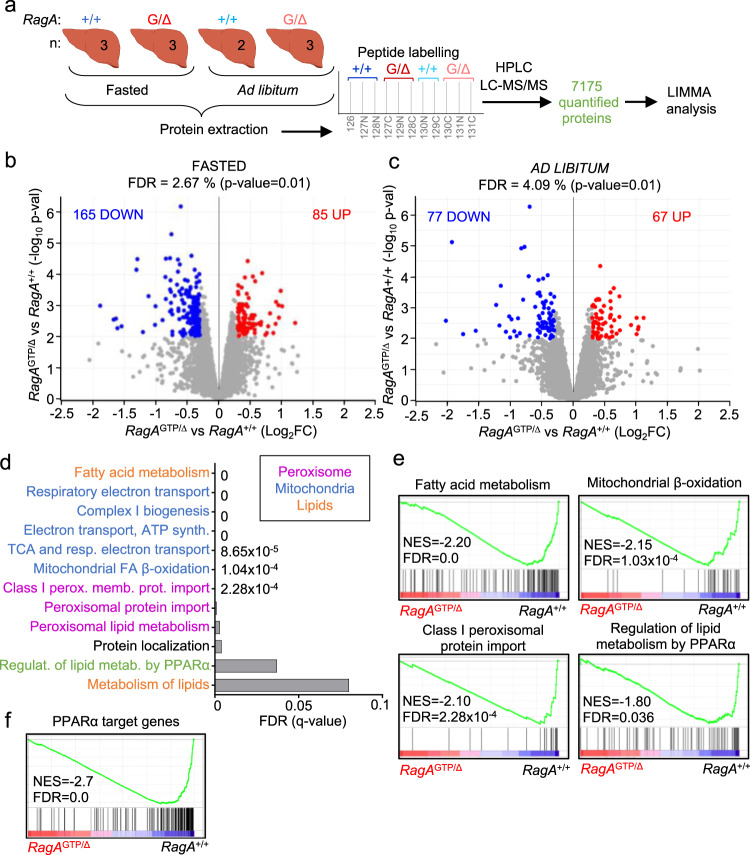
Proteomic analysis of *RagA*^GTP/Δ^ livers revealed blunt PPARα transcriptional program during fasting. **a** Schematic summary of the proteomics experimental setting. Protein lysates from livers of 19- to 24-week-old *RagA*^+/+^ (ad libitum *n* = 2 and fasted *n* = 3) and *RagA*^GTP/Δ^ (ad libitum *n* = 3 and fasted *n* = 3) females were extracted, processed, and labelled as indicated. **b** Volcano plot highlighting significantly different protein levels in livers from fasted *RagA*^GTP/Δ^ (*n* = 3) vs. fasted *RagA*^+/+^ (*n* = 3) mice. **c** Volcano plot highlighting significantly different protein levels in livers from ad libitum fed *RagA*^GTP/Δ^ (*n* = 3), vs. ad libitum fed *RagA*^+/+^ (*n* = 2) mice. Statistical significance for sections **b** and **c** was calculated using the LIMMA program as indicated in Materials and Methods. **d** Graphical representation of the false discover rates (FDRs) from the indicated Reactome gene sets. **e**, **f** Enrichment of gene sets (GSEA) related to indicated Reactome signatures (**e**) or related to our PPARα targets curated list (**f**) in livers from fasted *RagA*^GTP/Δ^ (*n* = 3) and *RagA*^+/+^ (*n* = 3) mice. NES: normalized enrichment score; FDR: false discovery rate and error bars depict Standard deviation (SD).

To identify cellular changes driven by constitutive nutrient signaling in vivo in an unbiased manner, we conducted whole-proteome analysis of livers obtained from overnight fasted and ad libitum fed wild-type and *RagA*^GTP/∆^ mice (Fig. 4a). From a total of 7175 proteins quantified in all the samples, we observed that the levels of 250 proteins were significantly different in *RagA*^GTP/∆^ versus *RagA*^+/+^ livers from mice fasted overnight (Fig. 4b, Supplementary Data. 1), and 144 were significantly different in *RagA*^GTP/∆^ versus *RagA*^+/+^ livers in ad libitum conditions (Fig. 4c). Only 92 proteins were found significantly altered when comparing ad libitum vs. fasted conditions in *RagA*^GTP/∆^ livers (Supplementary Fig. 4a), almost half of the observed changes in *RagA*^+/+^ mice (Supplementary Fig. 4b), indicating higher similarity between the two nutritional states when mTORC1 is constitutively active. Principal Component Analysis (PCA) scattered *RagA*^+/+^ and *RagA*^GTP/∆^ samples in identifiable, different regions (Supplementary Fig. 4c). Interestingly, a significant fraction of the differentially abundant proteins in *RagA*^GTP/∆^ versus *RagA*^+/+^ were either decreased or increased in both fasted and fed states (Supplementary Fig. 4d; *p* < 6.028 × 10^−54^ for upregulated, and *p* < 1.119 × 10^−30^ for downregulated proteins), indicating that certain changes caused by constitutive nutrient signaling do not depend on the nutritional status.

We next analyzed the differential proteome of *RagA*^+/+^ versus *RagA*^GTP/Δ^ samples in fasting conditions, as this comparison would presumably show the impact of elevated nutrient signaling in the physiological setting in which it is normally suppressed. Consistently with the expected changes caused by genetic activation of Rag GTPase signaling, gene set enrichment analysis (GSEA)^32^ showed the expected enrichment of a mTOR signaling signature in *RagA*^GTP/Δ^ livers in fasted state (Supplementary Fig. 4e), but not in ad libitum fed *RagA*^+/+^ versus *RagA*^GTP/Δ^ mice. The proteomic dataset also showed significant enrichment of proteins encoded by classical TOP mRNAs (Supplementary Table 1) in *RagA*^GTP/Δ^ samples from fasted mice (Supplementary Fig. 4f). This association was expected because mTORC1 promotes global protein synthesis by driving the translation initiation of 5′ TOP mRNAs that encode most of the translation machinery, including ribosomal proteins and other known translation initiators^33,34^. Surprisingly, targets of TFEB^35,36^, a transcription factor inhibited by direct phosphorylation by mTORC1, were not down-regulated in *RagA*^GTP/Δ^ samples (Supplementary Fig. 4g). This result suggests that, whereas activation of RagC strongly suppresses TFEB activity^37^, the effect of genetic activation of RagA alone may have a more subtle effect on such inhibition.

Only three main functional categories stood out among the signatures significantly depleted from *RagA*^GTP/Δ^ livers under fasting conditions: lipid metabolism, mitochondria, and peroxisomes (Fig. 4d, e and Supplementary Fig. 4h). These three categories are largely composed by proteins encoded by transcriptional target genes of peroxisome proliferator-activated receptor alpha (PPARα). Indeed, signatures of PPARα targets, including one list of 168 PPARα target genes curated by us (Supplementary Table 1), were also significantly depleted (Fig. 4f) from *RagA*^GTP/Δ^ vs. *RagA*^+/+^ livers obtained from fasted mice (Fig. 4d–f, and Supplementary Fig. 4h). These data suggest that a major consequence of the inhibition of nutrient signaling during fasting is the induction of PPARα targets.

### Constitutive nutrient signaling impairs the PPARα program

**Fig. 5:**
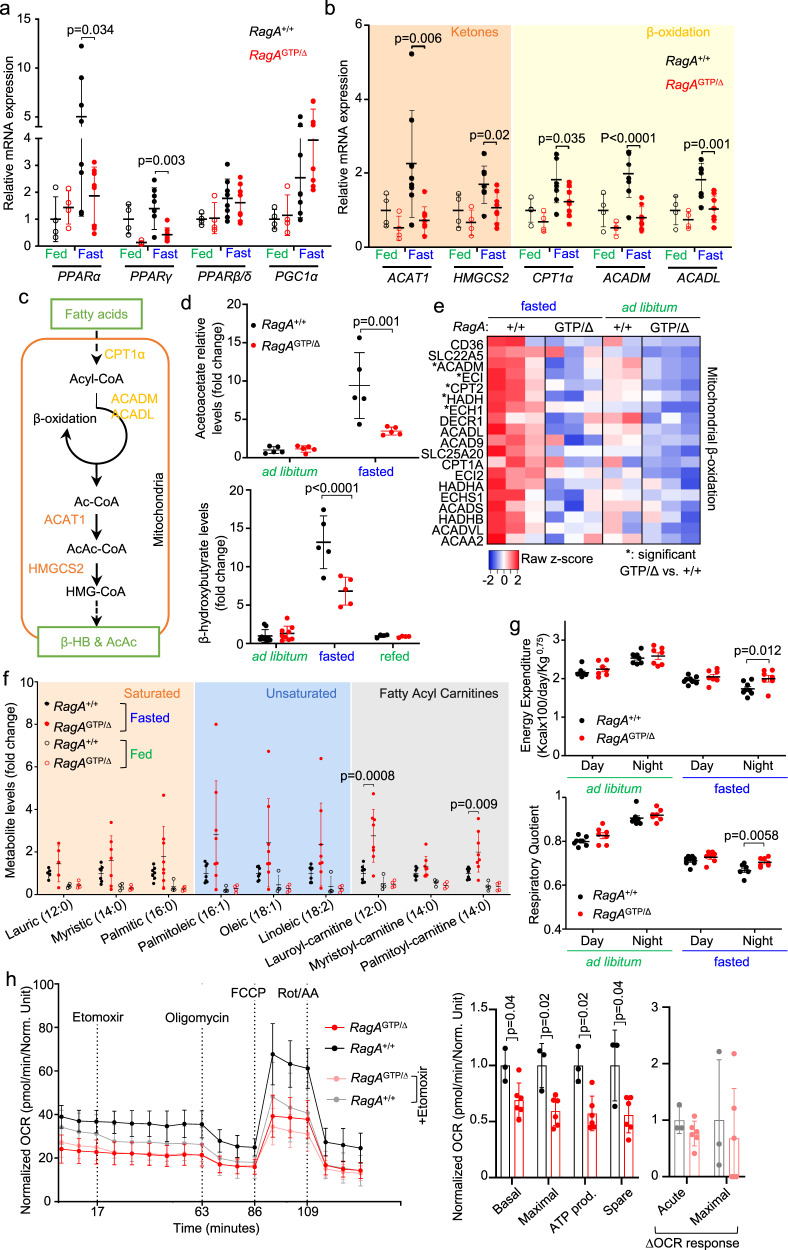
Impaired hepatic ketogenesis and mitochondrial β-oxidation in *RagA*^GTP/∆^ mice. **a**, **b** RT-qPCR of livers from 15- to 36-week-old *RagA*^+/+^ (fasted *n* = 8; fed *n* = 4) and *RagA*^GTP/Δ^ (fasted *n* = 8; fed *n* = 4) females. Expression levels of the indicated genes relative to the average level in fed *RagA*^+/+^ mice. β-actin was used as housekeeping gene. Fast: Fasted. **c** Schematic representation of the key steps of β-oxidation and ketogenesis. Enzymes measured by RT-PCR involved in ketogenesis (orange) and β-oxidation (yellow). β-HB: β-hydroxybutyrate; AcAc: Acetoacetate. **d** 27- to 35-week-old *RagA*^+/+^ (*n* = 5) and *RagA*^GTP/Δ^ (*n* = 5) females were fasted for 24 h and acetoacetate levels before and after fasting were quantified from serum by targeted LC‐MS. For β-hydroxybutyrate levels, 15- to 19-week-old *RagA*^+/+^ and *RagA*^GTP/Δ^ females were fasted for 16 h, followed by a 2 h refeeding period. Mice were bled before the fasting period and euthanized. β-hydroxybutyrate levels were analyzed by targeted LC‐MS. Data are relative to the levels of ad libitum *fed RagA*^+/+^. *RagA*^+/+^ and *RagA*^GTP/Δ^ mice (ad libitum fed *n* = 9; fasted *n* = 5; refed *n* = 4). **e** Heat map from all proteins involved in mitochondrial β-oxidation detected in the proteomics experiment from Fig. 4. *RagA*^+/+^ (fasted *n* = 3; ad libitum fed *n* = 2) and *RagA*^GTP/Δ^ (fasted *n* = 3; ad libitum fed *n* = 3). Statistical significance was calculated using the LIMMA program as indicated in Materials and Methods. The * indicates significant difference between *RagA*^+/+^ and *RagA*^GTP/∆^ in fasted conditions. **f** 15- to 19-week-old *RagA*^+/+^ and *RagA*^GTP/∆^ females were fasted for 16 h followed by a 2 h ad libitum refeeding period (fed state). Liver was collected upon the sacrifice and the levels of indicated metabolites were quantified by targeted LC‐MS. Data are shown relative to the levels of samples from fasted *RagA*^+/+^. *RagA*^+/+^ and *RagA*^GTP/∆^ (fasted *n* = 8; fed *n* = 4). **g** Quantification of Energy Expenditure (EE) and Respiratory Quotient (RQ) of 15- to 19-week-old *RagA*^+/+^ (*n* = 8) and *RagA*^GTP/Δ^ (*n* = 7) females. Mice were monitored in metabolic cages for 3 days and nights in ad libitum conditions and for 27 h in fasting. The average value of 3 days and 3 nights in ad libitum conditions and 1 day and night for fasted conditions is shown. **h** Normalized Oxygen Consumption Rate (OCR) in primary hepatocytes from *RagA*^+/+^ (*n* = 3) and *RagA*^GTP/Δ^ (*n* = 6) mice after the addition of Etomoxir (40 µM), Oligomycin (1.5 µM), FCCP (0.3 µM) and Rotenone/antimycin A (Rot/AA) (0.5 µM) (left). Quantification of indicated parameters (right) relative to the average OCR of *RagA*^+/+^ hepatocytes. For the quantification of the acute and maximal ∆OCR, basal and maximal respiration respectively were compared before and after etomoxir treatment. Statistical significance was calculated by using unpaired two-tailed t-test corrected for multiple comparisons (Holm-Sidak). For **a**, **b**, **d**, **f**, and **g** statistical significance was calculated by using 2-way ANOVA with Sidak’s multiple comparison correction. In all panels, horizontal lines indicate the mean and error bars depict Standard deviation (SD).

The PPAR family of nuclear receptors is composed of tree isoforms (α, β/δ, and γ) that differ in tissue distribution, ligand specificity, and metabolic regulatory activities^38^. mTOR controls PPARα, PPARγ, and transcriptional co-activators such as PGC1α^39,40^. Hence, we evaluated the transcriptional levels of *PPAR* family members in *RagA*^+/+^ and *RagA*^GTP/Δ^ livers. In agreement with our proteomics analysis, the mRNA levels of *PPARα* were significantly decreased upon fasting in *RagA*^GTP/Δ^ mice. Interestingly, *PPARγ* levels were also decreased, while no changes were noticeable in the levels of *PPARβ/δ* and *PGC1α* (Fig. 5a). Because mTORC1 activity, together with mTORC1-independent actions of Akt, promotes a SREBF- and PPARγ-dependent lipogenic program, we measured the mRNA levels of the transcription factor *Srebf* and of some key lipogenic factors, such as *Acaca, Acly*, and *Fasn*, but found no experimental support for increased expression of these factors in livers from *RagA*^GTP/Δ^ mice (Supplementary Fig. 5a) neither were they increased in the proteomics datasets (Supplementary Fig. 5b). In addition, the hepatic triglyceride content was not increased in *RagA*^+/+^ and *RagA*^GTP/Δ^ mice (Supplementary Fig. 5c), consistently with other models of increased hepatic mTORC1 activity^41^. These results are consistent with the strict requirement for increased Akt activation, in addition to constitutive nutrient signaling - mTORC1 activity, to drive lipogenesis in the liver. Interestingly, and in contrast to the findings in liver, expression of *PPARγ* was increased in subcutaneous WAT from *RagA*^GTP/Δ^ in the fed state (Supplementary Fig. 5d), suggesting that the impact of nutrient signaling activation on the transcriptional program of lipogenesis is not identical in the liver and in WAT. In subcutaneous WAT from *RagA*^GTP/Δ^ mice, neither the mRNA levels of *PPARα* nor its transcriptional targets involved in ketogenesis, β-oxidation or peroxisomal function were significantly decreased during fasting, as compared to R*agA*^+/+^ samples (Supplementary Fig. 5d). A similar picture was observed in skeletal muscle (Supplementary Fig. 5e). These results suggest that the PPARα program in the liver is more sensitive to constitutive nutrient signaling, compared to that of WAT and skeletal muscle.

A key role of PPARα is to promote a transcriptional program that elicits hepatic ketogenesis and fatty acid oxidation during fasting^42^. The mRNA levels of PPARα targets important for ketogenesis were decreased in livers from fasted *RagA*^GTP/Δ^ mice (Fig. 5b, c). Accordingly, fasting-induced ketogenesis was impaired in *RagA*^GTP/Δ^ mice (Fig. 5d), as revealed by the decrease in the levels of the ketone bodies acetoacetate and β-hydroxybutyrate, a defect that has been previously observed in liver-specific *TSC1* knock-out mice^40^. Additionally, mRNA levels of key enzymes involved in mitochondrial β-oxidation were also decreased in the livers from fasted *RagA*^GTP/∆^ mice as compared to *RagA*^+/+^ counterparts (Fig. 5b), differences that were also noticeable at the protein level (Fig. 5e and Supplementary Fig. 5f), pointing to a defect in lipid metabolism. An incomplete burning of lipids species can generate hepatic accumulation of lipid intermediates^43,44^, a trend we observed for medium and long saturated and unsaturated fatty acids and acyl-carnitine species in liver extracts from fasted *RagA*^GTP/Δ^ versus *RagA*^+/+^ mice (Fig. 5f). Interestingly, the accumulation of fatty acids in *RagA*^GTP/Δ^ livers occurred without concomitant changes in the levels of the same fatty derivatives and of free fatty acids in circulation (Supplementary Fig. 5g, h).

To ascertain whether the proteomic, transcriptomic, and metabolomic findings translated into a physiological metabolic defect, we conducted indirect calorimetric analyses in ad libitum fed and fasted *RagA*^GTP/Δ^ and *RagA*^+/+^ mice. *RagA*^GTP/Δ^ mice had no alterations in energy expenditure (EE) nor in the respiratory quotient (RQ) in ad libitum conditions, but interestingly, presented an increase in both EE and RQ upon fasting (Fig. 5g and Supplementary Fig. 5i), supporting a defective systemic adaptation to fasting. In agreement with an impaired fed-to-fasted adaptation, bodyweight loss was significantly higher in fasted *RagA*^GTP/Δ^ mice than in *RagA*^+/+^ (Supplementary Fig. 5j). Finally, the upward shift in RQ under fasting conditions is consistent with increased metabolism of carbohydrates to detriment fat/ketone body metabolism, as the biochemical observations suggested. Collectively, *RagA*^GTP/∆^ mice show defects in energy balance that manifest only under a fasted state, driven by an impairment in decreasing mTORC1 activity in a Rag GTPase-dependent manner upon nutrient limitation.

To evaluate whether the hepatocytes of *RagA*^GTP/∆^ mice had an intrinsic defect in mitochondrial activity, we measured ex vivo cellular respiration in primary isolated hepatocytes. Indeed, *RagA*^GTP/∆^ hepatocytes showed a striking reduction in basal and maximal respiration (Fig. 5h), while the suppression of fatty acid oxidation (FAO) by etomoxir was subtle in hepatocytes from both genotypes (Fig. 5h).

**Fig. 6:**
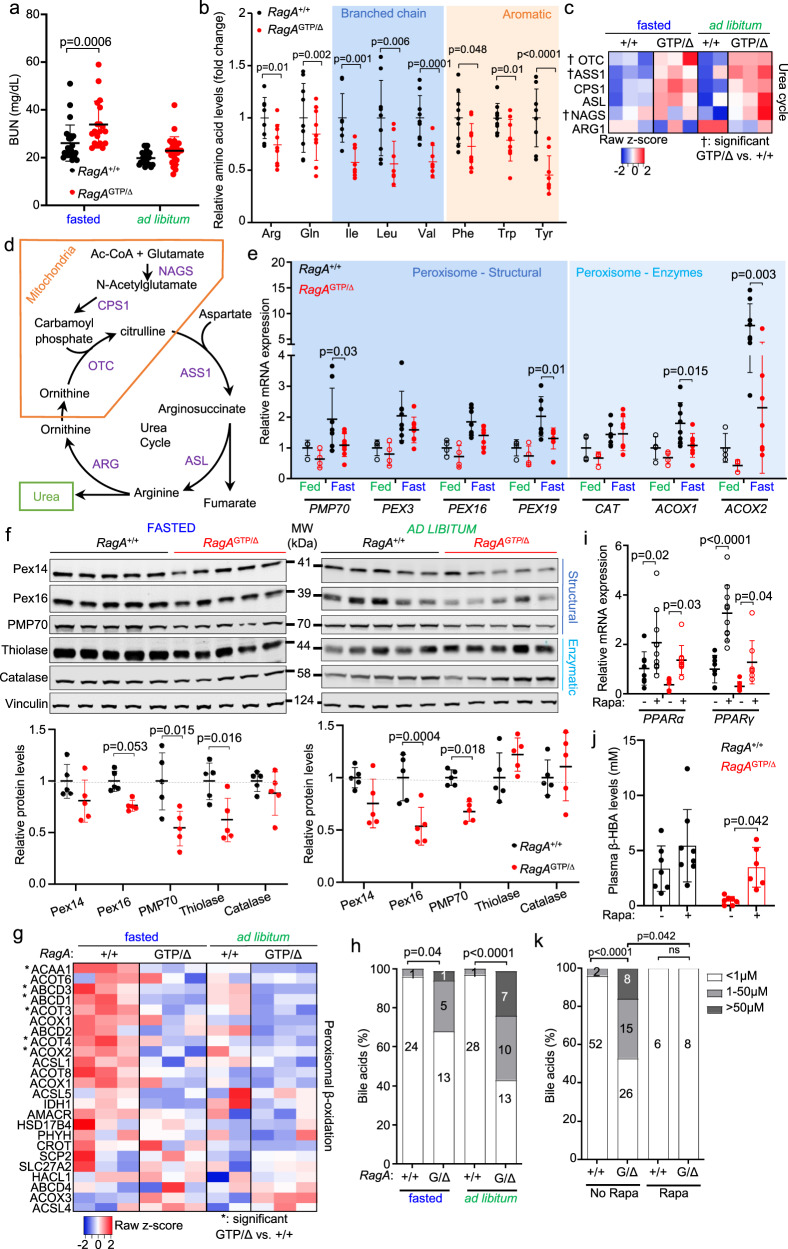
*RagA*^GTP/∆^ mice show increased amino acid catabolism and defective peroxisomes. **a** Blood Urea Nitrogen (BUN) was measured in 6- to 23-week-old *RagA*^+/+^ (fasted *n* = 25; ad libitum *n* = 19) and *RagA*^GTP/∆^ (fasted *n* = 19; ad libitum *n* = 20) males and females. **b** Amino acid levels were measured by targeted LC–MS in serum from 15- to 19-week-old ad libitum *RagA*^+/+^ (*n* = 9) and *RagA*^GTP/∆^ (*n* = 9) females. **c** Heat map from all proteins involved in Urea cycle detected in the proteomics experiment from Fig. 4. Statistical significance was calculated using the LIMMA program as indicated in Materials and Methods. The † indicates a significant difference between *RagA*^+/+^ and *RagA*^GTP/∆^ in ad libitum fed conditions. **d** Schematic representation of the Urea Cycle reactions, metabolites, and key enzymes. **e** RT-qPCR of livers from 15- to 36-week-old *RagA*^+/+^ (fasted *n* = 8; fed *n* = 4) and *RagA*^GTP/Δ^ (fasted *n* = 8; fed *n* = 4) females. Expression levels of the indicated genes relative to the average level in fed *RagA*^+/+^ mice. β-actin was used as housekeeping gene. **f** Livers were obtained from 18- to 28-week-old *RagA*^+/+^ and *RagA*^GTP/∆^ mice in ad libitum fed conditions or deprived from food for 16 h. Whole-cell protein lysates from liver were immunoblotted for the indicated proteins. For each protein, the levels in each lane are relative to vinculin levels and presented normalized to the average level of fasted *RagA*^+/+^ mice (n = 5). Gels/blots were processed in parallel. **g** Heat map from all proteins involved in peroxisomal β-oxidation detected in the proteomics experiment from Fig. 4. *RagA*^+/+^ (fasted *n* = 3; ad libitum fed n = 2) and *RagA*^GTP/∆^ (fasted *n* = 3; ad libitum fed *n* = 3). Statistical significance was calculated using the LIMMA program as indicated in Materials and Methods. The * indicates a significant difference between *RagA*^+/+^ and *RagA*^GTP/∆^ in fasted conditions. **h** Bile acid levels were measured in blood from 6- to 23-week-old *RagA*^+/+^ (fasted *n* = 25; ad libitum *n* = 29) and *RagA*^GTP/∆^ (fasted n = 19; ad libitum *n* = 30) males and females. **i** 11- to 21-week-old *RagA*^+/+^ and *RagA*^GTP/∆^ females were treated with rapamycin encapsulated in food for 2 weeks and fasted 16 h before sacrifice. mRNA levels of the indicated genes from liver extracts were quantified by RT-qPCR, relative to β-actin levels, and then normalized to the average levels of samples from non-treated *RagA*^+/+^ mice. *RagA*^+/+^ (no rapa *n* = 8; rapa *n* = 10) and *RagA*^GTP/∆^ (no rapa *n* = 8 rapa *n* = 9). **j** Mice treated or not treated with Rapamycin for 1 week were bleed after 16 h of fasting, then the levels of β-hydroxybutyrate (β-HBA) were addressed. 7- to 36-week-old *RagA*^+/+^ (no rapa *n* = 7; rapa n = 8) and *RagA*^GTP/∆^ (no rapa *n* = 7 rapa *n* = 6). **k** Bile acid levels were measured from blood samples from 6- to 23-week-old *RagA*^+/+^ (no rapa *n* = 53 and rapa *n* = 6) and *RagA*^GTP/∆^ (no rapa *n* = 49 and rapa *n* = 8). For **a**, **b**, **e**, **f**, **i**, and **j** statistical significance was calculated for individual metabolites by using 2-way ANOVA with Sidak’s multiple comparison correction. For **h** and **k** statistical significance was calculated by using Chi-square test. In all panels, horizontal lines indicate the mean and error bars depict Standard deviation (SD).

PPARα also regulates amino acid degradation and controls the expression of peroxisomal proteins, including those involved in peroxisomal β-oxidation^42^. Like *PPARα*^−/−^ mice, *RagA*^GTP/∆^ mice showed elevated plasma urea levels upon fasting (Fig. 6a) and a striking reduction in the circulating levels of several amino acids (Fig. 6b and Supplementary Fig. 6a). These changes occurred with a similar increase in protein and mRNA levels of hepatic enzymes involved in the urea cycle (Fig. 6c, d, and Supplementary Fig. 6b, c), which are transcriptionally repressed by PPARα^45,46^. Moreover, and in addition to the decrease in peroxin proteins detected by mass spectrometry (Supplementary Fig. 6d), mRNA levels of several structural peroxisomal genes and proteins detected by western blot were significantly decreased in livers of *RagA*^GTP/∆^ mice (Fig. 6e, f), strongly suggesting a consistent decrease in peroxisomes. Peroxisomes are involved in several physiologically relevant metabolic reactions, including long chain fatty acid oxidation and bile acid synthesis. Interestingly, both protein and mRNA levels of specific enzymes and transporters involved in peroxisomal β-oxidation were diminished in the livers of *RagA*^GTP/∆^ mice (Fig. 6e–g and Supplementary Fig. 6e). This decrease was also observed in primary hepatocytes isolated from *RagA*^GTP/∆^ mice, as compared to those from *RagA*^+/+^ mice (Supplementary Fig. 6f). Importantly, the levels of bile acids, controlled by peroxisomal activity, were abnormally high in sera from *RagA*^GTP/∆^ mice (Fig. 6h). Altogether, these defects underlie a complete, global impairment in the PPARα-dependent fasting metabolic program in mice with constitutive nutrient signaling.

### Reversion of metabolic defects by acute rapamycin treatment

Next, we tested whether impaired PPARα transactivation and its physiological consequences observed in *RagA*^GTP/∆^ mice could be reverted by short-time inhibition of mTORC1 with rapamycin. Administration of rapamycin partially reverted the impaired transcription of *PPARα* and *PPARγ* in *RagA*^GTP/∆^ mice, and it also increased their levels in *RagA*^+/+^ mice (Fig. 6i). Rapamycin also restored the expression of PPARα target genes involved in ketogenesis in livers from *RagA*^GTP/∆^ mice (Supplementary Fig. 6g) and fasting-induced ketogenesis (Fig. 6j). Although the expression of the PPARα target genes *Cpt1α, Acadm and Acadl*, involved in β-oxidation, was minimally affected by rapamycin (Supplementary Fig. 6g), the abnormally high levels of fatty-acid intermediates in the liver of *RagA*^GTP/∆^ mice were reduced (Supplementary Fig. 6h). Similarly, acute pharmacological inhibition of mTORC1 corrected the differences in peroxin protein levels (Supplementary Fig. 6i, j). Finally, the aberrantly high levels of circulating bile acids in *RagA*^GTP/∆^ mice were normalized by rapamycin (Fig. 6k). Hence, acute correction of mTORC1 signaling under fasting is efficacious to boost the defective PPARα activity caused by constitutive nutrient signaling.

### Metabolic phenotypes in liver-specific *RagA*^GTP^ mice

**Fig. 7:**
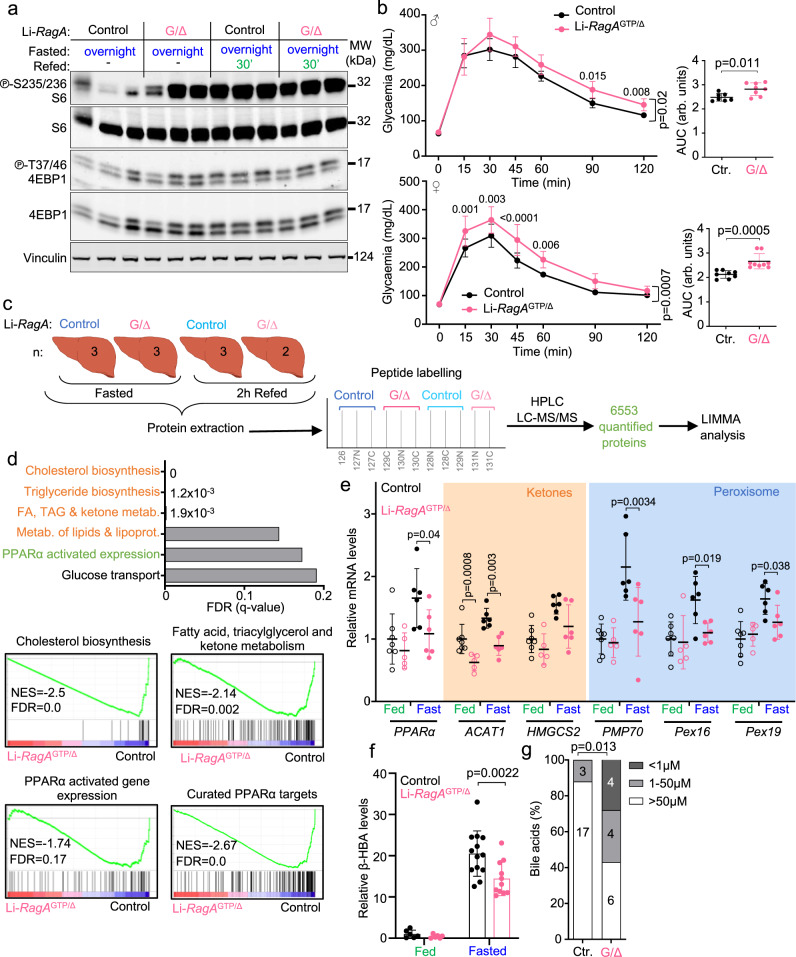
Li-*RagA*^GTP/∆^ mice partially recapitulate the metabolic defects of *RagA*^GTP/∆^ mice. **a** Control and Li-*RagA*^GTP/Δ^ males were deprived from food for 16 h and sacrificed following a 30 min refeeding. Protein lysates from the liver were immunoblotted for the indicated proteins. This experiment was repeated at least two more times with different samples obtaining similar results. **b** Glucose tolerance test of 7- to 12-week-old control (males *n* = 7 and females *n* = 8) and Li-*RagA*^GTP/Δ^ (males *n* = 8 and females *n* = 9) mice. Statistical significance was calculated by using 2-way ANOVA with Sidak’s multiple comparison correction. The area under the curve (AUC) was done and statistical significance was calculated by using unpaired two-tailed t-test. Ctr.: Control. **c** Schematic summary of the proteomics experimental setting. Protein lysates from livers of 23- to 42-week-old control (fed *n* = 3 and fasted *n* = 3) and Li-*RagA*^GTP/Δ^ (fed *n* = 2 and fasted *n* = 3) females were extracted, processed, and labelled as indicated. **d** Graphical representation of the false discover rates (FDRs) from the indicated Reactome gene sets (top). Enrichment of gene sets (GSEA) related to indicated Reactome signatures (bottom-left). Enrichment of gene sets (GSEA) related to our curated list of PPARα target genes (bottom-right) in livers from fasted *RagA*^GTP/Δ^ (*n* = 3) vs. *RagA*^+/+^ (*n* = 3) mice. NES: normalized enrichment score; FDR: false discovery rate. **e** RT-qPCR of livers from 23- to 42-week-old control (fasted *n* = 6; fed *n* = 7) and *RagA*^GTP/Δ^ (fasted *n* = 6; fed *n* = 5) females. Expression levels of the indicated genes relative to the average level in fed control mice. β-actin was used as housekeeping gene. **f** 12- to 27-week-old males and females in ad libitum (fed) or fasted for 24 h were bleed and the levels of β-hydroxybutyrate (β-ΗΒΑ) were quantified using NMR. Control (fed *n* = 6 and fasted *n* = 13) and Li-*RagA*^GTP/∆^ (fed *n* = 6 and fasted *n* = 11). Statistical significance was calculated by using 2-way ANOVA with Sidak’s multiple comparison correction. **g** Bile acid levels were measured from blood samples from 5- to 14-week-old ad libitum control (*n* = 20) and Li-*RagA*^GTP/∆^ (*n* = 14) males and females. Ctr.: Control. Statistical significance was calculated by using Chi-square test. In all panels, horizontal lines indicate the mean and error bars depict Standard deviation (SD).

To assess whether Rag GTPase signaling in the liver was responsible for the metabolic phenotypes observed in full-body *RagA*^GTP^ mice, we generated mice with constitutive activation of RagA restricted to hepatocytes (*RagA*^GTP/floxed^; Albumin-Cre^Tg^ mice, hereafter referred to as Li-*RagA*^GTP/Δ^ mice) (Supplementary Fig. 7a). Li-*RagA*^GTP/Δ^ mice were born at expected mendelian ratios without abnormalities (Supplementary Fig. 7b). As expected, livers from Li-*RagA*^GTP/Δ^ mice showed increased mTORC1 activity upon fasting (Fig. 7a), but not other organs, such as gastrocnemius muscle (Supplementary Fig. 7c). Unlike full-body *RagA*^GTP/Δ^ mice, Li-*RagA*^GTP/Δ^ mice had no differences in body weight (Supplementary Fig. 7d), nor in the percentage of fat (Supplementary Fig. 7e).

In contrast to full-body *RagA*^GTP/Δ^ mice, Li-*RagA*^GTP/∆^ mice had normal glycaemia during long-term fasting (Supplementary Fig. 7f). However, like full-body *RagA*^GTP/Δ^ mice, both male and female Li-*RagA*^GTP/Δ^ mice exhibit a mild but reproducible decrease in glucose tolerance (Fig. 7b). This alteration in glycaemia occurred without a concomitant increase in fasting insulin levels nor in the HOMA-IR ratio (Supplementary Fig. 7g). Indeed, Li-*RagA*^GTP/∆^ mice responded as wild-type counterparts when subjected to an ITT (Supplementary Fig. 7h). In agreement with the signaling results of livers from full-body *RagA*^GTP/Δ^ mice (Fig. 3f and Supplementary Fig. 3f), Akt signaling in vivo was normal in livers from Li-*RagA*^GTP/∆^ mice (Supplementary Fig. 7i), and was only mildly decreased in the gastrocnemius muscle of Li-*RagA*^GTP/∆^ mice (Supplementary Fig. 7j). In addition, and like full-body *RagA*^GTP/∆^ mice, the results of the PTT were similar in Li-*RagA*^GTP/∆^ and wild-type mice (Supplementary Fig. 7k), and also similar was the expression of hepatic gluconeogenic enzymes and *PYGL*, enzyme involved in glycogen degradation (Supplementary Fig. 7l). Glycogen was mobilized with similar kinetics in Li-*RagA*^GTP/∆^ and wild-type animals both in the liver and in the gastrocnemius muscle (Supplementary Fig. 7m). Thus, deregulated hepatic Rag GTPase signaling suffices to deteriorate glucose homeostasis without detectable effects on gluconeogenesis or glycogen mobilization.

### Proteomics analysis from Li-*RagA*^GTP^ mice

We next performed whole-proteome analysis of livers from overnight fasted-refed Li-*RagA*^GTP/Δ^ and control mice (Fig. 7c, Supplementary Data. 2), as done with full-body *RagA*^GTP/Δ^ mice (Fig. 4). From a total of 6553 detected proteins found in all samples, the levels of 212 proteins were significantly different in Li-*RagA*^GTP/Δ^ vs. control livers from fasted mice (Supplementary Fig. 7n), while the levels of only 127 proteins were significantly different in Li-*RagA*^GTP/Δ^ vs. control livers in refed conditions (Supplementary Fig. 7n). As seen in the proteomics analysis from full-body *RagA*^GTP/Δ^ mice, unbiased whole-proteome comparisons revealed that the most striking differences observed in Li-*RagA*^GTP/Δ^ vs. control livers from fasted mice were the downregulation of proteins related to fatty acid metabolism (Fig. 7d), and PPARα targets, but not TFEB targets (Fig. 7d and Supplementary Fig. 7o). The similarities of the proteomic analyses from full-body and liver-specific RagA activation (Supplementary Fig. 7p), showing a non-random overlap of differentially abundant proteins in the two systems, together with the assays with primary hepatocyte cultures, indicate that the restricted activation of Rag GTPase signaling in the liver cell-autonomously impairs PPARα-dependent transactivation and affects hepatic lipid homeostasis. Consistently, the mRNA levels of *PPARα* and several of its target genes, including those involved in ketogenesis and peroxisomes, were reduced in livers from fasted Li-*RagA*^GTP/Δ^ mice (Fig. 7e), and a similar reduction in the levels of proteins involved in mitochondrial and peroxisomal β-oxidation was found specifically in fasting conditions (Supplementary Fig. 7q, r), as observed in full-body *RagA*^GTP/Δ^ mice. Finally, ketogenesis was impaired in Li-*RagA*^GTP/Δ^ mice upon fasting (Fig. 7f), together with significantly increased levels of bile acids in circulation (Fig. 7g). Altogether, we conclude that the sole activation of Rag GTPase signaling in hepatocytes recapitulates metabolic defects and impaired adaptation to fasting observed in mice with systemic activation of RagA, dissecting a prominent role for hepatic nutrient signaling upstream of mTORC1 in the systemic control of fasting metabolism.

### Side-by-side comparison of Li-*RagA*^GTP^ and Li-*TSC1*^KO^ mice

**Fig. 8:**
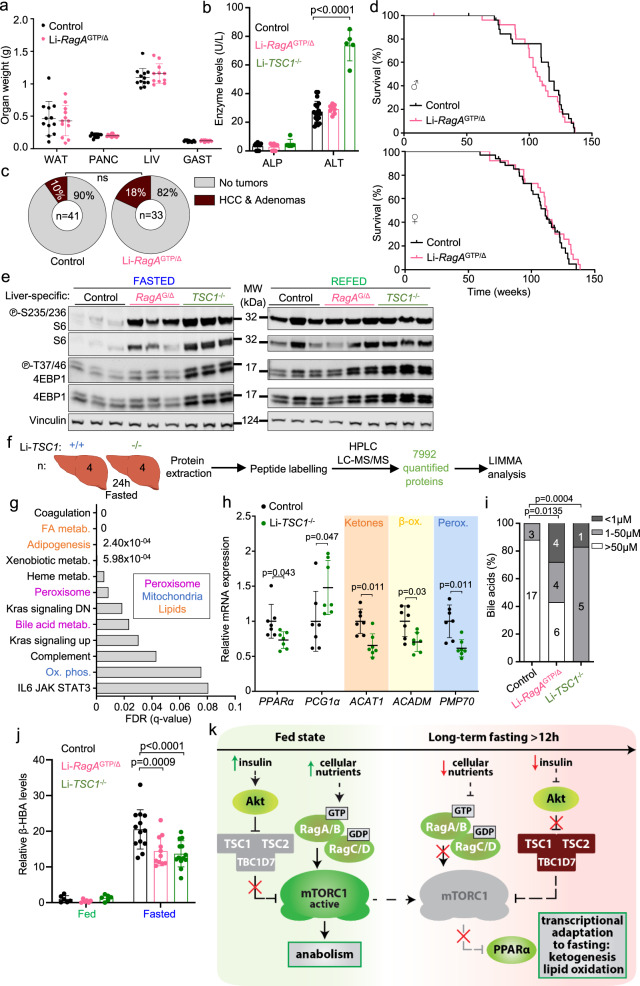
Li-*RagA*^GTP/∆^ mice share fasting metabolic alterations with Li-*TSC1*^*−/−*^ mice but do not develop hepatocellular damage nor carcinomas. **a** Weight of the indicated organs of 23- to 42-week-old control (n = 12) and Li-*RagA*^GTP/∆^ (n = 12) females. White adipose tissue (WAT), Liver (LIV), pancreas (PANC), gastrocnemius muscle (GAST). **b** Levels of circulating alanine aminotransferase (ALT) and alkaline phosphatase (ALP) were measured in 5- to 14-week-old ad libitum control (*n* = 20), Li-*RagA*^GTP/Δ^ (*n* = 8) and Li-*TSC1*^−/−^ (*n* = 6) mice. Statistical significance was calculated by using 1way ANOVA with Dunnett’s multiple comparison correction. **c** Tumor development in control (*n* = 41) and Li-*RagA*^GTP/∆^ (*n* = 33) mice. **d** Kaplan-Meier survival curve of controls (males *n* = 28, females *n* = 34) and Li-*RagA*^GTP/Δ^ (males *n* = 26, females *n* = 26). **e** 13- to-25-week-old control, Li-*RagA*^GTP/Δ^, and Li-*TSC1*^−/−^ females were deprived from food for 24 h and sacrificed following a 2 h refeeding. Protein lysates from the liver were immunoblotted for the indicated proteins. **f** Schematic summary of the proteomics experimental setting. Protein lysates from livers of 17- to 25-week-old control (*n* = 4) and Li-*TSC1*^−/−^ (*n* = 4) females, fasted for 24 h were extracted, processed and labelled as indicated. **g** Graphical representation of the false discover rates (FDRs) from the indicated hallmarks gene sets from Li-*TSC1*^−/−^ (*n* = 4) versus control (*n* = 4) livers. **h** RT-qPCR of livers from 17- to 26-week-old control (*n* = 7) and Li-*TSC1*^−/−^ (n = 7) females fasted for 24 h. Expression levels of the indicated genes relative to the average level in fed control mice. β-actin was used as housekeeping gene. Statistical significance was calculated by using unpaired two-tailed t-test corrected for multiple comparisons (Holm-Sidak). **i** Bile acid levels were measured from blood samples from 5- to 14-week-old ad libitum control (*n* = 20), Li-*RagA*^GTP/∆^ (*n* = 14), and Li-*TSC1*^−/−^ (*n* = 6) males and females. Statistical significance was calculated by using Chi-square test. **j** 12- to 27-week-old males and females in ad libitum (fed) or fasted for 24 h were bleed and the levels of β-hydroxybutyrate (β-ΗΒΑ) were quantified using NMR. Control (fed *n* = 6 and fasted *n* = 13), Li-*RagA*^GTP/∆^ (fed *n* = 6 and fasted *n* = 11) and Li-*TSC1*^−/−^ (fed *n* = 6 and fasted *n* = 16). Statistical significance was calculated by using 2-way ANOVA with Sidak’s multiple comparison correction. **k** Graphical abstract of the metabolic role of mTORC1 in adaptation to fasting. In all panels, horizontal lines indicate the mean and error bars depict Standard deviation (SD).

Constitutive activation of RagA in the liver did not lead to increased liver size (Fig. 8a), neither it increased the levels of transaminases indicative of liver damage, such as alanine amino transferase (ALT) and alkaline phosphatase (ALP) (Fig. 8b), nor resulted in the development of spontaneous liver tumors and shortened lifespan (Fig. 8c, d). These results contrast with those observed in models of deregulated growth factor-dependent activation of mTORC1 in the liver: liver damage, necrosis, fibrosis, and liver tumors^8,9^.

To understand why growth factor- versus nutrient-dependent activation of mTORC1 differentially impact hepatocellular homeostasis, while mirroring several metabolic alterations, we looked side-by-side at the extent of mTORC1 activation in Li-*RagA*^GTP/Δ^ and Li-*TSC1*^−/−^ mice. Samples obtained from fed mice had high mTORC1 activity, regardless of the genotype, and fasting resulted in highest mTORC1 activity in Li-*TSC1*^−/−^ mice, compared with Li-*RagA*^GTP/Δ^, but both growth factor- and nutrient-signaling activation resulted in higher fasting mTORC1 activity as compared to that of fasted wild-type mice (Fig. 8e and Supplementary Fig. 8a). In addition, and as previously reported^8,9^, the marker of hepatocellular damage ALT (Fig. 8b) as well as several other markers of liver damage detected by anatomopathological analysis (necrosis, fibrosis, and hepatitis) (Supplementary Fig. 8b, bottom) were increased in Li-*TSC1*^−/−^ mice and mainly absent in Li-*RagA*^GTP/Δ^ mice.

To determine, at the whole proteome level, the similarities between Li-*RagA*^GTP/Δ^ and Li-*TSC1*^−/−^ livers, we analyzed the proteome of fasted Li-*TSC1*^−/−^ livers (Fig. 8f and Supplementary Data 3). A total of 7992 proteins were detected, and, consistently with previous reports^40,47^, and in striking overlap with Li-*RagA*^GTP/Δ^ livers, fatty acid metabolism, peroxisome, bile-acid metabolism, oxidative phosphorylation, PPARα target signatures were significantly depleted from fasted Li-*TSC1*^−/−^ livers and validated by qPCR (Fig. 8g, h, and Supplementary Fig. 8c–f). Finally, the reduction of bile acids and ketone bodies upon fasting was comparable in Li-*RagA*^GTP/Δ^ and Li-*TSC1*^−/−^ mice (Fig. 8i, j). Altogether, the striking overlap of metabolic alterations observed in Li-*RagA*^GTP/Δ^ and Li-*TSC1*^−/−^ mice indicates that suppression of mTORC1 by inhibitory cues from nutrient and hormonal signaling during fasting are sufficient to coordinate the metabolism of the fasted state (Fig. 8k). However, while chronically deregulated growth factor signaling to mTORC1 drives hepatocellular damage and liver cancer, deregulated nutrient signaling upstream of mTORC1 does not overtly affect hepatocyte homeostasis, it does not result in hepatocellular tumorigenesis, neither it compromises long-term survival.

## Discussion

This work constitutes the first report of mice with constitutive RagA signaling to mTORC1 in all adult cells, a genetic system to interrogate how chronic elevation of nutrients impacts mammalian physiology by deregulating mTORC1 and without manipulating nutrient intake or hormonal signaling. These efforts were challenged in the past because constitutive expression of the *RagA*^Q66L^ allele (*RagA*^GTP^) in homozygosity lead to neonatal lethality, and heterozygous expression of this mutant allele caused no signaling activation^15^. The restriction of the expression of the *RagA*^GTP^ variant to one allele, in the absence of expression of a wild-type RagA from the other allele, together with a different genetic background, allowed the rescue of the aforementioned neonatal lethality (Fig. 1a). This rescue shows that subtle changes in the extent of increased nutrient signaling have dramatic consequences in mice: i.e. developmental aberrations and fully-penetrant neonatal lethality versus a 60% survival with mild and stereotypical morphometric alterations.

Genetically engineered mice for studying the consequences of systemic activation of mTORC1 have frequently faced the issue of embryonic lethality, as in *TSC1/2* KO mice^4^ and *Pten* KO mice^48,49^. Conditional, tissue-specific activation has provided substantial pathophysiological insight on the in vivo functions of mTORC1 and its deregulation by hormonal cues. The rescue of neonatal lethality in *RagA*^GTP^ mice allowed the study of systemic activation of nutrient signaling in adult mice. These mice exhibited obvious phenotypic alterations, including stereotypical cranial abnormalities and hypopigmentation, two clinical features present in syndromes of deregulated mTORC1 activity^19,23^, and interestingly, in a familial hypomorphic variant of a Ragulator component^50^. Thus, both gain- and loss-of-function alterations in nutrient signaling can impair skin and hair pigmentation.

The signaling perturbation on mTORC1 generated by constitutive activation of nutrient signaling is seen in starved cells and in fasted mice (Fig. 1) but does not increase maximal activity of mTORC1 in nutrient replete conditions. Thus, the consequences of constitutive nutrient signaling are likely to affect only physiological responses linked to fasted state. In addition, the extent of signaling activation was not identical in every organ from *RagA*^GTP^ mice: while liver and skeletal muscle showed a comparable state of mTORC1 activation in fasted vs. fed states, kidney, heart, WAT, and BAT from *RagA*^GTP^ mice show a partial inhibition of mTORC1 in the fasted state (Fig. 1c and Supplementary Fig. 1e, f). The differential impact on mTORC1 by the same genetic perturbation is unlikely to be caused by a compensatory effect of very low levels of RagB, and most likely reflects the dependence on a normal regulation of hormonal signaling, not genetically manipulated in *RagA*^GTP^ mice.

While *RagA*^GTP/GTP^ neonates experience a lethal metabolic crisis during the first episode of nutrient limitation occurring immediately after the interruption of transplacental supply of nutrients that occurs with birth^15^, the metabolic consequences of endogenous expression of *RagA*^GTP/Δ^ in adult mice are comparatively milder. Constitutive nutrient signaling in adult mice affects the adaptation to fasting, but mice do not succumb or experience a severe hypoglycaemic state. In contrast, the levels of circulating glucose under fasting resulted abnormally high and *RagA*^GTP^ mice exhibited a moderate glucose intolerance with an elevated HOMA-IR index (Fig. 3). The Framingham epidemiology study^31^ showed that elevated levels of circulating branched chain amino acids are the best predictors of future development of type 2 diabetes in healthy, lean individuals. Our results provide experimental support, by genetic means in mice, for an emerging idea that systemically high branched chain amino acids, via Rag GTPase - mTORC1 activation, may perturb glucose homeostasis. This moderate loss of glycaemic control did not correlate with an obvious inhibition of hepatic PI3K-Akt signaling, decreased glucose uptake or overt changes in hepatic glucose output. We cannot exclude the provocative possibility of increased gluconeogenesis in *RagA*^GTP/Δ^ mice from amino acids such as glutamine (the most abundant amino acid in circulation), whose levels are reduced in *RagA*^GTP/Δ^ mice.

Global proteomic analysis of liver samples from wt and *RagA*^GTP^ mice revealed the expected features consistent with increased nutrient signaling. These include mTORC1 activation itself, proteins encoded by TOP mRNAs (mostly involved in protein synthesis), PPARα targets; but surprisingly failed to identify a strong suppression of TFEB activity^51,52^. A potential technical explanation for this is the enrichment for transcriptional targets of TFEB translate to membrane-bound proteins with lysosomal localization, which tend to be under-represented using standard proteomic approaches. A biological explanation is that inhibitory phosphorylation of TFEB, a target of mTORC1 with unique sensitivity to Rag GTPase complex activity^37,53^, may be more strongly controlled by the nucleotide state of RagC and less so on that of RagA, used herein. Collectively, our whole proteomic analysis revealed a striking inability of *RagA*^GTP^ mice to unleash the fasting responses executed by PPARα, which normally include ketogenesis, fatty acid oxidation with increased respiratory capacity in the mitochondria, amino acid catabolism, and peroxisome abundance. Hence, under fasting conditions, the metabolic alterations in mice with constitutive nutrient signaling to mTORC1 include glucose, lipid, ketone, and amino acid metabolism, a wide range of metabolic defects that highlight the central role of the dynamic regulation of mTORC1 for metabolic homeostasis. In addition, the abnormal systemic metabolic state of fasted *RagA*^GTP^ mice is reflected with increased energy expenditure and increased weight loss, and a respiratory quotient indicative of a shift to sugar metabolism to detriment of fat/ketone metabolism (Fig. 5g). While a deficient PPARα axis largely explains the aberrant fasting metabolism of mice with constitutive nutrient signaling, the fasting hyperglycaemia contrasts with the hypoglycaemia that is observed in *PPARα* KO mice^42^, pointing to PPARα-independent metabolic defects at work in *RagA*^GTP^ mice.

An unmet expectation for an outcome of constitutive nutrient signaling systemically was a premature aging phenotype, as seen in other model organisms^54,55^, but which did not seem to occur in *RagA*^GTP^ mice, as more severe pathologies precipitate before a moderate acceleration of aging can manifest (Fig. 2). This difference, which is not unprecedent as other models of increased mTORC1 activity also succumb before physiological longevity can be scored^10^, precludes still pending efforts to understand the underpinnings of the connections between increased mTORC1 activity and aging.

Importantly, liver-specific activation of Rag GTPase signaling did not recapitulate developmental and morphometric alterations, but partially mirrored (Fig. 7) the metabolic alterations observed in full-body *RagA*^GTP^ mice. Li-*RagA*^GTP/Δ^ mice exhibited loss of glucose tolerance, but not the fasting hyperglycaemia observed in full-body *RagA*^GTP^ mice. Moreover, whole proteome analysis confirmed the outstanding suppression of the PPARα transcriptional program, including mitochondrial, peroxisomal and lipid metabolism, and the systemic defects in ketogenesis and increased bile acids levels.

Li-*TSC1*^−/−^ mice and Li-*RagA*^GTP/Δ^ largely shared metabolic alterations, but Li-*RagA*^GTP/Δ^ mice did not suffer the overt, aberrant hepatocellular damage and spontaneous tumor development seen in Li-*TSC1*^−/−^ mice (Fig. 8). This milder phenotype correlated with the smaller extent of activation of mTORC1 in Li-*RagA*^GTP/Δ^ versus Li-*TSC1*^−/−^ samples compared side-by-side, and also correlated with the extent of indirect inhibition of Akt, which is stronger in *TSC1/2*-deficient cells and mice. Thus, these results suggest that deregulated growth factor signaling more profoundly affects hepatocellular homeostasis, and that the degree of chronic overactivation of mTORC1 may result in either benign or pathological outcomes. Although speculative, these differences may underlie the existence of frequent genetic alterations in growth factor signaling upstream of mTORC1 in human cancer, being mutations in the nutrient signaling cascade more infrequent and found mostly in B cell lymphomas^56^.

Finally, by putting together the phenotypes of Li-*RagA*^GTP^ mice, Li-*TSC1* KO mice and those of hepatic deletion of Deptor, a negative Regulator of mTOR, the involvement of mTORC1 inhibition in the control fasting metabolism is likely biphasic: (1) an early post-absorptive state (6 h to 12 h after food intake) where mTORC1 is inhibited in a Deptor-dependent manner^57^, which contributes to modulate an rapid drop in glycaemia; followed by (2) a complete fasting response driven by overlapping nutrient/Rag GTPase- and growth-factor/TSC-mediated inhibition of mTORC1, in turn enabling the PPARα transcriptional program and metabolic adaptation (Fig. 8k).

## Methods

### Generation of *RagA*^GTP/GTP^ and *RagA*^GTP/∆^ mice

All animal procedures carried out at the CNIO were performed according to protocols approved by the CNIO-ISCIII Ethics Committee for Research and Animal Welfare (CEIyBA) and the Autonomous Community of Madrid (CAM). Protocol numbers PROEX285/15 and PROEX15/18. Mice were housed under specific pathogen free conditions at 22 °C and with 12-h dark/light cycles. *RagA*^LSL-Q66L^ mice were generated in the David M Sabatini laboratory at the Whitehead Institute for Biomedical Research at Massachusetts Institute of Technology^15^. Expression of the *RagA*^Q66L^ allele (also referred to as *RagA*^GTP^) only occurs upon Cre-dependent elimination of the transcriptional STOP cassette on the null *RagA*^LSL-Q66L^ locus. Hemizygous *RagA*^Q66L/LSL-Q66L^ (referred to as *RagA*^GTP/Δ^) mice were generated by crossing *RagA*^Q66L/+^ mice with mice carrying the null allele *RagA*^LSL-Q66L^. To minimize genetic drift and to maintain maximum genetic representation in the mixed background in which neonatal viability occurred, experimental cohorts were always from F2 generation. For hepatocyte-specific activation of RagA, we produced mice that expressed the *RagA*^Q66L^ variant, in the absence of wild-type RagA, exclusively in the liver. Thus, we crossed *RagA*^GTP/+^ mice with *RagA*^floxed/floxed^; Albumin-Cre transgenic mice^58^ to generate *RagA*^floxed/GTP^; Albumin-Cre^Tg^ mice. Mice with normal Rag GTPase signaling used as controls were *RagA*^GTP/floxed^ without Albumin-Cre allele, and *RagA*^GTP/+^; Albumin-Cre^Tg^ mice. 

### Animal procedures

For hepatocyte-specific deletion of *TSC1*, we crossed *TSC1*^floxed/floxed^ mice^5^ with Albumin-Cre transgenic mice to generate Li-*TSC1*^−/−^ mice. Mice were housed under specific pathogen-free (SPF) conditions, at 22 C, and with 12-hr dark/light cycles. Mice were fed with a standard chow diet (Harlan Teklad 2018). For fasting experiments of overnight or 24 h, mice were placed in a clean cage without access to food from 4 pm to 8 am or form 8 am to 8 am next day, respectively. In each cage a maximum of two animals were placed. Unless otherwise stated in the legends, samples were obtained from fasted mice as described above with and without *ad libitum* feeding with chow diet for 30 min or 2 h. For the 48 h fasting, the same protocol was used, and fasting was started at 7:30 am. Body weight was monitored every 6 h to avoid a weight loss higher than 20%. Glucose was monitored with Accu-check Aviva glucometer by making a small incision at the end of the tail to obtain a drop of blood. For survival experiments, mice were observed weekly by trained personnel until they presented signs of morbidity, time at which mice started to be inspected daily until application of Humane End Point (HEP) criteria (https://grants.nih.gov/grants/olaw/guide-for-the-care-and-use-of-laboratory-animals.pdf) in consultation with veterinary staff blinded to the mouse genotype or procedure. Neonates were obtained by caesarean section at E19.5 and placed on ice for sacrificing. To prevent early delivery, pregnant mums were injected with 2 mg of progesterone (Sigma P0130) in PBS once a day at E17.5 and 18.5. For measuring the length, mice were anesthetized via gaseous anesthetic (isoflurane) with a continuous flow of 1 to 3% isoflurane/oxygen mixture (2 L/min), for approximately 5 min. The nose-to-tail length was measured with a caliper. Rapamycin was administered in food, encapsulated in chow diet at 42 ppm (Rapamycin Holdings and Purina Lab Diet). Before the sacrifice, mice were fasted overnight and injected intra-peritoneally (IP) with 2 mg/kg of Rapamycin (Alpha Aesar J62473) dissolved in 5% Tween 80 (Sigma P7949) + 5% PEG400 (Fluka 81172).

### Glucose tolerance test

Mice were fasted from 4 pm to 8 am as previously indicated. Before injecting the mice, around 20 μL of blood was extracted from the tail to measure insulin levels and calculate the HOMA-IR. Following fasting, D-Glucose 2 g/Kg (Sigma Aldrich G7528) was injected intraperitoneally and glucose levels were measured at 15, 30, 45, 60, 90 and 120 min after injection.

### Insulin Tolerance test (ITT)

Pure C57BL/6 mice were fasted from 9 am to 3 pm; mixed C57BL/6:129 Sv background mice were fasted for 3 h starting at 12 pm, due to an intrinsic increase in insulin sensitivity. After the fasting, mice were injected intraperitoneally with 0.75 U/Kg of mice of insulin (Humulin R U-100 Lilly) in PBS and tail blood glucose levels were measured at 15, 30, 45, 60, 90 and 120 min after injection.

### Pyruvate tolerance test (PTT)

Mice were fasted from 4 pm to 8 am as previously indicated. Sodium pyruvate (1.5 g/Kg body weight; Sigma Aldrich G7528) was injected intraperitoneally, and tail blood glucose levels were measured at 15, 30, 45, 60, 90, and 120 min after injection.

### Indirect calorimetry

EE, RQ, food intake, voluntary movement (rearing), was measured with Oxylet device from Panlab. Briefly, mice were first acclimated for 5d. This was followed by individualization and acclimation to the metabolic cages for at least 3d. After the acclimatization period, the experiment was started. The animals had unrestricted access to food for 2 days and 3 nights, followed by ~27 h fasting. Weight and glucose were monitored before and after the ad libitum and the fasting conditions. Lean mass, obtained from densitometric analysis before the experiment, was used instead of the total body weight for normalization of values.

### Animal imaging

For densitometry analysis, body composition measurements (body weights, fat mass, and lean muscle mass) was measured using dual-energy X-ray absorptiometry (DEXA) PIXImus, Mouse Densitometer (GE Lunar co, Madison, WI, USA), software version 1.46. Mice were anesthetized with isoflourane during recording. Quality control was performed using a calibrated phantom before imaging. For microCT scans mice were anesthetized and the area of interest was imaged with the eXplore Vista micro-CT scanner (GE Healthcare, London, Canada). The isotropic resolution of this instrument is 45 µm. The micro-CT image acquisition consisted of 400 projections collected in one full rotation of the gantry in approximately 10 min. The X-ray tube settings were 80k V and 450 µA. For skull imaging analysis, CT gantry was centered at the skull and the quantification was performed using Microview 2.5.0 software (GEHC; London, Canada). For esophageal imaging, the image acquisition was performed immediately after intraesophageally administration of a contrast agent Iopamiro 0.28 ml per mice (Bracco, Milan Italy) at a concentration 0.612 mg/ml by oral gavage. The gantry was centered in the thorax and upper part of the abdomen. The reconstructed images were viewed and analyzed using 3D Slicer 4.10.1 software https://www.slicer.org/.

### Ex vivo muscle [^3^H]-2-Deoxy-d-glucose uptake

For ex vivo muscle [^3^H]-2-deoxy-d-glucose uptake, mice were fasted overnight and rapidly after cervical dislocation soleus and extensor digitorum longus (EDL) muscles from both legs were dissected out and incubated for 60 min at 37 C in Krebs-Ringer bicarbonate (KRB) solution containing the following (in mmol/L): 117 NaCl, 4.7 KCl, 2.5 CaCl_2_·2H_2_O, 1.2 KH_2_PO_4_, 1.2 MgSO_4_·7H_2_O, and 24.6 NaHCO_3_ and supplemented with 2 mmol/L pyruvate and 0.1% of BSA. Glucose transport was assessed during 10 min at 37 C using 6.5 mM 2-DOG containing 1.5 μCi/ml 2-deoxy-D-[^1, 2-3^H]-glucose (Perkin Elmer NET549A250UC) and 7 mM mannitol containing 0.3 μCi/ml D-[^14^C]-mannitol (Perkin Elmer NEC852050UC) in KRB. After the incubation, muscles were washed once in cold PBS and dried on Whatman paper. Then, flash-frozen in liquid nitrogen, weighed, and solubilized for 30 min at 65 C in 300 μl of 1 M NaOH. After centrifugation at 10,000 g for 10 min, 100 µL aliquots were taken for scintillation counting of the [^3^H] and [^14^C] labels using a Perkin Elmer *Wallac 1414* Liquid Scintillation counter. [^14^C]-mannitol was used to correct the amount of extracellular [^3^H]-2-DG that was non-specifically uptaken. Counts were normalized to muscle weight.

### Hepatic glycogen content

Glycogen was measured in liver samples^59^. Briefly, glycogen was extracted from livers in 30% KOH saturated with Na_2_SO_4_, precipitated in 95% ethanol and re-suspended in double-distilled H_2_O. After the addition of phenol and H_2_SO_4_, absorbance at 490 nm was measured in duplicates.

### Sample preparation for proteomic analysis

Liver samples were lysed 10 min at 95 C in 5% SDS, 50 mM TEAB pH 7.55. After cooling, DNA was sheared by 10 min of sonication. Protein concentration was determined using micro BCA using BSA as standard. Fifty µg of each sample were digested by means of the Protifi™ S-Trap™ Mini Spin Column Digestion Protocol. Briefly, proteins were reduced and alkylated (15 mM TCEP, 25 mM CAA) 1 h at 45 C in the dark. SDS was removed from samples in the S-Trap column using 90% methanol in 100 mM TEAB and proteins were digested with 125 µl of trypsin in 50 mM TEAB pH 7.55 (Trypzean, Sigma, protein:enzyme ratio 1:25, 16 h at 37 C). The resulting peptides were eluted from S-Trap columns, speed-vac dried, and re-dissolved in 20 µl of 100 mM HEPES pH 8.0. 50 µg per sample were labelled using Thermo Scientific TMT11plex™ Isobaric Label Reagent Set following manufacturer’s instructions. Samples were mixed in 1:1 ratio based on total peptide amount, which was determined from an aliquot by comparing overall signal intensities on a regular LC–MS/MS run. The final mixture was finally desalted using a Sep-Pak C18 cartridge (Waters) and dried prior high pH reverse phase HPLC pre-fractionation.

### High pH reverse phase chromatography for proteomics analysis

Peptides were pre-fractionated offline by means of high pH reverse phase chromatography using an Ultimate 3000 HPLC system equipped with a sample collector. Briefly, peptides were dissolved in 100 µL of phase A (10 mM NH_4_OH) and loaded onto a XBridge BEH130 C18 column (3.5 µm, 150 mm length and 1 mm ID) (Waters). Phase B was 10 mM NH_4_OH in 90% CH_3_CN. The following gradient (flow rate of 100 µL/min) was used: 0–50 min 0–25% B, 50–56 min 25–60% B, 56–57 min 60–90% B. One-minute fractions from minute 15 to 65 were collected, neutralize with 10 µl of 10% formic acid and immediately vacuum dried. Based on the UV absorbance at 280 nm, 35 fractions were selected for LC-MS/MS analysis.

### Mass spectrometry for proteomics analysis

LC-MS/MS was done by coupling an UltiMate 3000 RSLCnano LC system to a Q Exactive HF mass spectrometer (Thermo Fisher Scientific). Five microliters of peptides were loaded into a trap column (Acclaim™ PepMap™ 100 C18 LC Columns 5 µm, 20 mm length) for 3 min at a flow rate of 10 µl/min in 0.1% FA. Peptides were then transferred to an EASY-Spray PepMap RSLC C18 column (Thermo) (2 µm, 75 µm × 50 cm) operated at 45 C and separated using a– 60 min effective gradient (buffer A: 0.1% FA; buffer B: 100% ACN, 0.1% FA) at a flow rate of 250 nL/min. The gradient used was, from 4 to 6% of buffer B in 2 min, from 6 to 33% B in 58 min, plus 10 additional minutes at 98% B. The mass spectrometer was operated in a data-dependent mode, with an automatic switch between MS and MS/MS scans using a top 12 method. (Intensity threshold ≥ 9.3e4, dynamic exclusion of 20 s and excluding charges unassigned, +1 and ≥ +6). MS spectra were acquired from 350 to 1500 m/z with a resolution of 60,000 FMHW (200 m/z). Ion peptides were isolated using a 1.0 Th window and fragmented using higher-energy collisional dissociation (HCD) with a normalized collision energy NCE of 35. MS/MS spectra were acquired with a fixed first mass of 100 m/z and a resolution of 45,000 FMHW (200 m/z). The ion target values were 3e6 for MS (maximum IT of 25 msec) and 1e5 for MS/MS (maximum IT of 86 msec).

### Proteomic data analysis

Raw files were processed with MaxQuant (v 1.6.10.43) using the standard settings against a mouse protein database (UniProtKB/TrEMBL, 53,449 sequences) supplemented with contaminants. Carbamidomethylation of cysteines was set as a fixed modification whereas oxidation of methionines, protein N-term acetylation, and N/Q de-amidation as variable modifications. Minimal peptide length was set to 7 amino acids and a maximum of two tryptic missed-cleavages were allowed. Results were filtered at 0.01 FDR (peptide and protein level). Afterwards, the “proteinGroups.txt” file was loaded in Prostar (v1.18)^60^ using the intensity values for further statistical analysis. Briefly, proteins with less than eleven valid values were filtered out. Global normalization of log2-transformed intensities across samples was performed using the LOESS function. Differential analysis was done using the empirical Bayes statistics Limma 3.46.0. Proteins with a *p*-value < 0.05 and a log2 ratio >0.3 or < −0.3 were defined as regulated. The FDR was estimated to be below 5% by Benjamini–Hochberg. GSEA Pre-ranked (broad institute) was used to perform gene-set enrichment analysis of the described gene signatures on a pre-ranked gene list, setting 1000 gene-set permutations. GSEA Pre-ranked calculates an enrichment score for each gene set using the Kolmogorov–Smirnov test. The nominal P-value estimates the statistical significance of the enrichment score for a single gene set and it was corrected for gene-set size and multiple hypothesis testing.

### Immunoblotting

For cell experiments, cells were rinsed once with ice-cold PBS and lysed in ice-cold lysis buffer (50 mM HEPES (pH 7.4), 40 mM NaCl, 2 mM EDTA, 1.5 mM sodium orthovanadate, 50 mM NaF, 10 mM pyrophosphate, 10 mM glycerophosphate and 1% Triton X-100 and one tablet of EDTA-free complete protease inhibitors (Roche) per 25 ml). Cell lysates were cleared by centrifugation at maximum speed for 10 min. For tissue extraction, a small piece of previously snap-frozen tissue was homogenized in ice-cold lysis buffer using the suggested homogenization protocols of the FastPrep machine (FastPrep-24^TM^ 5 G). Tissue lysates were cleared by centrifugation at maximum speed for 10 min. Protein content was measured with BCA (Bicinchoninic Acid) Protein Assay (Thermo Scientific™ 23222), protein was denatured by the addition of sample buffer, boiled for 5 min, resolved by SDS–PAGE and analyzed by immunoblotting. Western blot analyses were performed according to standard procedures. Antibodies were visualized by using Odyssey Infrared Imaging System (Application software version 3.0.30) LI-COR Biosciences. Antibodies from Cell Signaling Technology were used for detection of P-T389-S6K1 (#9234) dilution: 1/500, S6K1 (#2708) dilution: 1/500, P-S235/236-S6 (#2211) dilution: 1/1000, S6 (#2217) dilution: 1/1000, P-T37/46-4EBP1 (#2855) dilution: 1/500, 4EBP1 (#9644) dilution: 1/1000, Catalase (#14097 S) dilution: 1/1000, RagA (#4375 S) dilution: 1/500, RagC (#9480) dilution: 1/500, P-S473-AKT (#4060) dilution: 1/500, P-T308-AKT (#2965) dilution: 1/500, AKT (#4691) dilution: 1/1000, P-T246-PRAS40 (#2997) dilution: 1/1000, PRAS40 (#2691) dilution: 1/1000, P-S9-GSK3β (#9336) dilution: 1/500, GSK3β (#9315) dilution: 1/1000, P-T24/32-FOXO1/3 (#9464) dilution: 1/500, FOXO1 (#2880) dilution: 1/500, P-S588-AS160 (#8730) dilution: 1/1000, AS160 (#2670) dilution: 1/500. Antibodies from Proteintech were used to detect Pex14 (#10594-1-AP) dilution: 1/500, Pex16 (#14816-1-AP) dilution: 1/500 and Pex19 (#14713-1-AP) dilution: 1/500. Anti-PMP70 (#SAB4200181) dilution: 1/1000, anti-thiolase (#HPA007244) dilution: 1/1000, anti-β-actin (#A1978) dilution: 1/5000 and anti-vinculin (#V9131) dilution: 1/5000, were obtained from Sigma. The uncropped version of the western blots showed in the manuscript can be found in the source data file.

### Histological stainings

Tissue samples were fixed in 10% neutral buffered formalin (4% formaldehyde in solution), and samples were then embedded in paraffin and cut at 3μm, mounted in super frost plus slides and dried overnight. Slides were deparaffinized in xylene and rehydrated through a series of graded concentrations of ethanol in water, then paraffin sections were stained with hematoxylin and eosin (H&E), Periodic Acid Schiff (PAS) for detection of glycogen and other carbohydrates, and Sirius red for fibrosis were also performed. Positive control sections known to be primary antibody positive were included for each staining run. Whole slides were acquired with a slide scanner (AxioScan Z1, Zeiss), images captured with the Zen 2.3 Blue edition Software (Zeiss) and quantified with ImageJ (1.52a).

### Determination of aspiration pneumonia

Pneumonia was diagnosed by the presence of multiple patches in the distal pulmonary parenchyma, particularly around the bronchioles. Typical foci had neutrophilic exudates, macrophages and karyorrhectic debris, filling alveolar spaces, admixed with red cells. Isolated or aggregated lymphocytes were observed around groups of epithelioid macrophages forming granulomas. Inside or in the periphery of these granulomas, foreign vegetable material was observed usually surrounded by multinucleated giant cells, macrophages, lymphocytes and neutrophils sometimes with micro-abscess formation. There was also mild proliferation of fibroblasts on the periphery of lesions. Additionally, areas of collapsed bronchial tube were concomitant to areas of emphysema. The observation of dilated esophagus, mucus in the respiratory tract and presence of foreign vegetable material in the bronchus of several animals lead to the diagnose of aspiration pneumonia.

### Assessment of liver damage

Liver necrosis was diagnosed by the observation of single-cell necrosis or small groups of hepatocytes presenting an increased and pale cytoplasm, with a small or fragmented hyperchromatic nucleus (pyknosis), sometimes surrounded by inflammatory cells. The diagnose of hepatitis was done by the observation of mononucleated or polymorphonucleated inflammatory cells including, lymphocytes, plasmocytes, macrophages, neutrophils, or eosinophils in multi-focal areas of the liver. In these cases, could or could not be accompanied by degeneration or necrosis of the hepatocytes, with or without fibrosis. Liver fibrosis was determined by the observation of excess fibrous connective tissue between contiguous periportal spaces in the hematoxylin and eosin (H&E) staining. Fibrosis was corroborated, when needed, by the observation of collagen fibers in red color in the Sirius red staining specifically distributed surrounding the hepatocytes located in the periportal space or in the middle of the hepatic parenchyma. Hepatocellular carcinomas were diagnosed upon the observation of a disorganized proliferation of hepatocytes with different degrees of cellular atypia forming nodular lesions in which the normal structure of the liver was lost and portal spaces and centrilobular veins were no longer observed.

### Quantitative PCR

Frozen tissue total RNA was extracted using Trizol (Life Technologies 15596-018) according to manufacturer’s instructions. Samples were retro-transcribed with SuperScript IV (Invitrogen 11756500). Real-time PCR was run in triplicates using GoTaq qPCR mastermix (Promega A6001) with a reaction volume of 10 μL. β-actin was used for normalization. Primer sequences are listed in Supplementary Table 2.

### Quantification of metabolites and insulin from plasma

Plasma metabolites were measured using the following commercial kits according to manufacturer’s instructions: Blood Urea Nitrogen (BUN), alanine aminotransferase (ALT), alkaline phosphatase (ALP), and bile acids (VetScan Abaxis Model 200–1000 and Abaxis Europe GMBH 500-0040-12). Free fatty acids (WAKO kit 434-91975, 434-6-91995, 270–77000). Insulin levels (CRYSTAL CHEM INC. 90080). HOMA-IR was calculated according to the formula: fasting insulin (µIU/L) × fasting glucose (mg/dL)/405. β-hydroxybutyrate levels were measure with Sigma-Aldrich MAK134 and the relative abundance of β-hydroxybutyrate from mouse serum was quantified with nuclear magnetic resonance. Triglyceride levels were quantified with Cayman 10010303.

### Targeted LC–MS metabolites analyses

For metabolomic experiments, mice were fasted for 16 h and maintained fasted or allowed to access food ad libitum for 2 h. For serum analysis, mice were bled before the fasting period and when euthanized. Blood samples were obtained in EDTA-free tubes. Serum was obtained after centrifugation of the blood at 10,000 g for 5 min. Liver samples were immediately snap-frozen in liquid nitrogen upon euthanasia. For the LC-MS analyses metabolites were extracted as follows^61^. Extraction solution was composed of 50% methanol, 30% ACN, and 20% water. The volume of extraction solution added was adjusted to mass of the tissue (1 ml per 40 mg) or serum volume (200ul per 10ul of serum). After addition of extraction solution, samples were vortexed for 5 min at 4 C and then centrifuged at 16,000 g for 15 min at 4 C. The supernatants were collected and stored at −80 C until analyses. LC/MS analyses were conducted using QExactive Plus Orbitrap mass spectrometer equipped with an Ion Max source and a HESI II probe and coupled to a Dionex UltiMate 3000 UPLC system (Thermo). External mass calibration was performed using the standard calibration mixture every 7 d as recommended by the manufacturer. 5 μl of each sample was injected onto Zic‐pHilic (150 mm × 2.1 mm i.d. 5 μm) with the guard column (20 mm × 2.1 mm i.d. 5 μm) (Millipore) for the liquid chromatography separation. Buffer A was 20 mM ammonium carbonate, 0.1% ammonium hydroxide (pH 9.2); buffer B was acetonitrile. The chromatographic gradient was run at a flow rate of 0.200 μl/min as follows: 0–20 min; linear gradient from 80 to 20% B; 20–20.5 min; linear gradient from 20 to 80% B; 20.5–28 min: hold at 80% B^61^. The mass spectrometer was operated in full scan, polarity switching mode with the spray voltage set to 2.5 kV, the heated capillary held at 320 C. The sheath gas flow was set to 20 units, the auxiliary gas flow was set to 5 units, and the sweep gas flow was set to 0 unit. The metabolites were detected across a mass range of 75–1000 m/z at a resolution of 35,000 (at 200 m/z) with the AGC target at 106, and the maximum injection time at 250 ms. Lock masses were used to ensure mass accuracy below 5 ppm. Data were acquired with Thermo Xcalibur 4.0.27.13 software (Thermo). The peak areas of metabolites were determined using Thermo TraceFinder 3.3 SP1 software (Thermo), identified by the exact mass of each singly charged ion and by known retention time on the HPLC column. For heatmap representation, http://www.heatmapper.ca/expression/^62^ was used.

### Mouse embryonic fibroblasts (MEFs)

MEFs from E13.5 were prepared by chemical digestion with trypsin (Gibco, 25300-054) for 15 min, followed by mechanical disaggregation with surgical blades^63^. For amino acid deprivation, sub-confluent cells were rinsed twice and incubated in RPMI without amino acids, and supplemented with 10% dialyzed FBS^64^. Stimulation with amino acids (with concentration as in RPMI) was performed for 10 min.

### Primary hepatocytes

Primary hepatocytes were isolated by perfusion (Masterflex C/L 77120-62 60RPM) with collagenase type I (Sigma-Aldrich C3867). Briefly, mice were anesthetized with Ketamine/Xylazine (Merial laboratorios, SA: Imalgene® 100 mg/ml injectable solution; Bayer: Rompun® 20 mg/ml injectable solution) in saline solution, 100 mg/kg and 10 mg/kg respectively, the abdomen was opened and the aorta and the cava vein in the abdominal cavity were tied with a loose knot. Left atrium of the heart was cut, and the aorta was cannulated. The liver was then perfused with 25 mL of Buffer A at 37 C and the mouse was placed under heating red-light lamp. At the same time the abdominal knot was tight and the aorta, the cava vein and the portal vein were cut. Subsequently, liver was perfused with 25 mL of Buffer B at 37 C. After the perfusion, the liver was placed in a 50 ml falcon containing 25 mL of Buffer C and once under the hood the liver was disaggregated and filtered through 100 μm sterile cell strainer. Hepatocytes were centrifuged at 50 g for 5 min, supernatant was removed, and the pellet was resuspended in a gradient solution (50% v/v of Buffer C and 50% v/v of Buffer D). After centrifugation at 50 g for 10 min, the supernatant was removed, and the remaining pellet was washed once with Buffer C and centrifuged 5 min at 50 g. Hepatocytes were resuspended in fresh Buffer C and cells were counted with trypan blue to assess viability. For immunoblotting experiments cells were plated at 3 × 10^5^ cells/well in a collagen-coated (Sigma-Aldrich C3867) multi six-well plate. After 6 h the media was changed and hepatocytes were cultured overnight before any experimental procedure. The next day, cells were washed 3 times and then incubated in DMEM:F12 without amino acids (USBiological Life Science D9807-10) supplemented with 6 mM of NaHCO_3_ (Sigma 1063291000), 18 mM of Hepes pH 7.4 (Lonza 17-737E), 25 mM of glucose (Sigma G8769) and 10% dialyzed fetal bovine serum and let sit for 16 h after which cells were collected for protein extraction. Buffer A: Hank Balanced Salt Solution (HBSS) 1x w/o Ca^2+^ Mg^2+^ (Life Technologies 14175-053), with 10 mM of Hepes pH 7.4 (Lonza 17-737E) and 0.2 mM of EGTA (Sigma-Aldrich E3889). Buffer B: Williams E media (Gibco 22551022) with 10 mM of Hepes pH 7.4 (Lonza 17-737E). Buffer C: DMEM:F12 media (Thermo Fisher 41965039 and Thermo Fisher 21765029) with 3 µM of BSA fatty acid-free 0.02% (Sigma A6003-10G), 6 mM of NaHCO_3_ (Sigma 1063291000), 5 mM of Sodium Pyruvate 100 mM (Sigma S8636-100ml) 18 mM of Hepes pH 7.4 (Lonza 17-737E), 2 mM of L-Glutamine (SIGMA G7513-100ML), 10% FBS and antibiotics (Pen/Strep (Gibco 15070-063)) and gentamycin (Solmeglas L0012-010). Buffer D: 90% v/v of Percoll (GE Healthcare 17-0891-01) and 10% v/v HBSS 10x (Thermo Fisher 14185052).

### Seahorse analysis

Hepatocytes were plated in a collagen-coated (Sigma-Aldrich C3867) seahorse XF96 cell culture microplates at 5000 cells/well in 80 µL in Buffer C and incubated at 37 °C for 6 h. After 6 h media was changed, and hepatocytes were cultured overnight before any experimental procedure. Mitochondrial measurements and FAO were performed using the Seahorse XF Cell Mito Stress Test (Agilent 103015-100) according to manufacturer’s instructions. Briefly, primary hepatocytes were plated as indicated above and the following morning regular media was replaced for Seahorse XF DMEM medium (Agilent 103575-100) supplemented with 10 mM glucose (Agilent 103577-100), 1 mM Sodium pyruvate (Agilent 103578-100) and 2 mM glutamine (Agilent 103579-100). Oxygen consumption Rate (OCR) was measured following the sequential addition of 40 µM of Etomoxir (MedChemTronica HY-502002; port A), 1.5 µM of oligomycin (port B), 0.3 µM of carbonyl cyanide 4-(trifluoromethoxy)-phenylhydrazone (FCCP)(port C) and 0.5 µM of Antimycin/Rotenone (Rot/AA) (port C). The oligomycin, FCCP and the Rot/AA were purchase from Agilent (103015-100). Oxygen consumption rate (OCR) was normalized to protein concentration using BCA Protein Assay (Thermo Scientific™ 23222). All parameters were represented as normalize OCR.

### Statistics

Statistical analysis was carried out with Prism 8 (GraphPad). All experiments that include a second variable (e.g.: time or nutritional status) were analyzed using 2-way ANOVA with Sidak’s multiple comparison post-test. Correction for repetitive measures was performed where appropriate, and the area under the curve (AUC) was calculated. Unpaired Student’s t-test was used for single comparisons, Chi-square test (http://vassarstats.net/newcs.html) was used for comparing categorical parameters, Log-rank (Mantel-Cox) test was used for survival curves, 1-way ANOVA (Dunnett’s multiple compassion test) was used when more than one genotype was analysed. For proportional diagram representation and statistic calculation the online tools https://www.biovenn.nl/index.php^65^ and http://nemates.org/MA/progs/overlap_stats.html were used. All error bars in all panels depict Standard deviation (SD).

### Reporting summary

Further information on research design is available in the Nature Research Reporting Summary linked to this article.

## Supplementary information

Supplementary Information

Description of Additional Supplementary Files

Supplementary Data 1

Supplementary Data 2

Supplementary Data 3

Reporting Summary

## Data Availability

Metabolomics data have been deposited to the EMBL-EBI MetaboLights database (https://www.ebi.ac.uk/metabolights/) with the identifier MTBLS2397. The mass spectrometry proteomics data have been deposited to the ProteomeXchange Consortium (http://proteomecentral.proteomexchange.org/cgi/GetDataset?ID=PXD023735). The authors declare that other source data supporting the findings of this study within the article and its Supplementary information files are available upon reasonable request to the corresponding author. Source data are provided with this paper.

## Source data

Source Data
