# Supplementary material for: Limited survival and impaired hepatic fasting metabolism in mice with constitutive Rag GTPase signaling
Source: Nat Commun. 2021 Jun 16;12:3660. doi: 10.1038/s41467-021-23857-8 (PMC8209044; doi:10.1038/s41467-021-23857-8)
Supplement: Supplementary file 1 — Supplementary Information [file 41467_2021_23857_MOESM1_ESM.pdf]

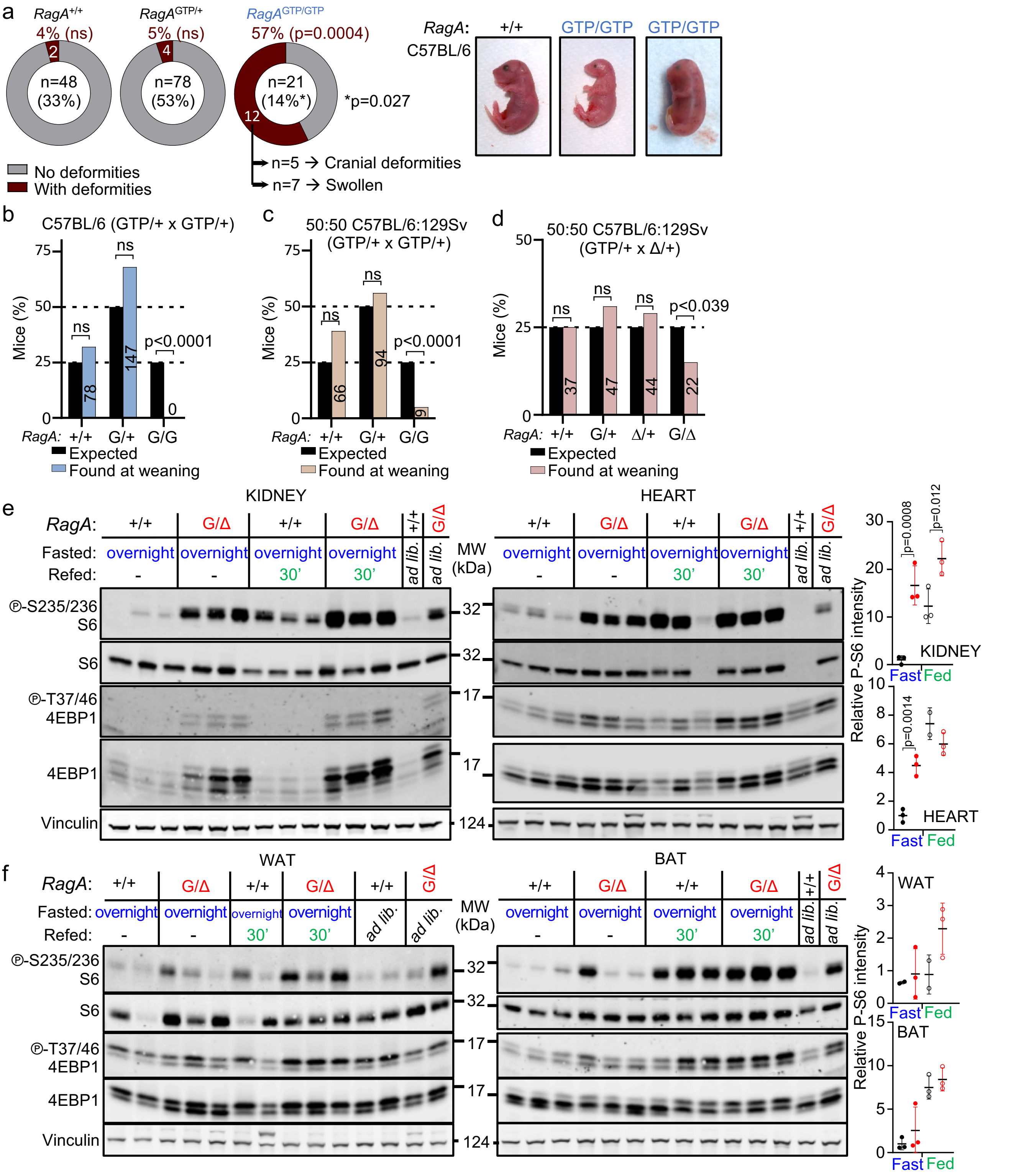
a
RagA+/+
4% (ns)
2
n=48
(33%)
RagAGTP/+
5% (ns)
4
n=78
(53%)
RagAGTP/GTP
57% (p=0.0004)
n=21
(14%*)
12
*p=0.027
n=5 → Cranial deformities
n=7 → Swollen
No deformities
With deformities
RagA:
+/+
GTP/GTP
GTP/GTP
C57BL/6
b
C57BL/6 (GTP/+ x GTP/+)
Mice (%)
ns
ns
p<0.0001
78
147
0
RagA: +/+ G/+ G/G
Expected
Found at weaning
c
50:50 C57BL/6:129Sv
(GTP/+ x GTP/+)
Mice (%)
ns
ns
p<0.0001
66
94
6
RagA: +/+ G/+ G/G
Expected
Found at weaning
d
50:50 C57BL/6:129Sv
(GTP/+ x Δ/+)
Mice (%)
ns
ns
ns
p<0.039
37
47
44
22
RagA: +/+ G/+ Δ/+ G/Δ
Expected
Found at weaning
e
KIDNEY
HEART
RagA:
+/+
G/Δ
Fasted:
overnight
Refed:
30'
ad lib.
MW (kDa)
Ⓟ-S235/236 S6
S6
Ⓟ-T37/46 4EBP1
4EBP1
Vinculin
32
17
124
p=0.0008
p=0.012
p=0.0014
Relative P-S6 intensity
KIDNEY
HEART
Fast
Fed
f
WAT
BAT
RagA:
+/+
G/Δ
Fasted:
overnight
Refed:
30'
ad lib.
MW (kDa)
Ⓟ-S235/236 S6
S6
Ⓟ-T37/46 4EBP1
4EBP1
Vinculin
32
17
124
Relative P-S6 intensity
WAT
BAT
Fast
Fed

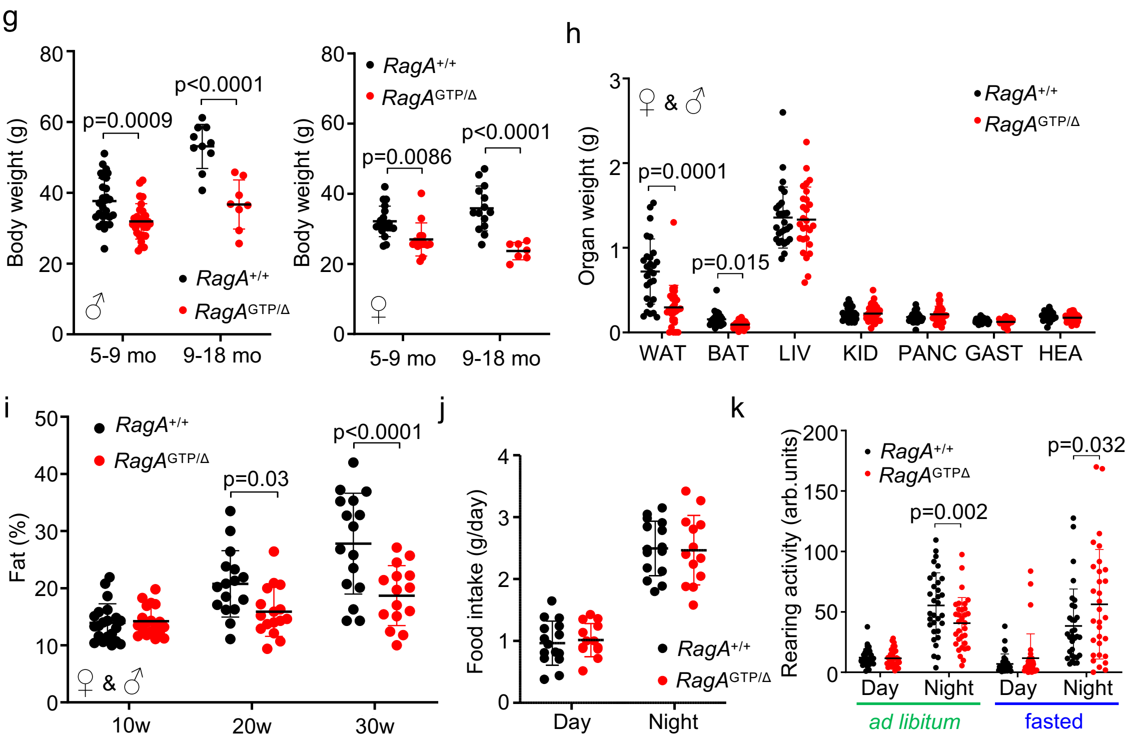

Supplementary Fig. 1 | Related to Figure 1. a, For pups at E19.5 the embryos were removed from the mum by c-section at E19.5 and deformities and mendelian ratios were evaluated (left). Representative pictures of RagA C57BL/6 neonates at E19.5 (right). b, $RagA^{GTP/+}$ mice were intercrossed and the mendelian ratio of the progeny was calculated at weaning. c, 50:50 C57BL/6:129Sv mixed background $RagA^{GTP/+}$ mice were intercrossed and the mendelian ratio of the progeny was calculated at weaning. d, 50:50 C57BL/6:129Sv mixed background $RagA^{GTP/+}$ and $RagA^{\Delta/+}$ mice were intercrossed and the mendelian ratio of the progeny was calculated at weaning. e, 29- to 34-week-old $RagA^{+/+}$ and $RagA^{GTP/\Delta}$ males were deprived from food for 16h and sacrificed following a 30 min refeeding (n=3). *Ad libitum (Ad lib.)* fed mice were also included (n=1). Protein lysates from the kidney and the heart were immunoblotted for the indicated proteins (left) and the quantification of P-S235/236-S6 intensity relative to vinculin was calculated (right). Fast: Fasted. f, 25- to 36-week-old $RagA^{+/+}$ and $RagA^{GTP/\Delta}$ females were deprived from food for 16h and sacrificed following a 30 min refeeding . *Ad libitum* fed mice were also included. Protein lysates from the white and brown adipose tissue (WAT & BAT) were immunoblotted for the indicated proteins (left) and the quantification of P-S235/236-S6 intensity relative to vinculin was calculated (right). Fast: Fasted. WAT ($RagA^{+/+}$ n=2 and $RagA^{GTP/\Delta}$ n=3 fasted/refed and n=2 *ad lib.*) BAT (n=3 fasted/refed and n=1 *ad lib.)*. g, Body weight of $RagA^{+/+}$ and $RagA^{GTP/\Delta}$ males and females at indicated times. $RagA^{+/+}$ males (5-9 mo n=30; 9-18 mo n=11) and females (5-9mo n=19; 9-18mo n=14). $RagA^{GTP/\Delta}$ males (5-9 mo n=28; 9-18 mo n=8) and females (5-9 mo n=14; 9-18 mo n=7). mo: months. h, Weight of the indicated organs. White adipose tissue (WAT), brown adipose tissue (BAT), Liver (LIV), Kidney (KID), pancreas (PANC), gastrocnemius muscle (GAST), heart (HEA). $RagA^{+/+}$ (WAT, LIV, KID, PANC n=27; BAT, HE n=25; GAST n=23) and $RagA^{GTP/\Delta}$ (WAT, LIV, KID, PANC, HEA n=27; BAT n=25, GAST n=24). Statistical significance was calculated by using unpaired two-tailed t-test corrected for multiple comparisons (Holm-Sidak). i, The percentage of fat was obtained from body composition analysis in $RagA^{+/+}$ (10w n=21; 20w n=16; 30w n=16) and $RagA^{GTP/\Delta}$ (10w n=19; 20w n=16; 30w n=15) mice at indicated times. j, Food intake during day and night in 6- to 11-week-old $RagA^{+/+}$ (n=15) and $RagA^{GTP/\Delta}$ (n=13) males and females. Data is the average value of 3 days and 3 nights. k, Rearing during day and night in 10- to 30-week-old $RagA^{+/+}$ (n=32) and $RagA^{GTP/\Delta}$ (n=32) males and females in *ad libitum* fed or fasted conditions. Data is the average value of 3 days and 3 nights in *ad libitum* conditions and 1 day and night under fasting. From a to b, statistical significance was calculated by using Chi-square test. For e-g, i, and k statistical significance was calculated by using 2-way ANOVA with Sidak's multiple comparison correction. In all panels, horizontal lines indicate the mean and error bars depict Standard deviation (SD).

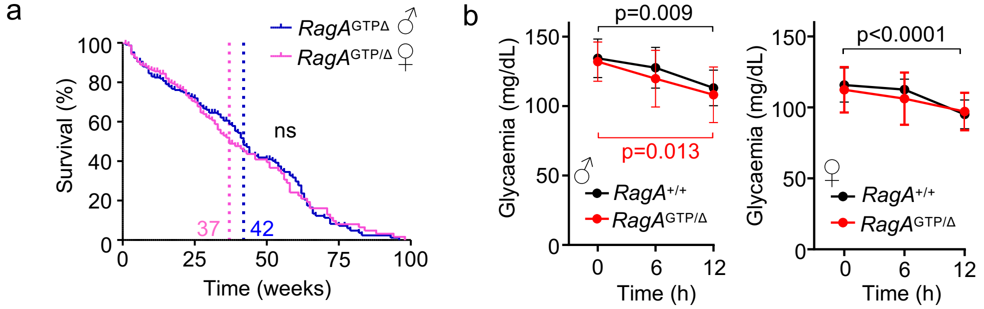

Supplementary Fig. 2 | Related to Figure 2. a. Kaplan–Meier survival curves of *RagA*$^{GTP/\Delta}$ males (n=235) and females (n=220). Mean survival annotated in pink (females) and blue (males). Statistical significance was calculated with the log-rank (Mantel-Cox) test. b. 22- to 33-week-old *RagA*$^{+/+}$ (males n=8; females n=10) and *RagA*$^{GTP/\Delta}$ (males n=8; females n=6) mice were fasted from 7:30am to 7:30pm. Glucose was measured at indicated times. Statistical significance was calculated by using 2-way ANOVA with Sidak's multiple comparison correction. In all panels, horizontal lines indicate the mean and error bars depict Standard deviation (SD).

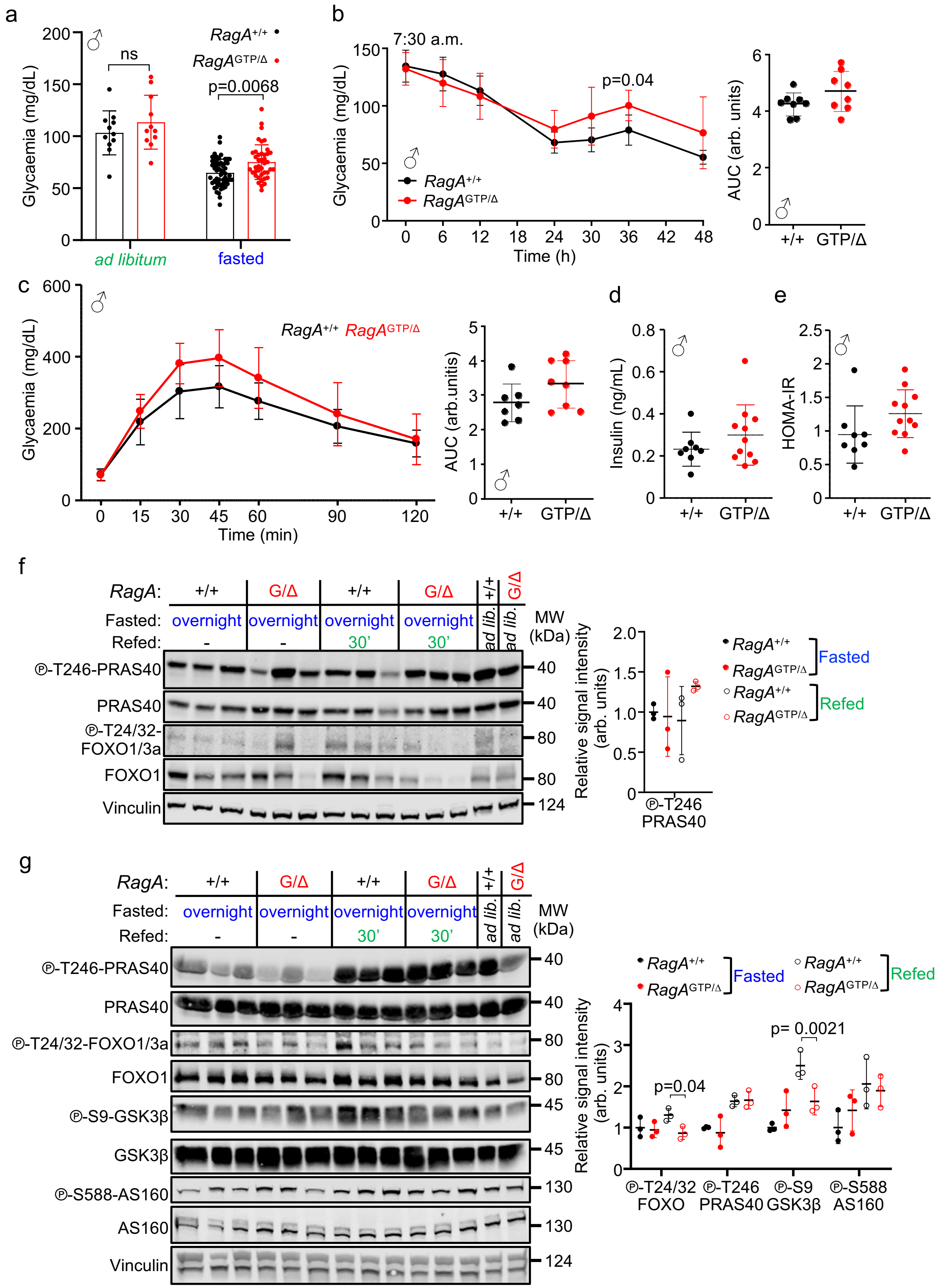
a
ns
p=0.0068
Glycaemia (mg/dL)
RagA+/+
RagAGTP/Δ
ad libitum
fasted
b
7:30 a.m.
p=0.04
Glycaemia (mg/dL)
Time (h)
AUC (arb. units)
+/+
GTP/Δ
c
Glycaemia (mg/dL)
Time (min)
AUC (arb.units)
d
Insulin (ng/mL)
e
HOMA-IR
f
RagA:
G/Δ
Fasted:
overnight
Refed:
30'
ad lib.
MW (kDa)
Ⓟ-T246-PRAS40
PRAS40
Ⓟ-T24/32-FOXO1/3a
FOXO1
Vinculin
Relative signal intensity (arb. units)
Ⓟ-T246 PRAS40
Fasted
Refed
g
Ⓟ-S9-GSK3β
GSK3β
Ⓟ-S588-AS160
AS160
p=0.04
p= 0.0021
Ⓟ-T24/32 FOXO
Ⓟ-T246 PRAS40
Ⓟ-S9 GSK3β
Ⓟ-S588 AS160

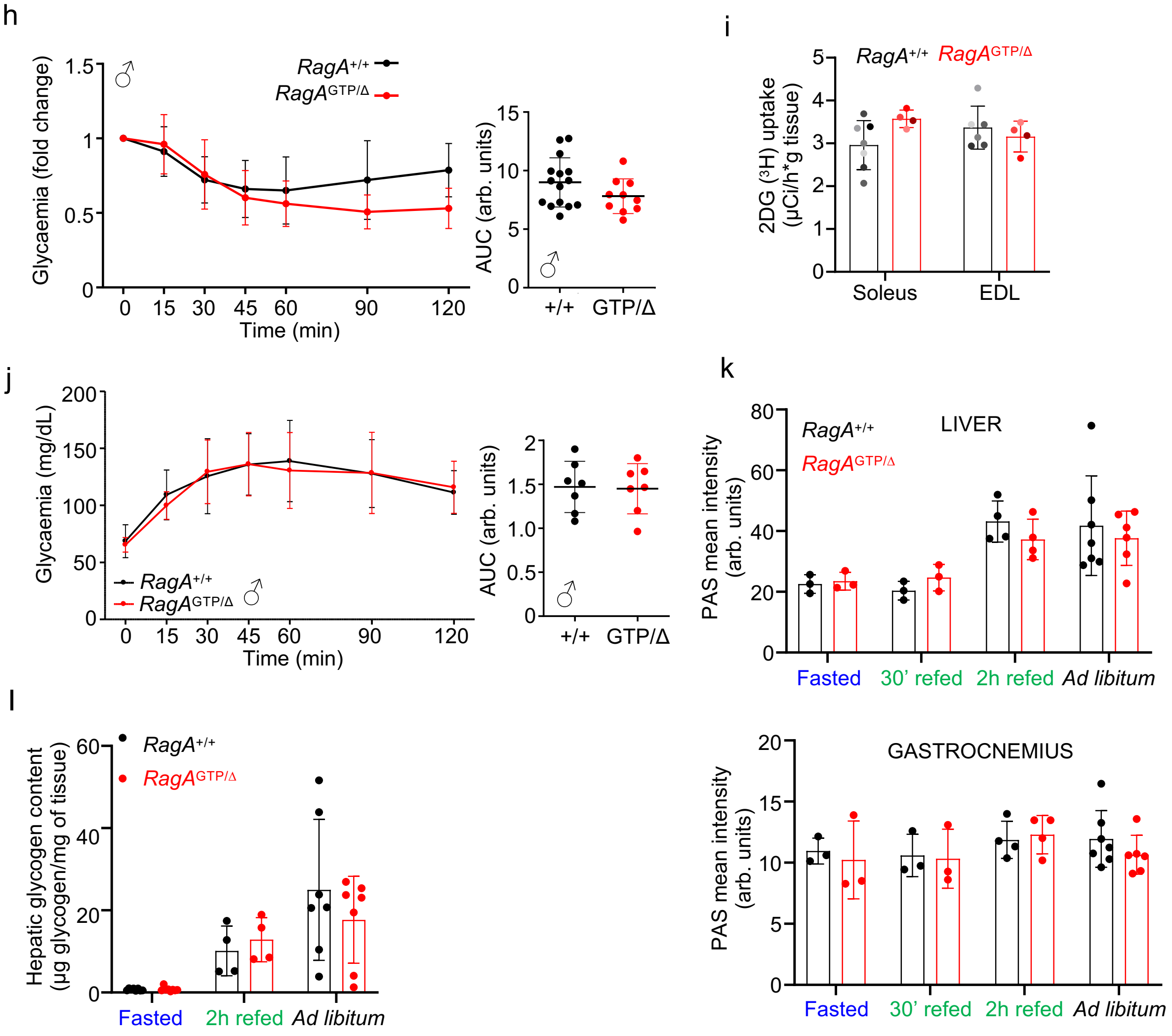

Supplementary Fig. 3 | Related to Figure 3 a, Glycaemia of 5- to 31-week-old *RagA*$^{+/+}$ and *RagA*$^{GTP/\Delta}$ males in *ad libitum* conditions and after 16h of fasting. *RagA*$^{+/+}$ (*ad libitum* n=11 and fasted n=55). *RagA*$^{GTP/\Delta}$ (*ad libitum* n=11 and fasted n=41). Statistical significance was calculated by using 2-way ANOVA with Sidak's multiple comparison correction. b, 22- to 27-week-old *RagA*$^{+/+}$ and *RagA*$^{GTP/\Delta}$ mice were starved for 48h. Glucose was monitored every 6h except in between 8pm and 8am. (n=8). c, Glucose tolerance test (GTT) of 19- to 24-week-old *RagA*$^{+/+}$ (n=7) and *RagA*$^{GTP/\Delta}$ (n=7) male mice. For b and c statistical significance was calculated by using 2-way ANOVA with Sidak's multiple comparison correction. The area under the curve (AUC) was calculated and statistical significance was calculated by using unpaired two-tailed t-test. d, Insulin levels of 24- to 34-week-old *RagA*$^{+/+}$ (n=8) and *RagA*$^{GTP/\Delta}$ (n=11) male mice fasted 16h. e, HOMA-IR ratio calculated from the ratio between insulin and glucose levels obtained from 24- to 34-week-old *RagA*$^{+/+}$ (n=8) and *RagA*$^{GTP/\Delta}$ (n=11) male mice fasted for 16h. f, g, 25- to 36-week-old *RagA*$^{+/+}$ and *RagA*$^{GTP/\Delta}$ females were deprived from food for 16h and sacrificed following a 30 min refeeding (n=3). *Ad libitum (Ad lib.)* fed mice were also included (n=1). Protein lysates from the liver (f) and gastrocnemius muscle (g) were immunoblotted for the indicated proteins (left) and the quantification of the intensity relative to vinculin was calculated for the indicated antibodies (right). Statistical significance was calculated by using 2-way ANOVA with Sidak's multiple comparison correction. h, Insulin tolerance test (ITT) of 20- to 35-week-old *RagA*$^{+/+}$ (n=15) and *RagA*$^{GTP/\Delta}$ (n=10) males. The area under the curve (AUC) was calculated. i, 16- to 20-week-old *RagA*$^{+/+}$ (n=7) and *RagA*$^{GTP/\Delta}$ (n=4) males were deprived from food for 16h. The ex-vivo glucose uptake from soleus and extensor digitorum longus (EDL) muscles was investigated and represented relative to the muscle weight. Each coloured dot indicate paired muscles from the same animal. 2DG: 2-Deoxy-D-glucose. j, Pyruvate tolerance test (PTT) of 17- to 18-week-old RagA$^{+/+}$ and *RagA*$^{GTP/\Delta}$ (n=7) males. The area under the curve (AUC) was calculated. k, Quantification of the median intensity of the periodic acid–Schiff (PAS) staining in the liver and gastrocnemius muscle of 15- to 36-week-old *RagA*$^{+/+}$ and *RagA*$^{GTP/\Delta}$ females at indicated conditions. Fasted (n=3), 30' refed (n=3), 2h refed (n=4), *ad libitum* (*RagA*$^{+/+}$ n=7 and *RagA*$^{GTP/\Delta}$ n=6). l, Biochemical determination of glycogen content in the liver of 15- to 36-week-old *RagA*$^{+/+}$ and *RagA*$^{GTP/\Delta}$ females at indicated conditions fasted (n=8), 2h refed (n=4), *ad libitum* (n=7). In all panels, horizontal lines indicate the mean and error bars depict Standard deviation (SD).

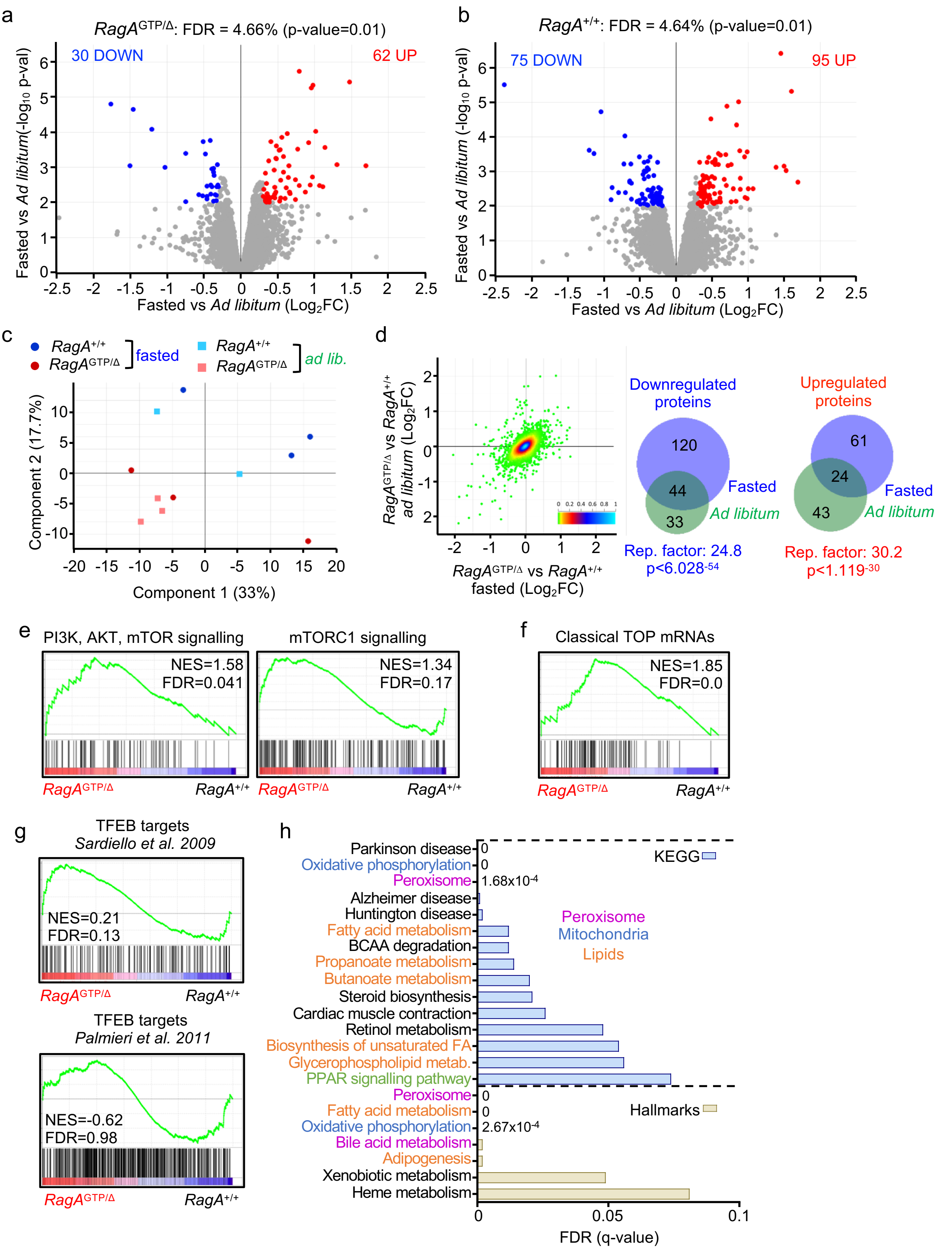
a
RagA^GTP/Δ: FDR = 4.66% (p-value=0.01)
30 DOWN
62 UP
Fasted vs Ad libitum(-log10 p-val)
Fasted vs Ad libitum (Log2FC)
b
RagA+/+: FDR = 4.64% (p-value=0.01)
75 DOWN
95 UP
Fasted vs Ad libitum (-log10 p-val)
Fasted vs Ad libitum (Log2FC)
c
RagA+/+
RagA^GTP/Δ
fasted
RagA+/+
RagA^GTP/Δ
ad lib.
Component 2 (17.7%)
Component 1 (33%)
d
RagA^GTP/Δ vs RagA+/+ ad libitum (Log2FC)
RagA^GTP/Δ vs RagA+/+ fasted (Log2FC)
Downregulated proteins
120
44
33
Fasted
Ad libitum
Rep. factor: 24.8
p<6.028-54
Upregulated proteins
61
24
43
Fasted
Ad libitum
Rep. factor: 30.2
p<1.119-30
e
PI3K, AKT, mTOR signalling
NES=1.58
FDR=0.041
mTORC1 signalling
NES=1.34
FDR=0.17
RagA^GTP/Δ
RagA+/+
f
Classical TOP mRNAs
NES=1.85
FDR=0.0
g
TFEB targets
Sardiello et al. 2009
NES=0.21
FDR=0.13
TFEB targets
Palmieri et al. 2011
NES=-0.62
FDR=0.98
h
KEGG
Parkinson disease 0
Oxidative phosphorylation 0
Peroxisome 1.68x10-4
Alzheimer disease
Huntington disease
Fatty acid metabolism
BCAA degradation
Propanoate metabolism
Butanoate metabolism
Steroid biosynthesis
Cardiac muscle contraction
Retinol metabolism
Biosynthesis of unsaturated FA
Glycerophospholipid metab.
PPAR signalling pathway
Peroxisome
Mitochondria
Lipids
Hallmarks
Peroxisome 0
Fatty acid metabolism 0
Oxidative phosphorylation 2.67x10-4
Bile acid metabolism
Adipogenesis
Xenobiotic metabolism
Heme metabolism
FDR (q-value)

Supplementary Fig. 4 | Related to Figure 4. a, Volcano plot highlighting (in red and blue) proteins with significantly different levels in fasted *RagA*$^{GTP/\Delta}$ (n=3) vs *ad libitum* fed *RagA*$^{GTP/\Delta}$ (n=3) livers. b, Volcano plot highlighting (in red and blue) proteins with significantly different levels in fasted *RagA*$^{+/+}$ (n=3) vs *ad libitum RagA*$^{+/+}$ (n=2) livers. Statistical significance for sections a and b was calculated using the LIMMA program as indicated in Materials and Methods. c, Principal Component Analysis (PCA) of proteomic profiles from *RagA*$^{+/+}$ (fasted n=3 and *ad libitum* fed n=2) and *RagA*$^{GTP/\Delta}$ (fasted n=3 and *ad libitum* fed n=3) livers. Each dot represents an individual mouse. d, Correlation diagram (density plot) comparing the significantly different proteins between genotypes in both fasted and *ad libitum* states (left). Proportional Venn diagram showing the overlap of significantly up-regulated (red) or down-regulated (blue) proteins between both nutritional states (right). Rep. factor: Representation factor. e-g, Enrichment of gene sets (GSEA) related to indicated hallmark signatures (e), related to classical TOP mRNAs signature (f), or related to indicated TFEB signatures (g) in livers from fasted *RagA*$^{GTP/\Delta}$ (n=3) vs *RagA*$^{+/+}$ (n=3) mice. NES: normalized enrichment score; FDR: false discovery rate. h, Graphical representation of the false discover rates (FDRs) from the indicated KEGG and Hallmark gene sets.

Supp. Figure 5

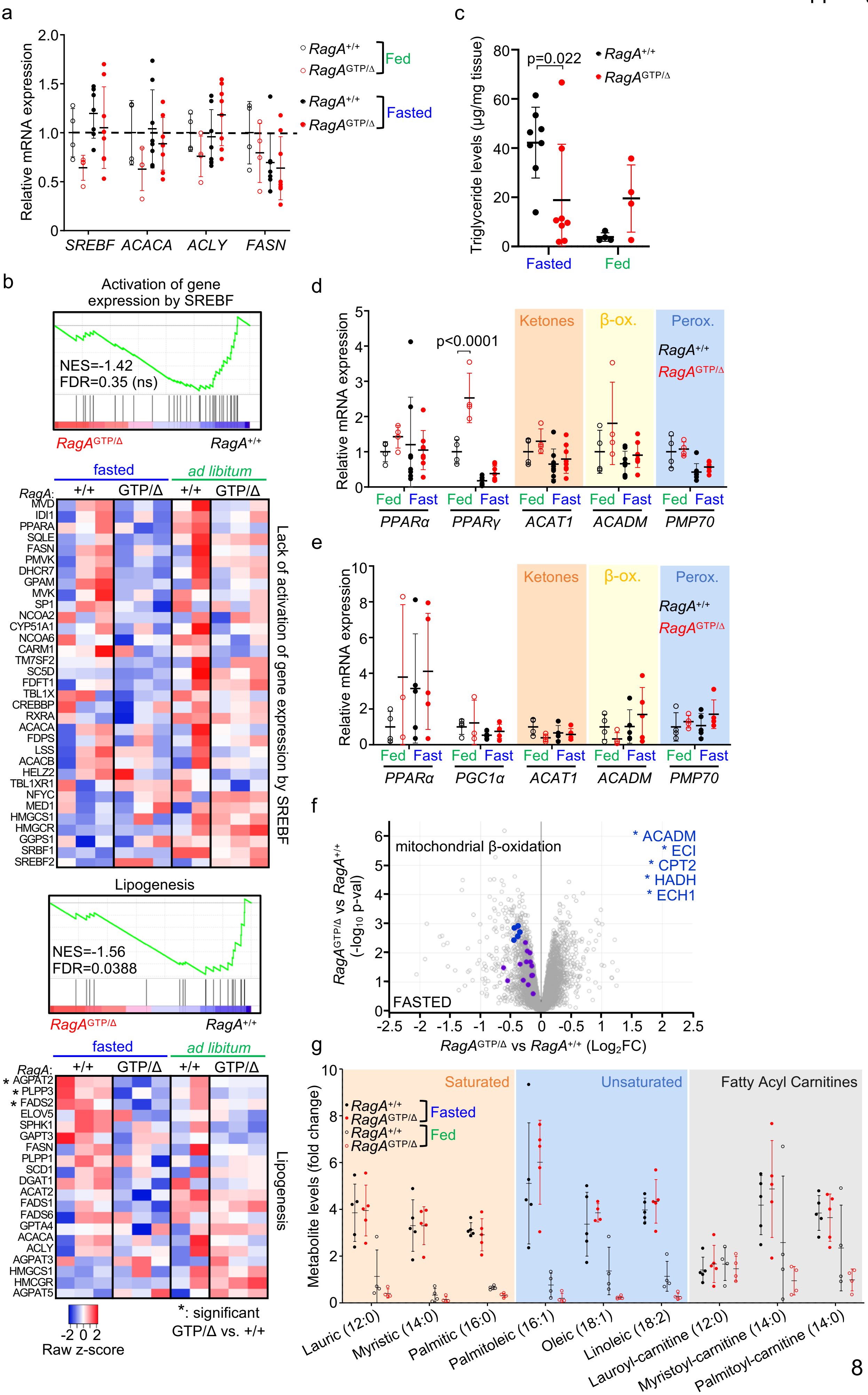

Supp. Figure 5
(cont.)

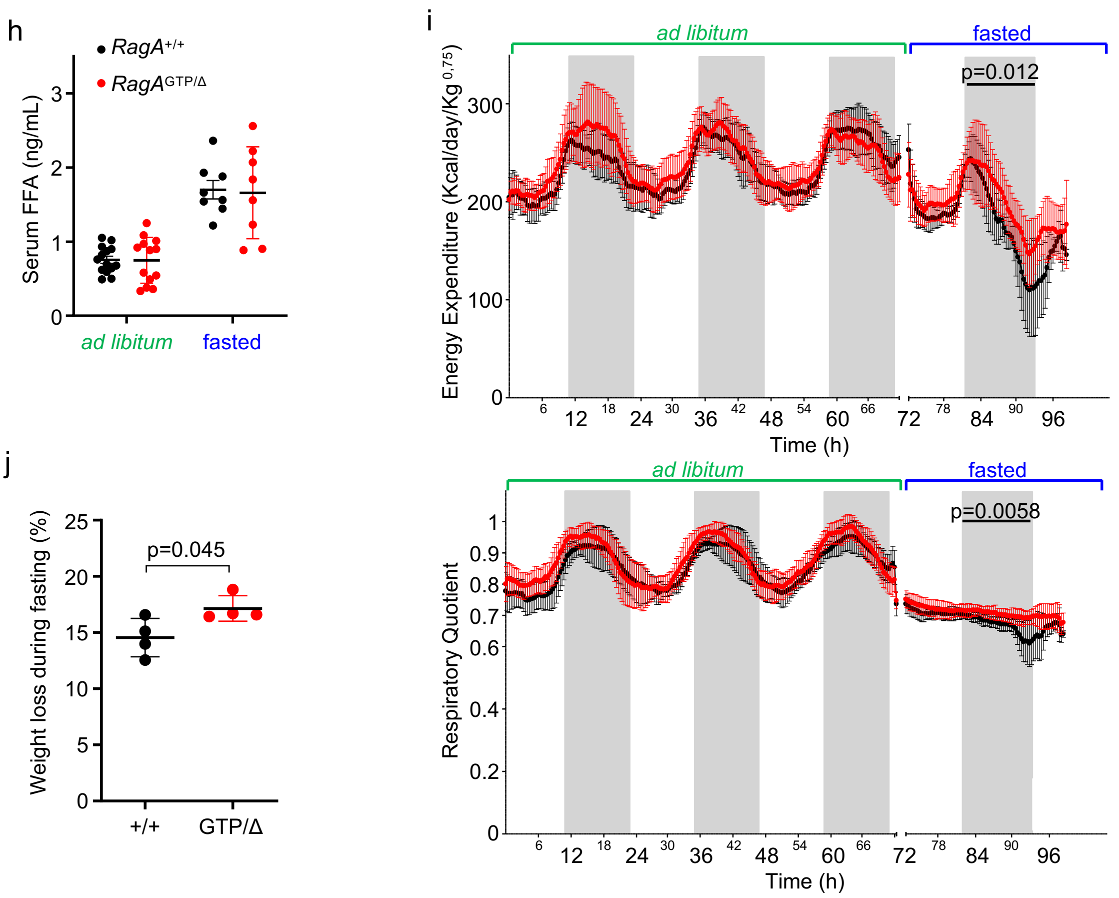

Supplementary Fig. 5 | Related to Figure 5. a, RT-qPCR of livers from 15- to 36-week-old *RagA*$^{+/+}$ (fasted n=8; fed n=4) and *RagA*$^{GTP/\Delta}$ (fasted n=8; fed n=4) females. Expression levels of the indicated genes relative to the average level in fed *RagA*$^{+/+}$ mice. β-actin was used as housekeeping gene. b, Heat map and enrichment of gene sets (GSEA) related to proteins involved in *SREBF* gene expression (top) and lipogenesis (bottom) detected in the proteomics experiment from Figure 4. *RagA*$^{+/+}$ (fasted n=3; *ad libitum* fed n=2) and *RagA*$^{GTP/\Delta}$ (fasted n=3; *ad libitum* fed n=3). Statistical significance was calculated using the LIMMA program as indicated in Materials and Methods. The * indicates significant difference between *RagA*$^{+/+}$ and *RagA*$^{GTP/\Delta}$ in fasted conditions. NES: normalized enrichment score; FDR: false discovery rate. c, Hepatic triglyceride levels of 15- to 36-week-old *RagA*$^{+/+}$ (fasted n=8; fed n=4) and *RagA*$^{GTP/\Delta}$ (fasted n=8; fed n=4) females. d and e, RT-qPCR of white adipose depots (d) and gastrocnemius muscle (e) of 15- to 36-week-old *RagA*$^{+/+}$ (fasted n=8; refed n=4) and *RagA*$^{GTP/\Delta}$ (fasted n=8; refed n=4) females. The expression levels of the indicated genes, relative to those of β-actin were relativized to the average level in of *RagA*$^{+/+}$ mice in fed state. Fast: Fasted. f, Volcano plot with colored dots highlighting the distribution of proteins involved in mitochondrial β-oxidation (comparison: fasted *RagA*$^{GTP/\Delta}$ n=3; vs. fasted *RagA*$^{+/+}$ n=3). Purple dots indicate the proteins whose levels have no statistically significant changes; blue dots and labels indicate proteins levels with statistically significant changes. Statistical significance was calculated using the LIMMA program as indicated in Materials and Methods. g, 15- to 19-week-old *RagA*$^{+/+}$ and *RagA*$^{GTP/\Delta}$ females were fasted for 16h, then a part of them were refed for 2h (fed state). Mice were bled when sacrificed. Serum was collected and the levels of indicated metabolites were analyzed by targeted LC-MS. *RagA*$^{+/+}$ and *RagA*$^{GTP/\Delta}$ (fasted n=5; refed n=4). h, The levels of circulating free fatty acids were measured in 15- to 36-week-old *RagA*$^{+/+}$ (*ad libitum* n=14; fasted n=8) and *RagA*$^{GTP/\Delta}$ (*ad libitum* n=14; fasted n=8) females. i. Graphical representation of Energy Expenditure (EE) and Respiratory Quotient (RQ) of 15- to 19-week-old *RagA*$^{+/+}$ (n=8) and *RagA*$^{GTP/\Delta}$ (n=7) females. Mice were monitored in metabolic cages for 3 days and nights in *ad libitum* conditions and for 27h in fasting. The quantification and the statistics were done by representing the average value of 3 days and 3 nights in *ad libitum* conditions and 1 day and night for fasted conditions. j, 15- to 19-week-old *RagA*$^{+/+}$ (n=4) and *RagA*$^{GTP/\Delta}$ (n=4) females were fasted for 27h. Body weight was measured before and after fasting and the percentage of body weight loss was calculated. Statistical significance was calculated by using unpaired two-tailed t-test. For, c-e, g and i, statistical significance was calculated by using 2-way ANOVA with Sidak's multiple comparison correction. In all panels, horizontal lines indicate the mean and error bars depict Standard deviation (SD).

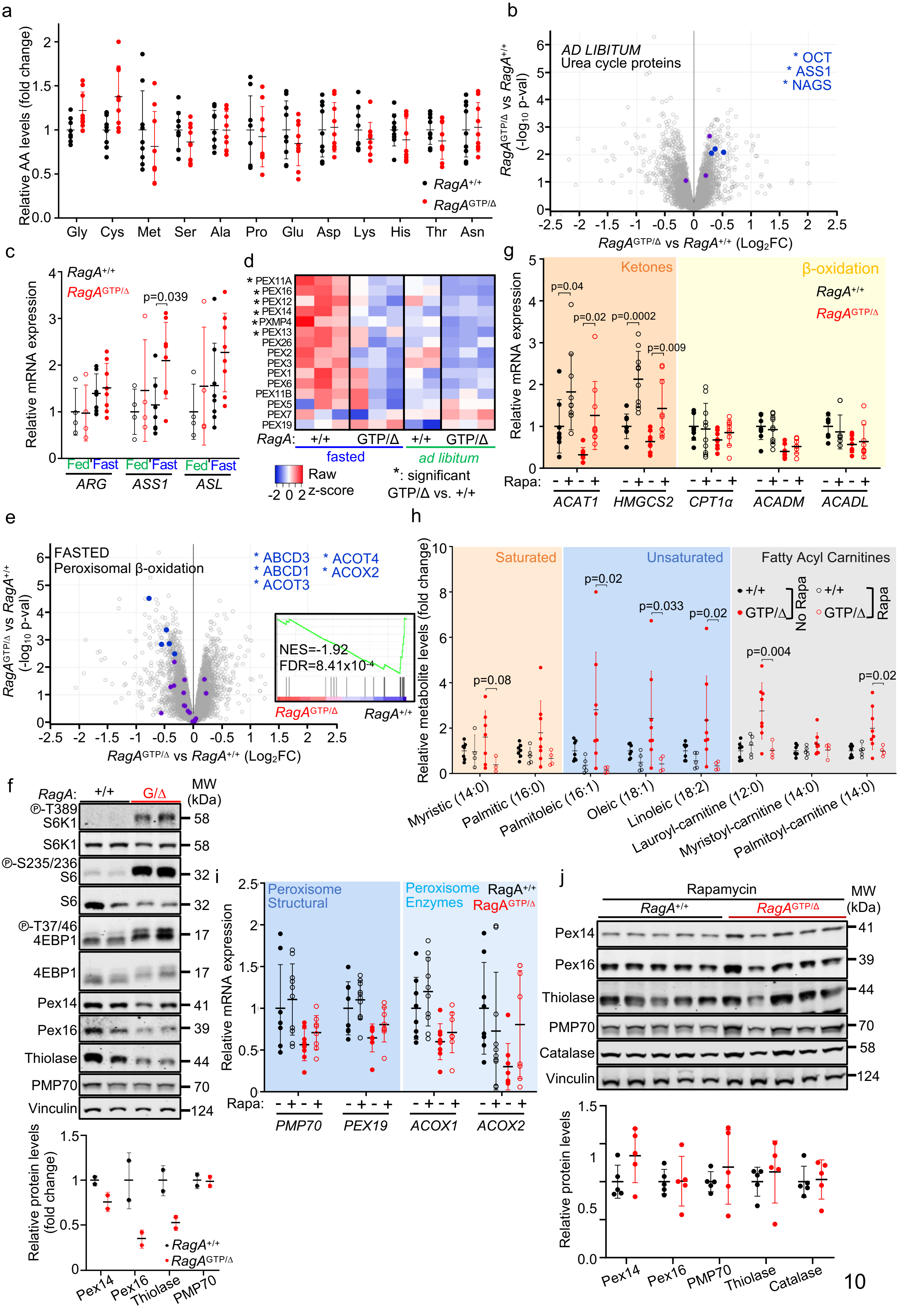
a
Relative AA levels (fold change)
Gly Cys Met Ser Ala Pro Glu Asp Lys His Thr Asn
RagA+/+
RagAGTP/Δ
b
AD LIBITUM
Urea cycle proteins
* OCT
* ASS1
* NAGS
RagAGTP/Δ vs RagA+/+ (-log10 p-val)
RagAGTP/Δ vs RagA+/+ (Log2FC)
c
RagA+/+
RagAGTP/Δ
p=0.039
Relative mRNA expression
Fed Fast Fed Fast Fed Fast
ARG ASS1 ASL
d
* PEX11A
* PEX16
* PEX12
* PEX14
* PXMP4
* PEX13
PEX26
PEX2
PEX3
PEX1
PEX6
PEX11B
PEX5
PEX7
PEX19
RagA: +/+ GTP/Δ +/+ GTP/Δ
fasted
ad libitum
Raw z-score
-2 0 2
*: significant GTP/Δ vs. +/+
g
Ketones
β-oxidation
RagA+/+
RagAGTP/Δ
p=0.04
p=0.02
p=0.0002
p=0.009
Relative mRNA expression
Rapa: - + - +
ACAT1 HMGCS2 CPT1α ACADM ACADL
e
FASTED
Peroxisomal β-oxidation
* ABCD3
* ABCD1
* ACOT3
* ACOT4
* ACOX2
NES=-1.92
FDR=8.41x10-4
RagAGTP/Δ
RagA+/+
RagAGTP/Δ vs RagA+/+ (-log10 p-val)
RagAGTP/Δ vs RagA+/+ (Log2FC)
h
Saturated
Unsaturated
Fatty Acyl Carnitines
+/+
GTP/Δ
No Rapa
Rapa
p=0.08
p=0.02
p=0.033
p=0.02
p=0.004
p=0.02
Relative metabolite levels (fold change)
Myristic (14:0)
Palmitic (16:0)
Palmitoleic (16:1)
Oleic (18:1)
Linoleic (18:2)
Lauroyl-carnitine (12:0)
Myristoyl-carnitine (14:0)
Palmitoyl-carnitine (14:0)
f
RagA: +/+ G/Δ
MW (kDa)
Ⓟ-T389 S6K1
S6K1
Ⓟ-S235/236 S6
S6
Ⓟ-T37/46 4EBP1
4EBP1
Pex14
Pex16
Thiolase
PMP70
Vinculin
Relative protein levels (fold change)
RagA+/+
RagAGTP/Δ
Pex14 Pex16 Thiolase PMP70
i
Peroxisome Structural
Peroxisome Enzymes
RagA+/+
RagAGTP/Δ
Relative mRNA expression
Rapa: - + - +
PMP70 PEX19 ACOX1 ACOX2
j
Rapamycin
RagA+/+
RagAGTP/Δ
MW (kDa)
Pex14
Pex16
Thiolase
PMP70
Catalase
Vinculin
Relative protein levels
Pex14 Pex16 PMP70 Thiolase Catalase

Supplementary Fig. 6 | Related to Figure 6. a, The indicated amino acid levels were measured by targeted LC-MS in serum from 15- to 19-week-old *ad libitum* *RagA*$^{+/+}$ (n=9) and *RagA*$^{GTP/\Delta}$ (n=9) females (other significant changes in amino acid levels are presented in Figure 6b). b, Volcano plot with colored dots highlighting the distribution of proteins from Figure 4 involved in the urea cycle (comparison: *ad libitum RagA*$^{GTP/\Delta}$ n=3; vs *ad libitum RagA*$^{+/+}$ n=2). Purple dots indicate proteins levels that do not change significantly; blue dots and labels indicate significant changes in protein levels. Statistical significance was calculated using the LIMMA program as described in Materials and Methods. c, RT-qPCR of livers from 15- to 36-week-old *RagA*$^{+/+}$ (fasted n=8; fed n=4) and *RagA*$^{GTP/\Delta}$ (fasted n=8; fed n=4) females. Expression levels of the indicated genes relative to those of β-actin were relativized to the average level in of *RagA*$^{+/+}$ mice in fed state. Fast: Fasted. d, Heat map of all peroxin proteins detected in the proteomics analysis from Figure 4. *RagA*$^{+/+}$ (fasted n=3; *ad libitum* fed n=2) and *RagA*$^{GTP/\Delta}$ (fasted n=3, *ad libitum* n=3). Statistical significance was calculated using the LIMMA program as indicated in Materials and Methods. The * indicates a statistically significant difference between *RagA*$^{+/+}$ and *RagA*$^{GTP/\Delta}$ mice in fasted conditions. e, Volcano plot (left) with the distribution of proteins from Figure 4 involved in peroxisomal β-oxidation in color (comparison: fasted *RagA*$^{GTP/\Delta}$ n=3; vs. fasted *RagA*$^{+/+}$ n=3). Purple dots indicate non-significant differences in protein levels; blue dots and labels indicate significant changes in protein levels. Statistical significance was calculated using the LIMMA program as indicated in Materials and Methods. Enrichment of gene sets (GSEA) related to peroxisomal β-oxidation in fasted livers of *RagA*$^{GTP/\Delta}$ (n=3) and *RagA*$^{+/+}$ (n=3) mice (right). NES: normalized enrichment score; FDR: false discovery rate. f, *RagA*$^{+/+}$ and *RagA*$^{GTP/\Delta}$ primary hepatocytes were cultured DMEM:F12 in media without amino acids for 16h, then protein extracts were collected and indicated proteins were immunoblotted (top). For quantification (bottom), proteins were normalized first to vinculin levels, and then to the levels of *RagA*$^{+/+}$ mice. (n=2). g, 11- to 21-week-old *RagA*$^{+/+}$ and *RagA*$^{GTP/\Delta}$ females were treated with Rapamycin encapsulated in food for 2 weeks and fasted 16h before sacrifice. mRNA levels of the indicated genes from liver extracts were quantified by RT-qPCR, relative to β-actin levels, and then normalized to the average levels of samples from non-treated *RagA*$^{+/+}$ mice. *RagA*$^{+/+}$ (no rapa n=8; rapa n=10) and *RagA*$^{GTP/\Delta}$ (no rapa n=8 rapa n=9). h, 16- to 21-week-old *RagA*$^{+/+}$ and *RagA*$^{GTP/\Delta}$ mice were treated with Rapamycin for 2 weeks and fasted for 16h. Liver was collected upon sacrifice and the levels of indicated metabolites were measured by targeted LC-MS. Values were resented normalized to the average level of non-treated 15- to 36-week-old *RagA*$^{+/+}$ mice. *RagA*$^{+/+}$ (no rapa n=8; rapa n=5) and *RagA*$^{GTP/\Delta}$ (no rapa n=8 rapa n=4). i, 11- to 21-week-old *RagA*$^{+/+}$ and *RagA*$^{GTP/\Delta}$ females were treated with Rapamycin encapsulated in food for 2 weeks and fasted 16h before sacrifice. mRNA levels of the indicated genes from liver extracts were quantified by RT-qPCR, relative to β-actin levels, and then normalized to the average levels of samples from non-treated *RagA*$^{+/+}$ mice. *RagA*$^{+/+}$ (no rapa n=8; rapa n=10) and *RagA*$^{GTP/\Delta}$ (no rapa n=8 rapa n=9). j, 7- to 10-week-old *RagA*$^{+/+}$ and *RagA*$^{GTP/\Delta}$ males were treated with encapsulated rapamycin in the food for one week, fasted for 16h and sacrificed. Whole-cell protein lysates from liver samples were immunoblotted (top) for the indicated proteins. For quantification (bottom), proteins were normalized first to vinculin levels, and then to the levels of *RagA*$^{+/+}$ mice in each condition. (n=5). For a, c, and g-i, statistical significance was calculated by using 2-way ANOVA with Sidak's multiple comparison correction. In all panels, horizontal lines indicate the mean and error bars depict Standard deviation (SD).

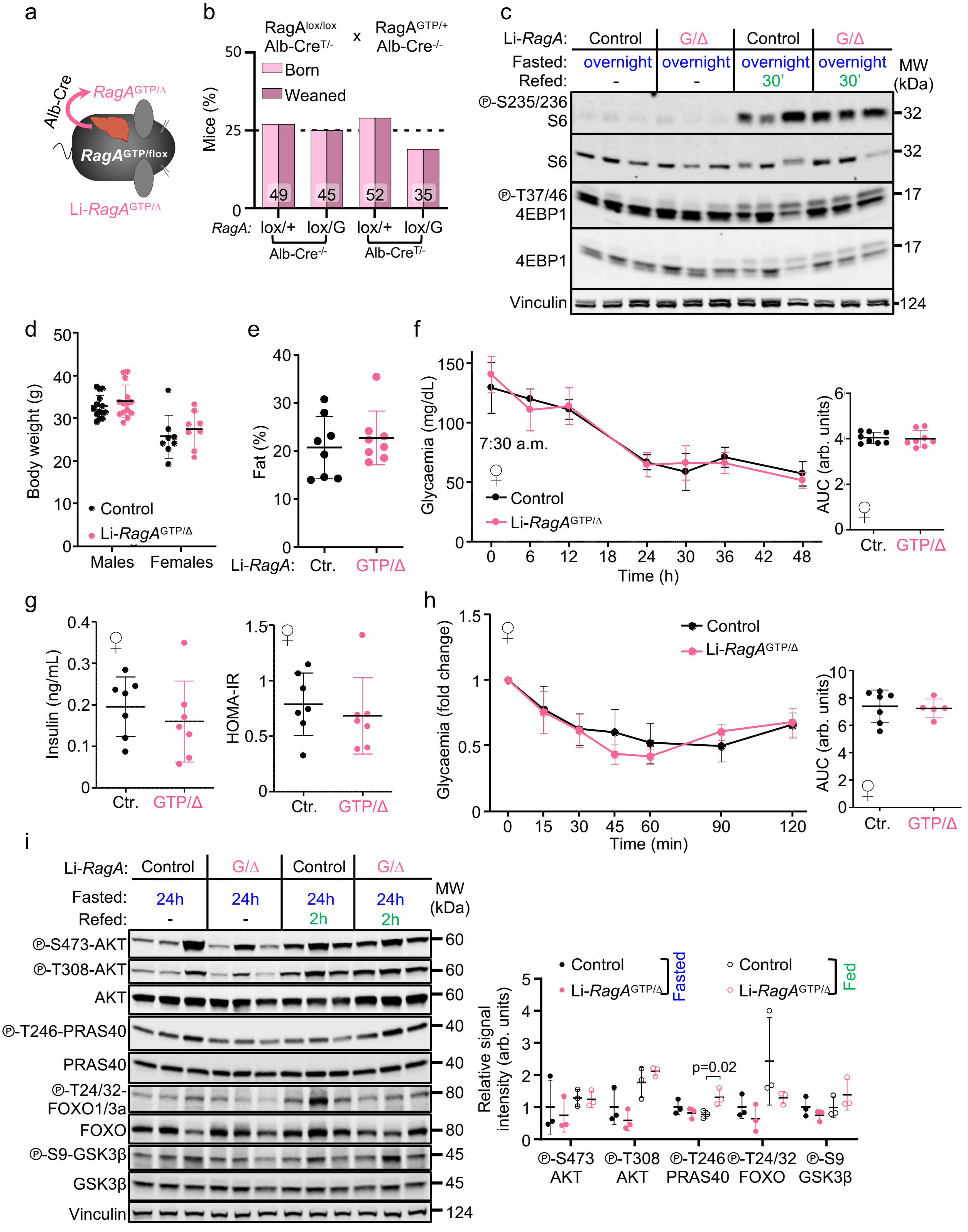
a
Alb-Cre
RagA GTP/Δ
RagA GTP/flox
Li-RagA GTP/Δ
b
RagA lox/lox Alb-Cre T/- x RagA GTP/+ Alb-Cre -/-
Born
Weaned
Mice (%)
49 45 52 35
RagA: lox/+ lox/G lox/+ lox/G
Alb-Cre -/- Alb-Cre T/-
c
Li-RagA: Control G/Δ Control G/Δ
Fasted: overnight overnight overnight overnight
Refed: - - 30' 30'
MW (kDa)
Ⓟ-S235/236 S6
S6
Ⓟ-T37/46 4EBP1
4EBP1
Vinculin
d
Body weight (g)
Control
Li-RagA GTP/Δ
Males Females
e
Fat (%)
Li-RagA: Ctr. GTP/Δ
f
Glycaemia (mg/dL)
7:30 a.m.
Control
Li-RagA GTP/Δ
Time (h)
AUC (arb. units)
Ctr. GTP/Δ
g
Insulin (ng/mL)
Ctr. GTP/Δ
HOMA-IR
Ctr. GTP/Δ
h
Glycaemia (fold change)
Control
Li-RagA GTP/Δ
Time (min)
AUC (arb. units)
Ctr. GTP/Δ
i
Li-RagA: Control G/Δ Control G/Δ
Fasted: 24h 24h 24h 24h
Refed: - - 2h 2h
MW (kDa)
Ⓟ-S473-AKT
Ⓟ-T308-AKT
AKT
Ⓟ-T246-PRAS40
PRAS40
Ⓟ-T24/32-FOXO1/3a
FOXO
Ⓟ-S9-GSK3β
GSK3β
Vinculin
Control
Li-RagA GTP/Δ
Fasted
Fed
Relative signal intensity (arb. units)
p=0.02
Ⓟ-S473 AKT
Ⓟ-T308 AKT
Ⓟ-T246 PRAS40
Ⓟ-T24/32 FOXO
Ⓟ-S9 GSK3β

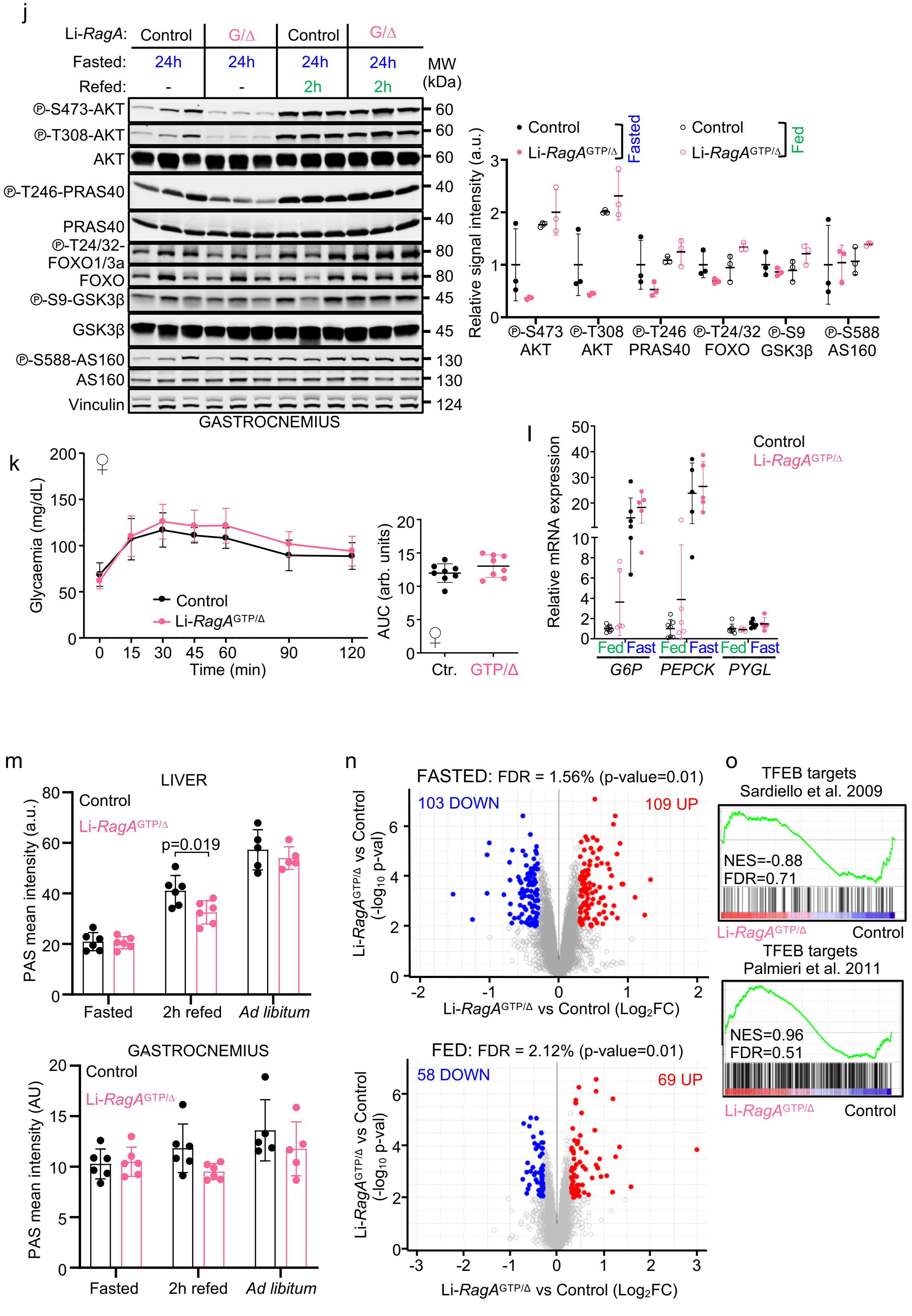
j
Li-RagA: Control G/Δ Control G/Δ
Fasted: 24h 24h 24h 24h
Refed: - - 2h 2h
MW (kDa)
Ⓟ-S473-AKT 60
Ⓟ-T308-AKT 60
AKT 60
Ⓟ-T246-PRAS40 40
PRAS40 40
Ⓟ-T24/32-FOXO1/3a 80
FOXO 80
Ⓟ-S9-GSK3β 45
GSK3β 45
Ⓟ-S588-AS160 130
AS160 130
Vinculin 124
GASTROCNEMIUS
Control
Li-RagA GTP/Δ
Fasted
Fed
Relative signal intensity (a.u.)
Ⓟ-S473 AKT
Ⓟ-T308 AKT
Ⓟ-T246 PRAS40
Ⓟ-T24/32 FOXO
Ⓟ-S9 GSK3β
Ⓟ-S588 AS160
k
Glycaemia (mg/dL)
Control
Li-RagA GTP/Δ
Time (min)
AUC (arb. units)
Ctr.
GTP/Δ
l
Relative mRNA expression
Control
Li-RagA GTP/Δ
Fed Fast Fed Fast Fed Fast
G6P PEPCK PYGL
m
LIVER
Control
Li-RagA GTP/Δ
p=0.019
PAS mean intensity (a.u.)
Fasted
2h refed
Ad libitum
GASTROCNEMIUS
PAS mean intensity (AU)
n
FASTED: FDR = 1.56% (p-value=0.01)
103 DOWN
109 UP
Li-RagA GTP/Δ vs Control (-log10 p-val)
Li-RagA GTP/Δ vs Control (Log2FC)
FED: FDR = 2.12% (p-value=0.01)
58 DOWN
69 UP
o
TFEB targets Sardiello et al. 2009
NES=-0.88
FDR=0.71
TFEB targets Palmieri et al. 2011
NES=0.96
FDR=0.51
Li-RagA GTP/Δ
Control

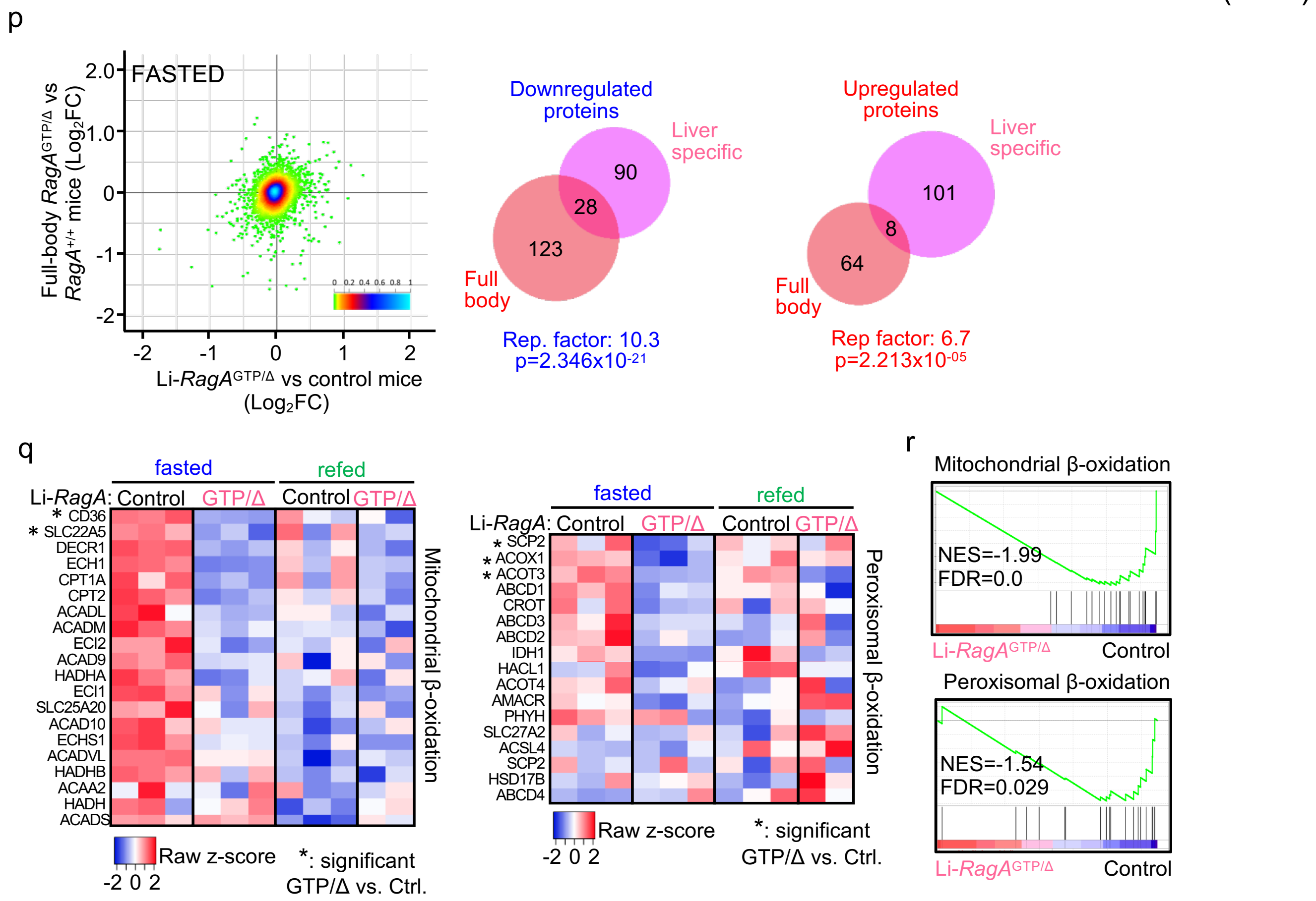

Supplementary Fig. 7 | Related to Figure 7. a, Schematic representation of the liver-specific $RagA^{GTP}$ mouse model. Alb: Allbumin. b, Mendelian ratio of C57BL/6 *RagA*$^{GTP/floxed}$; Alb-Cre$^{T/-}$ mice was calculated at weaning. The dot line (25%) represent the expected Mendelian ratio. c, 6- to 7-week-old control and Li-*RagA*$^{GTP/\Delta}$ males were deprived from food for 16h and sacrificed following a 30 min refeeding. Protein lysates from gastrocnemius muscle were immunoblotted for the indicated proteins. This experiment was repeated with different samples obtaining similar results. d, Body weight of 15- to 27-week-old control and $RagA^{GTP/\Delta}$ males and females. Control males (n=14) and females (n=8). Li-*RagA*$^{GTP/\Delta}$ males (n=14) and females (n=8). e, The percentage of fat was obtained from densitometric analysis in 20- to 26-week-old cntrol (n=8) and Li-*RagA*$^{GTP/\Delta}$ (n=8) males and females. Ctr.: Control. f, 22- to 28-week-old control and Li-*RagA*$^{GTP/\Delta}$ females (n=8) were starved for 48h. Glucose was monitored every 6h except in between 8pm and 8am. The area under the curve (AUC) was calculated. g, Fasting insulin levels of 4- to 6-week-old control and Li-*RagA*$^{GTP/\Delta}$ mice fasted 16h and HOMA-IR ratio calculated from the ratio between insulin and glucose levels obtained from control and Li-*RagA*$^{GTP/\Delta}$ mice fasted for 16h (n=7). h, Insulin tolerance test (ITT) of 30- to 37-week-old control (n=7) and Li-*RagA*$^{GTP/\Delta}$ (n=5) females. The area under the curve (AUC) was calculated. i, j, 13- to 25-week-old control and Li-*RagA*$^{GTP/\Delta}$ females were deprived from food for 24h and sacrificed following a 2h refeeding. Protein lysates from the liver (i) and gastrocnemius muscle (j) were immunoblotted for the indicated proteins and the quantification of the intensity relative to β-actin was calculated for the indicated antibodies. (n=3). k, Pyruvate tolerance test (PTT) of 19- to 24-week-old control (n=8) and Li-*RagA*$^{GTP/\Delta}$ (n=8) females. The area under the curve (AUC) was calculated. l, RT-qPCR of livers from 23- to 42-week-old control (fasted n=6; fed n=7) and Li-*RagA*$^{GTP/\Delta}$ (fasted n=5; fed n=5) females. Expression levels of the indicated genes relative to the average level in fed control mice. β-actin was used as housekeeping gene. Fast: Fasted. m, Quantification of the median intensity of the periodic acid–Schiff (PAS) staining in the liver and gastrocnemius muscle of 15- to 42-week-old control and Li-*RagA*$^{GTP/\Delta}$ males and females at indicated conditions. Fasted (n=6), 2h refed (n=6), *ad libitum* (n=5). n, Volcano plot highlighting (in red and blue) proteins detected in the proteomics analysis of Figure 7c with significantly different levels in fasted Li-*RagA*$^{GTP/\Delta}$ (n=3) vs fasted control (n=3) livers (top). Volcano plot highlighting (in red and blue) proteins with significantly different levels in fed Li-*RagA*$^{GTP/\Delta}$ (n=2) vs fed control (n=3) livers (bottom). Statistical significance was calculated using the LIMMA program as indicated in Materials and Methods. o, Enrichment of gene sets (GSEA) related to indicated TFEB signatures in livers from fasted Li-*RagA*$^{GTP/\Delta}$ (n=3) vs control (n=3) mice from Figure 7c. NES: normalized enrichment score; FDR: false discovery rate. p, Correlation diagram (density plot) comparing the significantly different proteins found in fasted full-body *RagA*$^{GTP/\Delta}$ mice vs *RagA*$^{+/+}$ mice from Figure 4 and fasted Li-*RagA*$^{GTP/\Delta}$ vs control mice from Figure 7c (left). Proportional Venn diagram showing the overlap of significantly up-regulated (red) or down-regulated (blue) proteins between both experiments (right). Rep. factor: Representation factor. Statistical significance was calculated with the nemates tool as indicated in Methods. q, Heat map of proteins related to mitochondrial and peroxisomal β-oxidation detected in the proteomics analysis from Figure 7c. Control (fasted n=3; fed n=3) and Li-*RagA*$^{GTP/\Delta}$ (fasted n=3, fed n=3). Statistical significance was calculated using the LIMMA program as indicated in Materials and Methods. * indicates a statistically significant difference between control and Li-*RagA*$^{GTP/\Delta}$ mice in fasted conditions. r, Enrichment of gene sets (GSEA) related to mitochondrial and peroxisomal β-oxidation in livers from fasted Li-*RagA*$^{GTP/\Delta}$ (n=3) vs control (n=3) mice from Figure 7c. NES: normalized enrichment score; FDR: false discovery rate. For f, and h-m, statistical significance was calculated by using 2-way ANOVA with Sidak's multiple comparison correction. In all panels, horizontal lines indicate the mean and error bars depict Standard deviation (SD).

Supp. Figure 8

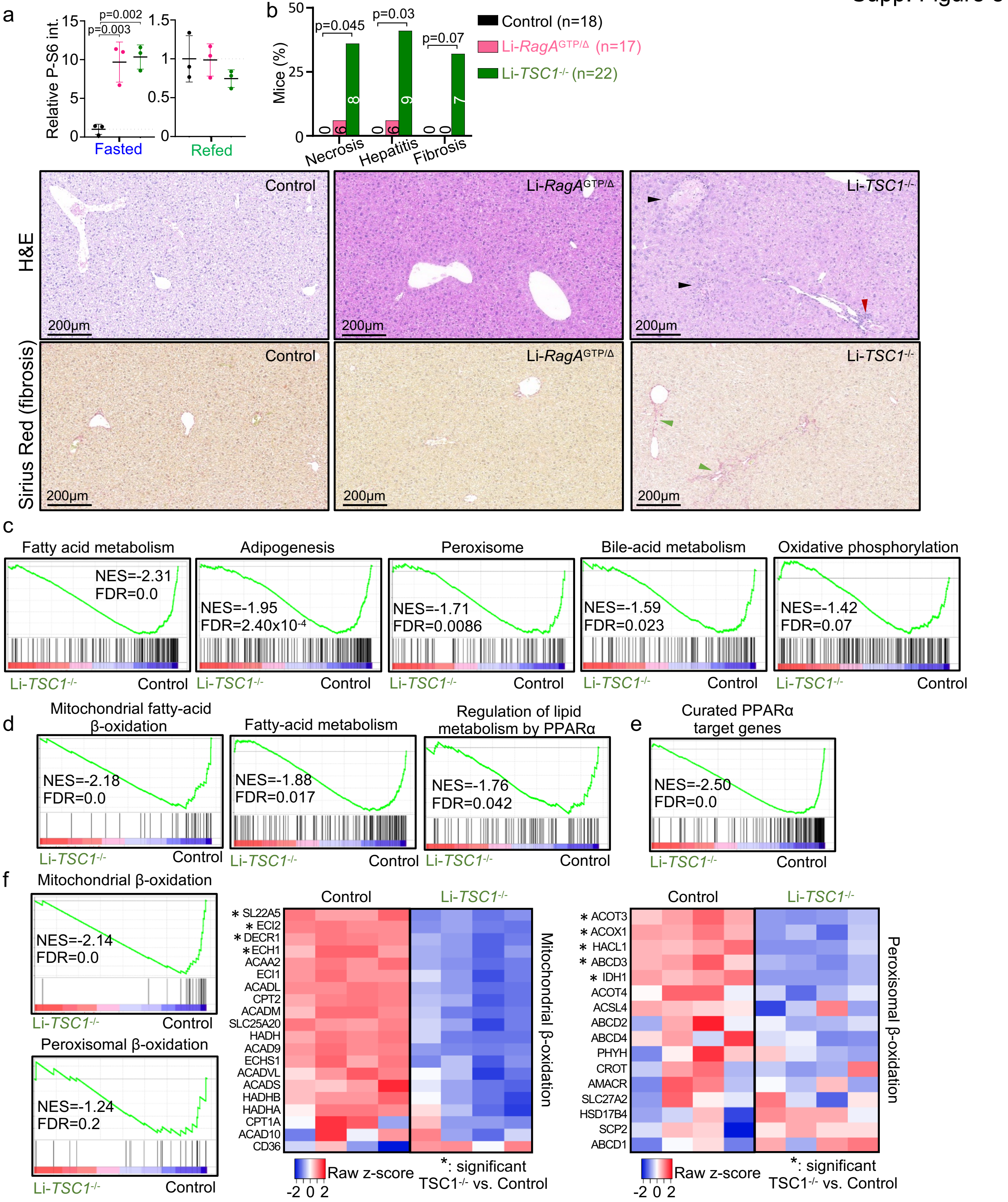

Supplementary Fig. 8 | Related to Figure 8. a, Quantification of P-S235/236-S6 intensity from Figure 8e. Statistical significance was calculated by using 1way ANOVA with Sidak's multiple comparison correction. b,10- to 39-week-old control (n=18), Li-*RagA*$^{GTP/\Delta}$ (n=17) and Li-*TSC1*$^{-/-}$ (n=22) mice were sacrificed and liver damage was evaluated (top). Representative hepatic hematoxylin and eosin (H&E) and Sirius Red staining of control, Li-*RagA*$^{GTP/\Delta}$ and Li-*TSC1*$^{-/-}$ mice (bottom). Black arrowheads indicate necrotic areas, red arrowheads indicate the presence of inflammatory foci and green arrowheads indicate areas of fibrosis denoted by the observation of collagen fibers in red color. Statistical significance was calculated by using chi-square test. c-e, Enrichment of gene sets (GSEA) related to indicated signatures from Hallmarks (c), Reactome (d), and our curated PPARα targets (e) in the livers from Li-*TSC1*$^{-/-}$ (n=4) vs control (n=4) mice from Figure 8f. NES: normalized enrichment score; FDR: false discovery rate. f, Enrichment of gene sets (GSEA) related to mitochondrial and peroxisomal β-oxidation in livers from fasted Li-*TSC1*$^{-/-}$ (n=4) vs control (n=4) mice from Figure 8f (left). NES: normalized enrichment score; FDR: false discovery rate. Heat map of proteins related to mitochondrial and peroxisomal β-oxidation detected in the proteomics analysis of Figure 8f (right). Control (n=4) and Li-*TSC1*$^{-/-}$ (n=4). Statistical significance was calculated using the LIMMA program as indicated in Materials and Methods. * indicates a statistically significant difference between control and Li-*TSC1*$^{-/-}$ mice and error bars depict Standard deviation (SD).

Classical TOP		
EEF1A1	RPL27	RPS14
EEF1B2	RPL27A	RPS15
EEF1D	RPL28	RPS15A
EEF1G	RPL29	RPS16
EEF2	RPL3	RPS17
EIF3A	RPL30	RPS18
EIF3E	RPL31	RPS19
EIF3F	RPL32	RPS2
EIF3H	RPL34	RPS20
EIF4B	RPL35	RPS21
FAU	RPL35A	RPS23
GNB2L1	RPL36	RPS24
HNRNPA1	RPL36A	RPS25
NAP1L1	RPL37	RPS26
PABPC1	RPL37A	RPS27
RPL10	RPL38	RPS27A
RPL10A	RPL39	RPS28
RPL11	RPL3L	RPS29
RPL12	RPL4	RPS3
RPL13	RPL41	RPS3A
RPL13A	RPL5	RPS4X
RPL14	RPL6	RPS4Y1
RPL15	RPL7	RPS4Y2
RPL17	RPL7A	RPS5
RPL18	RPL8	RPS6
RPL18A	RPL9	RPS7
RPL19	RPLP0	RPS8
RPL21	RPLP1	RPS9
RPL22	RPLP2	RPSA
RPL23	RPS10	TPT1
RPL23A	RPS11	VIM
RPL24	RPS12	
RPL26	RPS13	

PPARa target genes - Curated					
ADASA	ACOT6	CROT	GPD1	PECR	TECR
ABCA1	ACOT7	CYP4A10	GPD2	PERRO	UCP2
ABCB4	ACOT8	CYP4A12A	GYK	PEX1	VLDLR
ABCB6	ACOX1	CYP4A14	GYS2	PEX11A	
ABCC3	ACSL1	CYP4A31	HACL1	PEX13	
ABCD1	ACSL5	CYP4A32	HADH	PEX14	
ABCD2	ACSM3	CYP4F15	HADHA	PEX16	
GENE	ACSM5	CYP7A1	HADHB	PEX19	
ABCD3	ADH1	CYP8YB1	HMGCL	PEX26	
ABCG2	ADH4	DBI	HMGCS2	Pex3	
ABCG5	AGPAT3	DECR1	HSD17B10	PEX5	
ABCG8	AGPAT6	DECR2	HSD17B12	PLIN2	
ACAA1A	AGPAT9	DGAT1	HSD17B4	PLIN4	
ACAA1B	ALAD	DHRS3	HSDL2	PLIN5	
ACAA2	ALDH1A1	DHRS4	KLF10	PMPT	
ACACB	ALDH3A2	ECH1	KLF11	PNPLA2	
ACAD10	ALDH9A1	EHHADH	LIPA	PPARA	
ACAD11	AQP3	ELOV1	LIPC	RDH5	
ACAD8	AQP9	ELOV2	LIPE	RETSAT	
ACADL	ATFDH	ELOV3	LIPG	SCARB1	
ACADM	BBOX1	ELOV5	LIPIN2	SCD1	
ACADS	BDH1	ELOV6	LPCAT3	SCD2	
ACADVL	CD36	ETFB	ME1	SLC10A1	
ACAT1	CELIA	FABP1	MGLL	SLC16A1	
ACI1	CIDEA	FABP2	MLYCD	SLC22A5	
ACI2	CIDEC	FABP4	MTTP	SLC25A10	
ACOT1	COX6B2	FADS2	NCEH1	SLC25A11	
ACOT12	CPOX	FBP1	NR1H4	SLC25A13	
ACOT13	CPT1A	FBP2	PCTP	SLC25A20	
ACOT2	CPT1B	FITM1	PCX	SLC25A42	
ACOT3	CPT2	G0S2	PDK1	SLC27A2	
ACOT4	CRAT	GALNT2	PDK4	SLC27A4	
ACOT5	CREB3L3	GPAM	PECI	SLC2A2	

Primer list		
PPARα	Forward Primer	AGAGCCCCATCTGTCCTCTC
	Reverse Primer	ACTGGTAGTCTGCAAAACCAAA
PPARγ	Forward Primer	ACTGCCTATGAGCACTTCAC
	Reverse Primer	CAATCGGATGGTTCTTCGGA
PPARβ/δ	Forward Primer	TTGAGCCCAAGTTCGAGTTTG
	Reverse Primer	CGGTCTCCACACAGAATGATG
PCG1α	Forward Primer	CAGCAAAAGCCACAAAGACG
	Reverse Primer	AGTTCCAGAGAGTTCCACAC
ACAT1	Forward Primer	ATGCATCCAAACCCACTTTG
	Reverse Primer	TGCCCATGTAGACTTCCTTCA
HMGCS2	Forward Primer	ATACCACCAACGCCTGTTATGG
	Reverse Primer	CAATGTCACCACAGACCACCAG
CPT1α	Forward Primer	CCATGAAGCCCTCAAACAGATC
	Reverse Primer	ATCACACCCACCACCACGATA
ACADM	Forward Primer	GATCGCAATGGGTGCTTTTGATAGAA
	Reverse Primer	AGCTGATTGGCAATGTCTCCAGCAAA
ACADL	Forward Primer	GTAGCTTATGAATGTGTGCAACTC
	Reverse Primer	GTCTTGCGATCAGCTCTTTCATTA
ARG	Forward Primer	GCTCCAAGCCAAAGTCCTTA
	Reverse Primer	GACATCAACAAAGGCCAGGT
ASS1	Forward Primer	GCATTGACATCGTGGAGAAC
	Reverse Primer	TCCATCGTGAAGGCCTCTAT
ASL	Forward Primer	GACCTCAGGCTGTGGATGAG
	Reverse Primer	AATCTCGTGTCAGCGCAAC
PMP70	Forward Primer	GCACAAGCTAGTCCCTTGGT
	Reverse Primer	GCTCTTTCCACAGCCGTTTG
CAT	Forward Primer	GTGCCCCCAACTATTACCCC
	Reverse Primer	TCTCACACAGGCGTTTCCTC
PEX3	Forward Primer	CCCTAGGCAACCCACACAA
	Reverse Primer	AATGGTACTGTCGCCGAGC
PEX16	Forward Primer	GGCTACTGTCTGGTGTGGTAGATA
	Reverse Primer	TAGTCCATGAGCGGCCTTGC
PEX19	Forward Primer	CAAGTCGGAGGCAGTAAGATGG
	Reverse Primer	GAGGGTTTGGCTTTGTCGAAAT
ACOX1	Forward Primer	GAATTTGGCATCGCAGACCC
	Reverse Primer	TGCCCAAGTGAAGGTCCAAA
ACOX2	Forward Primer	GGTGAGTCAAGAGTTTAGTCCCA
	Reverse Primer	TTGGCTTCCTTTAGGGTCTGC
β-Actin	Forward Primer	GGCACCACACCTTCTACAATG
	Reverse Primer	GTGGTGGTGAAGCTGTAGCC
SREBF	Forward Primer	GGTTTTGAACGACATCGAAGA
	Reverse Primer	CGGGAAGTCACTGTCTTGGT
ACACA	Forward Primer	ACCAGCACTCCCGATTCATA
	Reverse Primer	TTTACTAGGTGCAAGCCAGACAT
ACLY	Forward Primer	CCCTGAAGGAGCATGAGGT
	Reverse Primer	AATGGCCGTCATGTGAGTTT
FASN	Forward Primer	CAAGCAGGCACACACAATG
	Reverse Primer	ACCATGCTGTAGCCCAGAAG
G6P	Forward Primer	GAAGGCCAAGAGATGGTGTGA
	Reverse Primer	TGCAGCTCTTGCGGTACATG
PEPCK	Forward Primer	CGATGACATCGCCTGGATGA
	Reverse Primer	TCTTGCCCTTGTGTTCTGCA
PYGL	Forward Primer	TGCTTTGGATAAGAAGGGGTATGAGGC
	Reverse Primer	TTGAAGAGGTCTGGCTGATTGGGAG
