# Supplementary material for: Limited survival and impaired hepatic fasting metabolism in mice with constitutive Rag GTPase signaling
Source: Nat Commun. 2021 Jun 16;12:3660. doi: 10.1038/s41467-021-23857-8 (PMC8209044; doi:10.1038/s41467-021-23857-8)
Supplement: Supplementary file 2 — Description of Additional Supplementary Files [file 41467_2021_23857_MOESM2_ESM.pdf]

Description of Additional Supplementary Files

Title: Supplementary Data 1

Description: Proteomics dataset of liver samples from full-body $RagA^{+/+}$ and $RagA^{GTP/\Delta}$ mice. Samples were collected upon overnight fasting and in *ad libitum* fed conditions.

Title: Supplementary Data 2

Description: Proteomics dataset of liver samples from Control and Li-$RagA^{GTP/\Delta}$ mice. Samples were collected upon overnight fasting with and without refeeding.

Title: Supplementary Data 3

Description: Proteomic dataset of liver samples from fasted Control and Li-$TSC1^{-/-}$ mice.
