# Supplementary material for: Limited survival and impaired hepatic fasting metabolism in mice with constitutive Rag GTPase signaling
Source: Nat Commun. 2021 Jun 16;12:3660. doi: 10.1038/s41467-021-23857-8 (PMC8209044; doi:10.1038/s41467-021-23857-8)
Supplement: Supplementary file 6 — Reporting Summary [file 41467_2021_23857_MOESM6_ESM.pdf]

nature research

Corresponding author(s): Alejo Efeyan
Last updated by author(s): May 10, 2021

Reporting Summary

Nature Research wishes to improve the reproducibility of the work that we publish. This form provides structure for consistency and transparency in reporting. For further information on Nature Research policies, see our Editorial Policies and the Editorial Policy Checklist.

Statistics

For all statistical analyses, confirm that the following items are present in the figure legend, table legend, main text, or Methods section.

n/a	Confirmed	
☐	☒	The exact sample size (*n*) for each experimental group/condition, given as a discrete number and unit of measurement
☐	☒	A statement on whether measurements were taken from distinct samples or whether the same sample was measured repeatedly
☐	☒	The statistical test(s) used AND whether they are one- or two-sided<br>*Only common tests should be described solely by name; describe more complex techniques in the Methods section.*
☒	☐	A description of all covariates tested
☐	☒	A description of any assumptions or corrections, such as tests of normality and adjustment for multiple comparisons
☐	☒	A full description of the statistical parameters including central tendency (e.g. means) or other basic estimates (e.g. regression coefficient) AND variation (e.g. standard deviation) or associated estimates of uncertainty (e.g. confidence intervals)
☐	☒	For null hypothesis testing, the test statistic (e.g. *F*, *t*, *r*) with confidence intervals, effect sizes, degrees of freedom and *P* value noted<br>*Give P values as exact values whenever suitable.*
☒	☐	For Bayesian analysis, information on the choice of priors and Markov chain Monte Carlo settings
☐	☒	For hierarchical and complex designs, identification of the appropriate level for tests and full reporting of outcomes
☒	☐	Estimates of effect sizes (e.g. Cohen's *d*, Pearson's *r*), indicating how they were calculated

Our web collection on statistics for biologists contains articles on many of the points above.

Software and code

Policy information about availability of computer code

Data collection	Odyssey Infrared Imaging System (Application software version 3.0.30) LI-COR Biosciences - v10, AxioScan Z1, Zeiss - Zen 2.3 Blue edition Software (Zeiss) - Mouse Densitometer (GE Lunar co, Madison, WI, USA), software version 1.46 - Microview 2.5.0 software (GEHC; London, Canada) - 3D Slicer 4.10.1 software https://www.slicer.org/ - Thermo Xcalibur 4.0.27.13 software - Thermo TraceFinder 3.3 SP1 software
Data analysis	GraphPad v8.3. - For chi-square: http://vassarstats.net/newcs.html - For heatmap representation: http://www.heatmapper.ca/expression/ (PMID: 27190236) - For proportional diagram representation: https://www.biovenn.nl/index.php (PMID: 18925949) - For proportional diagram statistic calculations: http:// nemates.org/MA/progs/overlap_stats.html - MaxQuant (v 1.6.10.43) - Bayes statistics Limma 3.46.0 - GSEA (v4.1.0) - ImageJ (1.52a)

For manuscripts utilizing custom algorithms or software that are central to the research but not yet described in published literature, software must be made available to editors and reviewers. We strongly encourage code deposition in a community repository (e.g. GitHub). See the Nature Research guidelines for submitting code & software for further information.

Data

Policy information about availability of data

All manuscripts must include a data availability statement. This statement should provide the following information, where applicable:
- Accession codes, unique identifiers, or web links for publicly available datasets
- A list of figures that have associated raw data
- A description of any restrictions on data availability

The metabolomics data is uploaded in EMBL-EBI Metabolights, database https://www.ebi.ac.uk/metabolights/ with the identifier MTBLS2397. The mass spectrometry proteomics data have been deposited to the ProteomeXchange Consortium (http://proteomecentral.proteomexchange.org/cgi/GetDataset?

ID=PXD023735). The authors declare that other data supporting the findings of this study are available within the article and its Supplementary information files or are available upon reasonable request to the corresponding author.

Field-specific reporting

Please select the one below that is the best fit for your research. If you are not sure, read the appropriate sections before making your selection.

☒ Life sciences ☐ Behavioural & social sciences ☐ Ecological, evolutionary & environmental sciences

For a reference copy of the document with all sections, see nature.com/documents/nr-reporting-summary-flat.pdf

Life sciences study design

All studies must disclose on these points even when the disclosure is negative.

Sample size	No sample size calculation was performed, as the magnitude of the effect sizes were unknown. As reference, the sample sizes were guided on the basis of similar published studies (PMID: 24768164; PMID: 21179166).<br>Details on sample size of all experiments are provided in the Methods section and figure legends.
Data exclusions	No data were excluded unless as determined by technical problems.
Replication	Experiments were repeated at least twice. All replication attempts were successful.
Randomization	Mice were randomly assigned to different treatments.
Blinding	Experiments were not blinded as full-body mutant mice were sometimes distinguishable by eye.

Reporting for specific materials, systems and methods

We require information from authors about some types of materials, experimental systems and methods used in many studies. Here, indicate whether each material, system or method listed is relevant to your study. If you are not sure if a list item applies to your research, read the appropriate section before selecting a response.

Materials & experimental systems

n/a	Involved in the study	
☐	☒	Antibodies
☒	☐	Eukaryotic cell lines
☒	☐	Palaeontology and archaeology
☐	☒	Animals and other organisms
☒	☐	Human research participants
☒	☐	Clinical data
☒	☐	Dual use research of concern

Methods

n/a	Involved in the study	
☒	☐	ChIP-seq
☒	☐	Flow cytometry
☒	☐	MRI-based neuroimaging

Antibodies

Antibodies used

P-T389-S6K1 (#9234) CST
S6K1 (#2708) CST
P-S235/236-S6 (#2211) CST
S6 (#2217) CST
P-T37/46-4EBP1 (#2855) CST
4EBP1 (#9644) CST
Catalase (#14097S) CST
RagA (#4375) CST
RagC (#9480) CST
P-S473-AKT (#4060) CST
P-T308-AKT (#2965) CST
AKT (#4691) CST
P-T246-PRAS40 (#2997) CST
PRAS40 (#2691) CST
P-S9-GSK3β (#9336) CST
GSK3β (#9315) CST
P-T24/32-FOXO1/3 (#9464) CST
FOXO1 (#2880) CST
P-S588-AS160 (#8730) CST
AS160 (#2670) CST
Pex14 (#10594-1-AP) Proteintech

	Pex16 (#14816-1-AP) Proteintech<br>Pex19 (#14713-1-AP) Proteintech<br>PMP70 (#SAB4200181) Sigma<br>Thiolase (#HPA007244) Sigma<br>β-actin (#A1978) Sigma<br>Vinculin (#V9131) Sigma
Validation	P-T389-S6K1 (#9234) CST - Used for WB in mouse tissues as validated by the company and by users (cited 968 times)<br>S6K1 (#2708) CST - Used for WB in mouse tissues as validated by the company and by users (cited 842 times)<br>P-S235/236-S6 (#2211) CST - Used for WB in mouse tissues as validated by the company and by users (cited 852 times)<br>S6 (#2217) CST - Used for WB in mouse tissues as validated by the company and by users(cited 1143 times)<br>P-T37/46-4EBP1 (#2855) CST - Used for WB in mouse tissues as validated by the company and by users (cited 829 times)<br>4EBP1 (#9644) CST - Used for WB in mouse tissues as validated by the company and by users (cited 533 times)<br>Catalase (#14097S) CST - Used for WB in mouse tissues as validated by the company and by users (cited 20 times)<br>RagA (#4375S) CST - Used for WB in mouse tissues as validated by the company and by users (cited 30 times)<br>RagC (#9480) CST - Used for WB in mouse tissues as validated by the company and by users (cited 8 times)<br>P-S473-AKT (#4060) CST - Used for WB in mouse tissues as validated by the company and by users (cited 4534 times)<br>P-T308-AKT (#2965) CST - Used for WB in mouse tissues as validated by the company and by users (cited 562 times)<br>AKT (#4691) CST - Used for WB in mouse tissues as validated by the company and by users (cited 2115 times)<br>P-T246-PRAS40 (#2997) CST - Used for WB in mouse tissues as validated by the company and by users (cited 124 times)<br>PRAS40 (#2691) CST - Used for WB in mouse tissues as validated by the company and by users (cited 90 times)<br>P-S9-GSK3β (#9336) CST - Used for WB in mouse tissues as validated by the company and by users (cited 582 times)<br>GSK3β (#9315) CST - Used for WB in mouse tissues as validated by the company and by users(cited 725 times)<br>P-T24/32-FOXO1/3 (#9464) CST - Used for WB in mouse tissues as validated by the company and by users (cited 225 times)<br>FOXO1 (#2880) CST - Used for WB in mouse tissues as validated by the company and by users (cited 482 times)<br>P-S588-AS160 (#8730) CST - Used for WB in mouse tissues as validated by the company and by users (cited 30 times)<br>AS160 (#2670) CST - Used for WB in mouse tissues as validated by the company and by users (cited 36 times)<br>Pex14 (#10594-1-AP) Proteintech - Used for WB in mouse tissues as validated by the company and by users (cited 26 times)<br>Pex16 (#14816-1-AP) Proteintech - Used for WB in mouse tissues as validated by the company and by users (cited 11 times)<br>Pex19 (#14713-1-AP) Proteintech - Used for WB in mouse tissues as validated by the company and by users (cited 1 times)<br>PMP70 (#SAB4200181) Sigma - Used for WB in mouse tissues as validated by the company and by users (cited 36 times)<br>Thiolase (#HPA007244) Sigma - Used for WB in mouse tissues as validated by the company and by users (cited 8 times)<br>β-actin (#A1978) Sigma - Used for WB in mouse MEFs as validated by the company and by users (cited 2809 times)<br>Vinculin (#V9131) Sigma - Used for WB in mouse tissues as validated by the company and by users (cited 1121 times)

Animals and other organisms

Policy information about studies involving animals; ARRIVE guidelines recommended for reporting animal research

Laboratory animals	Mus musculus, C57BL/6 and 50:50 C57BL/6:129sv, males and females, from E13.5 to 2.9 years old.
Wild animals	not used
Field-collected samples	no field-collected samples
Ethics oversight	All animal procedures carried out at the CNIO were performed according to protocols approved by the CNIO-ISCIII Ethics Committee for Research and Animal Welfare (CEIyBA) and the Autonomous Community of Madrid (CAM). Protocol IDs: PROEX285/15 and PROEX15/18.

Note that full information on the approval of the study protocol must also be provided in the manuscript.
